# Comparing efficacy and safety of first-line treatment of metastatic renal cell carcinoma: A Bayesian network meta-regression analysis

**DOI:** 10.3389/fonc.2023.1072634

**Published:** 2023-02-22

**Authors:** Suyang Qin, Zhiyuan Xv, Xi Chen, Shurui Wang, Hai Lu, Jiaqi Li, Xinglin Guo, Jinghua Yang, Chengjiang Liu, Yaoguang Wang, Hongwu Wang

**Affiliations:** ^1^ First Teaching Hospital of Tianjin University of Traditional Chinese Medicine, National Clinical Research Center for Chinese Medicine Acupuncture and Moxibustion, Tianjin, China; ^2^ School of Health, Brooks College, Sunnyvale, CA, United States; ^3^ Department of Epidemiology and Statistics, School of Public Health, Medical College, Zhejiang University, Hangzhou, China; ^4^ School of Nursing, Chinese Academy of Medical Sciences & Peking Union Medical College, Beijing, China; ^5^ Nanjing Drum Tower Hospital, The Affiliated Hospital of Nanjing University Medical School, Nanjing, China; ^6^ Department of Gastroenterology, Anhui Medical University, Hefei, China; ^7^ School of Health Sciences and Engineering, Tianjin University of Traditional Chinese Medicine, Tianjin, China

**Keywords:** metastatic renal cell carcinoma, network meta-regression analysis, immune checkpoint inhibitors, tyrosine kinase inhibitors, efficacy

## Abstract

**Background:**

This Bayesian network meta-regression analysis provides a head-to-head comparison of first-line therapeutic immune checkpoint inhibitors (ICI) and tyrosine kinase inhibitors (TKI) combinations for metastatic renal cell carcinoma (mRCC) using median follow-up time as covariate.

**Methods:**

We searched Six databases for a comprehensive analysis of randomised clinical trials (RCTs). Comparing progression free survival (PFS) and overall survival (OS) of different interventions at the same time node by Bayesian network meta-analysis. Bayesian network meta-regression analysis was performed on objective response rate (ORR), adverse events (AEs) (grade ≥ 3) and the hazard ratios (HR) associated with PFS and OS, with the median follow-up time as the covariate.

**Results:**

Eventually a total of 22 RCTs reporting 11,090 patients with 19 interventions. Lenvatinib plus Pembrolizumab (LenPem) shows dominance of PFS, and Pembrolizumab plus Axitinib (PemAxi) shows superiority in OS at each time point. After meta-regression analysis, for HRs of PFS, LenPem shows advantages; for HRs of OS, PemAxi shows superiority; For ORR, LenPem provides better results. For AEs (grade ≥ 3), Atezolizumab plus Bevacizumab (AtezoBev) is better.

**Conclusion:**

Considering the lower toxicity and the higher quality of life, PemAxi should be recommended as the optimal therapy in treating mRCC.

**Systematic review registration:**

https://www.crd.york.ac.uk/prospero/, identifier CRD4202236775.

## 1 Introduction

According to the latest statistics, renal cancer is the sixth most common malignant tumor in men and the ninth in women. It is estimated that 79,000 new diagnoses will be determined in the United States in 2022, resulting in 13,920 deaths (1). Among them, 80% are renal cell carcinoma (RCC) (2). In the past 20 years, the incidence rate of RCC has continued to rise (3). In addition, in approximately 35% of cases, metastatic RCC (mRCC) was first diagnosed (4).

Since 2006, treatment of mRCC in the first instance has gradually changed from interleukin-2 (IL-2) and interferon-α (IFN-α) with serious toxic and side effects to the treatment of tyrosine kinase inhibitors (TKI) Sunitinib (Suni) and Pazopanib (Pazo) (5). Over the ensuing years, with the advent of TKI and mamman target of rapamycin (mTOR) inhibitors, survival rates for those with mRCC have improved dramatically (6). Simultaneously in 2016, the immune checkpoint inhibitors (ICI) has achieved significant effect in the treatment of mRCC, which could be regarded as a milestone (7). Recently, ICI-TKI and ICI-ICI have demonstrated remarkable efficacy in patients with mRCC. Phase 3 CLEAR Trial demonstrated that the objective response rate (ORR), OS and progression-free survival (PFS) of first-line treatment Lenvatinib plus Pembrolizumab (LenPem) were significantly higher than that of Suni (8). First-line treatment Nivolumab plus Cabozantinib (NivoCabo), when compared with Suni, showed significantly improved OS, PFS, and ORR in phase 3 CheckMate 9ER (9). A four-year-long study indicated that in intermediate/poor-risk patients with IMDC, Nivolumab plus lpilimumab (Nivolpi) had better OS, PFS, and ORR than Suni, according to the phase 3 CheckMate 214 trial (10). According to the phase 3 KEYNOTE-426 trial, significant improvement in PFS, OS and ORR for all IMDC risk group patient treated with Pembrolizumab plus Axitinib (PemAxi) versus Suni (11). The phase 3 JAVELIN Renal 101 trial shows that PFS was significantly higher in Avelumab plus Axitinib (AveAxi) than in Suni among all patients in the IMDC risk group (12).

Notwithstanding, there is still no direct comparison between ICI-ICI and ICI-TKI for certain reasons, a head-to-head comparison of different ICI and TKI combinations remains paucity. Naturally, network meta-analysis (NMA) acts as an indispensable bridge to materialize the indirect comparisons. However, previous NMAs did not compare PFS and OS of different interventions at the same time node, resulting potential bias as treatment period might be the confounding factor. Moreover, no studies have focused on the effect of different median follow-up times on ORR and AEs (grade ≥ 3) of different interventions.

Hence, based on this study, we performed a Bayesian NMA to investigate the effectiveness of different combinations of ICI and TKI at each time node and Bayesian network meta-regression analysis adjusting follow-up time using hazard ratios from kaplan meier curve as primitive data to provide more precise evidence for practice in clinical settings.

## 2 Methods

This NMA was guided by the PRISMA guideline (Preferred Reporting Items for Systematic Reviews and Meta-analysis) (13).

### 2.1 Search strategy

We searched Google Scholars, Cochrane Library (CENTRAL), PubMed, Scopus and Embase from inception to August 28, 2022, with the following Mesh terms: (“kidney carcinoma” OR “Carcinoma, Renal Cell” OR “renal cell cancer” OR “renal cell carcinoma” OR “kidney cancer” OR “renal carcinoma” OR “renal cancer”) AND (“randomized” OR “Allocation, Random” OR “Randomization”).

### 2.2 Selection criteria

The following criteria were used to determine inclusion: (1) The mRCC patient population; (2) no history of systemic therapy; (3) first-line pairwise comparisons treatments; (4) reporting PFS, OS, ORR, or AEs (grade ≥ 3). (5) randomized controlled trials (RCTs).

Below are the exclusion criteria: (1) observational studies, letters, review, or conference abstract; (2) single-arm design studies; (3) animal studies or research *in vitro*; (4) interferon as control arm (in light of the widespread acceptance of TKI as a standard of care); (5) non-Chinese and non-English literature.

### 2.3 Data extraction and quality assessment

In the included studies, data were independently extracted by two investigators (SQ and ZX) and used the Cochrane Risk of Bias 2.0 tool, assessed the risk of bias for each included RCT by Review Manager 5.3, any discrepancy was arbitrated by the senior reviewer (XC). Variables recorded include: name of the first author, country, publication year, number of patients, condition, therapeutic drugs, treatment dosage, median follow-up time, treatment level, ORR, AEs (grade ≥ 3), and the hazard ratios (HR) and 95% confidence intervals (CI) associated with PFS and OS. Subsequently, the data regarding to PFS and OS at 3, 6, 12, 18, 24, 30, 36 month were harvested from kaplan meier curve by Getdata 2.26.

### 2.4 Data analysis

For PFS and OS at each time point, the Bayesian NMA was conducted with STATA 17.0 MP to directly and indirectly compare multiple treatments. After evaluating OS and PFS at each time point with odds ratio (OR) and 95% Cl, treatment ranking was performed conducting the surface under the cumulative ranking curve (SUCRA) values, however, whether the effect size between any pair reached the significance was determined by net-league table, which was also called matrix in algebra. Inconsistency and consistency tests were performed to examine the existence of inconsistency. Publication bias was assessed by funnel plots as well.

HRs for OS and PFS, Napierian Logarithm HR (lnHR) and standard error of lnHR (selnHR) for each study were calculated by STATA 17.0 MP. For the ORR and AEs (grade ≥ 3), conventional meta-analyses were conducted by STATA 17.0 MP to generate Napierian Logarithm odds ratios (lnOR) and standard error of lnOR (selnOR) for each study. Subsequently these data (lnHR and selnHR for OS and PFS, lnOR and selnOR for ORR and AEs, respectively) were input into Rstudio 4.1.2 by “gemtc” package to conduct Bayesian NMA. if *I^2^* <50% and *p*>0.01, fixed effect model would be implemented; if 50%<*I^2^* <75%, random effect model was carried out; if *I^2^* >75%, Galbraith plot would be drawn to preclude the studies outside the outlines. Markov-chain Monte Carlo (MCMC) was used to obtain posterior distributions, with 20,000 burn-ins and 150,000 iterations of 4 each chain and a thinning interval of 10 for each outcome. Brooks-Gelman-Rubin diagnostics and Trace plots were used to evaluate and visualize the convergence of the model over iterations. Matrices were also generated by Rstudio 4.1.2.

Finally, we conducted sensitivity analyses, using median follow-up time as a covariate to perform meta-regression analyses to eliminate potential confounding factors.

## 3 Results

### 3.1 Characteristics of the included studies

**Figure 1:**
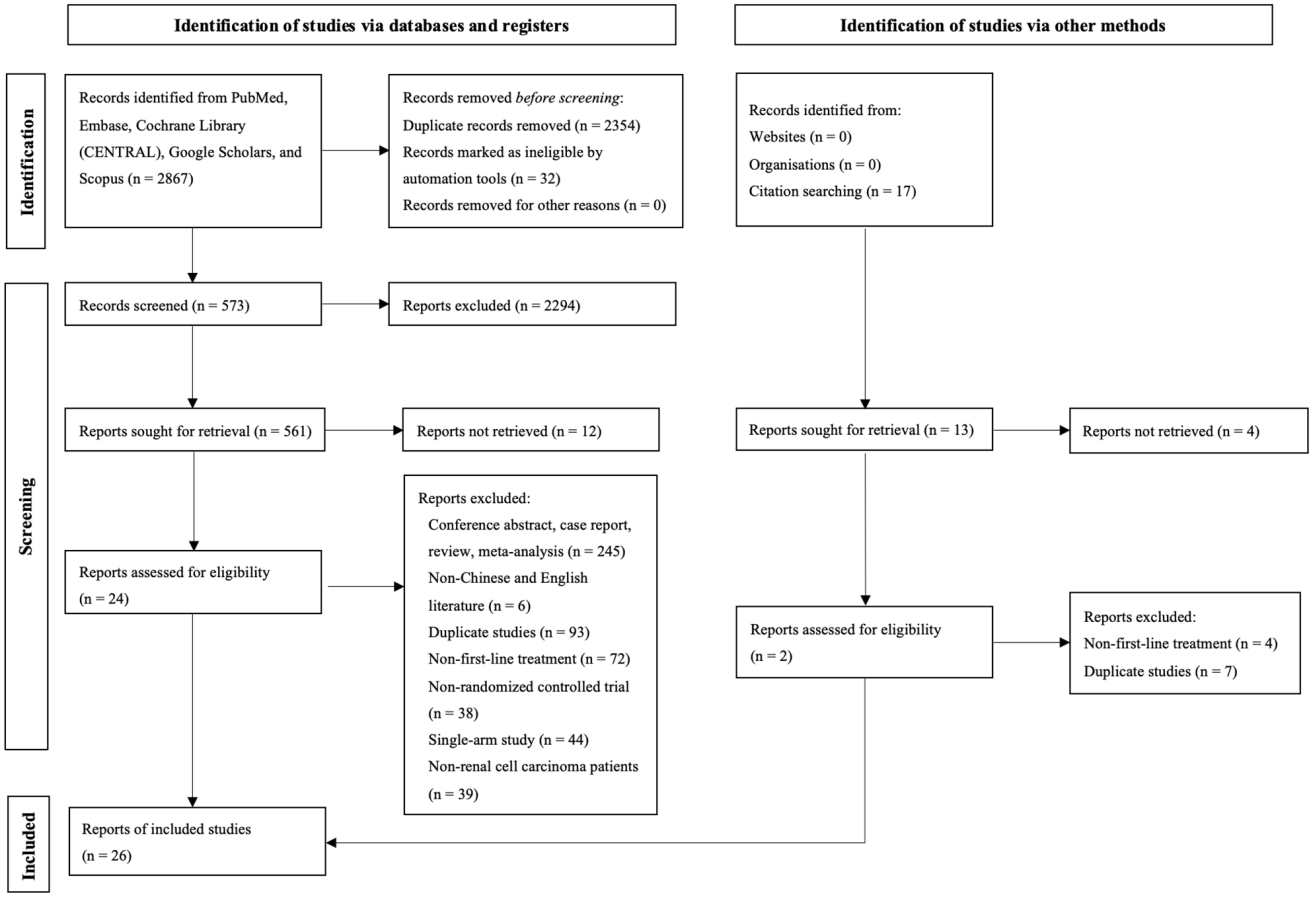
This diagram shows the PSRISMA flow diagram for study search and selection (updated in 2020). PSRISMA, Preferred Reporting Items for Systematic Reviews and Meta-Analyses; CENTRAL, Cochrane Central Register of Controlled Trials; Embase, Excerpta Medica database.

During the initial search, we obtained 5253 publications. As a result of eliminating duplicates and screening titles and abstracts, 537 studies were eligible to be reviewed in full, and 26 studies finally met our criteria (**Figure 1**) (8–12, 14–34) Eventually a total of 22 RCTs reporting 11,090 patients with 19 interventions, namely Suni, Bevacizumab (Bev), Cabozantinib (Cabo), Pazo, Savolitinib (Savo), Sorafenib (Sora), Axitinib (Axi), Anlotinib (Anlo), Nivolumab (Nivo), Atezolizumab (Atezo), PemAxi, Atezolizumab plus Bevacizumab (AtezoBev), AveAxi, LenPem, NivoCabo, NivoIpi, Lenvatinib plus Everolimus (LenEvero), Pazopanib plus Everolimus (PazoEvero), and IMA901 plus Sunitinib (IMA901Suni). A detailed description of the included studies can be found in **Table 1**. All patients included in the study were untreated patients with mRCC, and a median follow-up period of 6.4 months to 55 months was reported in the study. The assessment of risk of bias is presented in **Supplementary Figures 1A and B**.

**Table 1:** Characteristics of first-line systemic therapy for metastatic renal cell carcinoma studies included in the network meta-analysis.

Study	Year	Condition	Treatment	Sample size	Follow-up (month)	Dosage	Outcomes	Treatment level
Rini (14)	2016	metastatic renal cell carcinoma	Sunitinib	204	33.3	50 mg Qd	PFS, OS, grade ≥ 3 AEs	first-line therapy
			IMA901+Sunitinib	135		Sunitinib 50 mg Qd and IMA901 4.13 mg intr		
Yang (15)	2003	metastatic renal cell carcinoma	Bevacizumab	39	27	3 mg/kg Q2W	PFS, OS, ORR	first-line therapy
			Placebo	40		matching Placebo		
Choueiri (16)	2018	metastatic renal cell carcinoma	Cabozantinib	79	21.4	60 mg Qd	PFS, OS, ORR, grade ≥ 3 AEs	first-line therapy
			Sunitinib	79		50 mg Qd		
Tamada (17)	2022	metastatic renal cell carcinoma	Pembrolizumab+Axitinib	44	29.5	Pembrolizumab 200 mg Q3W + Axitinib 5 mg Bid	PFS, ORR, grade ≥ 3 AEs	first-line therapy
			Sunitinib	50		50 mg Qd		
Sternberg (18)	2010	metastatic renal cell carcinoma	Pazopanib	290	6.4	800 mg Qd	PFS, ORR, grade ≥ 3 AEs	first-line therapy
			Placebo	145		matching Placebo		
Sheng (19)	2020	metastatic renal cell carcinoma	Sunitinib	100	29.5	50 mg Qd	PFS, OS, ORR, grade ≥ 3 AEs	first-line therapy
			Pazopanib	109		800 mg Qd		
Motzer (20)	2013	metastatic renal cell carcinoma	Pazopanib	557	29.5	801 mg Qd	PFS, ORR, grade ≥ 3 AEs	first-line therapy
			Sunitinib	553		50 mg Qd		
Motzer (21)	2014	metastatic renal cell carcinoma	Pazopanib	557	29.5	800 mg QD	OS	first-line therapy
			Sunitinib	553		50 mg Qd		
Cirkel (22)	2016	metastatic renal cell carcinoma	Pazopanib+Everolimus	52	20.2	Pazopanib 800mg Qd + Everolimus 10 mg Qd	PFS, grade ≥ 3 AEs	first-line therapy
			Pazopanib	49		800mg Qd		
Powles (11)	2020	advanced renal cell carcinoma	Pembrolizumab+Axitinib	432	30.6	200 mg intr Q3W	PFS, OS, ORR, grade ≥ 3 AEs	first-line therapy
			Sunitinib	429		5 mg Axitinib Qd or 50 mg Sunitinib Qd		
Choueiri (23)	2020	metastatic renal cell carcinoma	Savolitinib	33	20.6	600mg Qd	PFS, OS, ORR, grade ≥ 3 AEs	first-line therapy
			Sunitinib	27		50 mg Qd		
Escudier (24)	2007	advanced renal cell carcinoma	Sorafenib	451	6.6	400 mg Bid	PFS, OS, ORR, grade ≥ 3 AEs	first-line therapy
			Placebo	452		matching Placebo		
Cai (25)	2018	metastatic renal cell carcinoma	Sorafenib	110	23	400 mg Bid	OS	first-line therapy
			Sunitinib	74		50 mg Qd		
Rini (26)	2019	metastatic renal cell carcinoma	Atezolizumab+Bevacizumab	454	24	Atezolizumab 1200 mg + Bevacizumab 15 mg/kg intr Q3W	PFS, OS, ORR, grade ≥ 3 AEs	first-line therapy
			Sunitinib	461		50 mg Qd		
McDermott (27)	2018	metastatic renal cell carcinoma	Atezolizumab+Bevacizumab	101	20.7	Atezolizumab 1,200 mg intr + Bevacizumab 15 mg/kg Q3W	PFS, ORR, grade ≥ 3 AEs	first-line therapy
			Sunitinib	101		50 mg Qd		
			Atezolizumab	103		1,200 mg intr Q3W		
Choueiri (12)	2020	advanced renal cell carcinoma	Avelumab+Axitinib	442	18.5	Avelumab 10 mg/kg intr Q2W + Axitinib 5 mg Bid	PFS, OS, ORR	first-line therapy
			Sunitinib	444		50 mg Qd		
Numakura (28)	2021	metastatic renal cell carcinoma	Axitinib	134	20	5 mg Bid	PFS, OS, ORR	first-line therapy
			Sunitinib	274		50 mg Qd		
Hutson (29)	2013	metastatic renal cell carcinoma	Axitinib	192	37	5 mg Bid	PFS, ORR	first-line therapy
			Sorafenib	96		400 mg Bid		
Hutson (30)	2016	metastatic renal cell carcinoma	Axitinib	192	37	5 mg Bid	OS, grade ≥ 3 AEs	first-line therapy
			Sorafenib	96		400 mg Bid		
Rini (31)	2011	advanced renal cell carcinoma	Axitinib	361	30	5 mg Bid	PFS, ORR	first-line therapy
			Sorafenib	362		400 mg Bid		
Rini (32)	2013	advanced renal cell carcinoma	Axitinib	361	30	5 mg Bid	OS, grade ≥ 3 AEs	first-line therapy
			Sorafenib	362		400 mg Bid		
Zhou (33)	2019	metastatic renal cell carcinoma	Anlotinib	90	30.5	12 mg Qd	PFS, OS, ORR, grade ≥ 3 AEs	first-line therapy
			Sunitinib	43		50 mg Qd		
Motzer (8)	2021	advanced renal cell carcinoma	Lenvatinib+Pembrolizumab	355	26.6	Lenvatinib 20 mg Qd + Pembrolizumab 200 mg intr Q3W	PFS, OS, ORR, grade ≥ 3 AEs	first-line therapy
			Sunitinib	357		50 mg Qd		
			Lenvatinib+Everolimus	357		Lenvatinib 18 mg Qd + Everolimus 5 mg Qd		
Motzer (9)	2022	advanced renal cell carcinoma	Nivolumab+Cabozantinib	323	32.9	Nivolumab 240 mg intr Q2W + Cabozantinib 40 mg Qd	PFS, OS, ORR, grade ≥ 3 AEs	first-line therapy
			Sunitinib	328		50 mg Qd		
Albiges (10)	2020	advanced renal cell carcinoma	Nivolumab+lpilimumab	550	55	Nivolumab 3 mg/kg Q3W + Ipilimumab 1 mg/kg Q3W	PFS, OS, ORR, grade ≥ 3 AEs	first-line therapy
			Sunitinib	546		50 mg Qd		
Vano (34)	2022	metastatic renal cell carcinoma	Nivolumab+lpilimumab	41	18	Nivolumab 3 mg/kg Q3W + Ipilimumab 1 mg/kg Q3W	PFS, ORR, grade ≥ 3 AEs	first-line therapy
			Nivolumab	42		Nivolumab 240 mg Q2W		

Qd, quaque die; Bid, bis in die; Q3W, every three weeks; Q2W, every two weeks; PFS, progression free survival; OS, overall survival; ORR, objective response rate; AE, adverse event.

### 3.2 PFS at each time point

**Figure 2:**
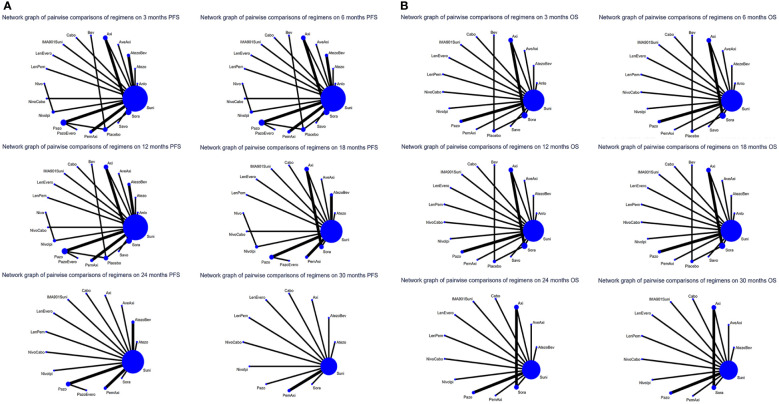
**(A)** Network graphs of pairwise comparison of regimens on each time point of the progression free survival and **(B)** Network graphs of pairwise comparison of regimens on each time point of the overall survival. PFS, progression free survival; OS, overall survival; Suni, Sunitinib; Bev, Bevacizumab; Cabo, Cabozantinib; Pazo, Pazopanib; Savo, Savolitinib; Sora, Sorafenib; Axi, Axitinib; Anlo, Anlotinib; Nivo, Nivolumab; Atezo, Atezolizumab; PemAxi, Pembrolizumab plus Axitinib; AtezoBev, Atezolizumab plus Bevacizumab; AveAxi, Avelumab plus Axitinib; LenPem, Lenvatinib plus Pembrolizumab; NivoCabo, Nivolumab plus Cabozantinib; NivoIpi, Nivolumab plus lpilimumab; LenEvero, Lenvatinib plus Everolimus; PazoEvero, Pazopanib plus Everolimus; IMA901Suni, IMA901 plus Sunitinib.

For PFS, 22 out of 26 articles reported related outcomes. At the 6-time points of 3, 6, 12, 18, 24 and 30 month, there were sufficient data for the NMA of PFS. **Figure 2A** shows the network graphs of pairwise comparison of regimens on each time point of the PFS. The most used agent was Suni, the most comparisons were between Pazo and Suni and between Axi and Sora. Detailed results of direct and indirect comparisons of 19 interventions at each time point are shown in the **Supplementary Table 2A-F** and **Supplementary Figure 3**. Due to the wide use of Suni in the clinic and as the first-line standard treatment, we regard Suni as the primary reference and the top SUCRA-ranked intervention as the secondary reference.

At 3^rd^ month, compared with Suni, LenPem (OR=2.80,95% Cl: 1.69 to 4.65), Axi (OR=2.55, 95%CI: 1.30 to 5.00), NivoCabo (OR=2.46, 95% Cl: 1.54 to 3.93), LenEvero (OR=1.97, 95%CI: 1.24 to 3.12), PemAxi (OR=1.72, 95%CI: 1.20 to 2.45) and AveAxi (OR=1.64, 95%CI: 1.20 to 2.24) significantly increased PFS rates. Compared with the top SUCRA-ranked intervention LenPem, Axi ranked second.

In comparison with Suni at 6^th^ month, LenPem (OR=4.47,95% Cl: 3.10 to 6.44), Axi (OR=2.32, 95%CI: 1.37 to 3.93), NivoCabo (OR=2.52, 95% Cl: 1.78 to 3.58), LenEvero (OR=1.71, 95%CI: 1.26 to 2.33), PemAxi (OR=1.52, 95%CI: 1.14 to 2.02) and AveAxi (OR=1.62, 95%CI: 1.23 to 2.13) significantly improved PFS. As compared with the top SUCRA-ranked intervention LenPem, NivoCabo ranked second.

At 12^th^ month, the PFS for LenPem (OR=3.88,95% Cl: 2.84 to 5.30), Axi (OR=3.18, 95%CI: 1.86 to 5.42), NivoCabo (OR=2.44, 95% Cl: 1.78 to 3.35), LenEvero (OR=1.95, 95%CI: 1.45 to 2.63), PemAxi (OR=1.72, 95%CI: 1.33 to 2.23) and AveAxi (OR=1.71, 95%CI: 1.31 to 2.24) and AtezoBev (OR=1.38, 95%CI: 1.09 to 1.74) were significantly higher compared to that of Suni. Among them, the highest SUCRA ranking is LenPem followed by Axi.

At 18^th^ month, the PFS was significantly higher for LenPem (OR=2.95, 95%Cl: 1.94 to 4.49), Axi (OR=3.00, 95%CI: 1.62 to 5.56), NivoCabo (OR=2.49, 95% Cl: 1.61 to 3.85), LenEvero (OR=1.62, 95%CI: 1.07 to 2.46), PemAxi (OR=1.76, 95%CI: 1.22 to 2.55), AveAxi (OR=1.91, 95%CI: 1.28 to 2.84), AtezoBev (OR=1.52, 95%CI: 1.05 to 2.22) and Sora (OR=2.38, 95%CI: 1.13 to 5.02) compared to Suni. Axi is the highest SUCRA ranking, LenPem was remarkably inferior to Axi.

At 24^th^ month, the PFS increased significantly when LenPem (OR=3.46, 95%Cl: 2.50 to 4.79), Axi (OR=2.81, 95%CI: 1.57 to 5.04), NivoCabo (OR=2.48, 95% Cl: 1.75 to 3.51), LenEvero (OR=1.61, 95%CI: 1.15 to 2.25), PemAxi (OR=1.70, 95%CI: 1.29 to 2.24) and AveAxi (OR=1.91, 95%CI: 1.44 to 2.52) were compared with Suni. LenPem has the highest SUCRA ranking, while Axi is remarkably inferior to LenPem.

At 30^th^ month, the PFS was significantly higher for LenPem (OR=3.26, 95%Cl: 2.26 to 4.71), Axi (OR=2.28, 95%CI: 1.22 to 4.26), NivoCabo (OR=3.10, 95%Cl: 2.08 to 4.62), LenEvero (OR=2.14, 95%CI: 1.47 to 3.13), PemAxi (OR=1.80, 95%CI: 1.34 to 2.42) and Nivolpi (OR=1.51, 95%CI: 1.17 to 1.96) compared to Suni. LenPem is ranked highest SUCRA among them, followed by NivoCabo.

At 36^th^ month, the PFS was significantly higher for LenPem (OR=3.26, 95%Cl: 2.26 to 4.71), Axi (OR=2.20, 95%CI: 1.09 to 4.44), NivoCabo (OR=3.55, 95%Cl: 2.22 to 5.67), LenEvero (OR=2.14, 95%CI: 1.47 to 3.13) and Nivolpi (OR=1.58, 95%CI: 1.21 to 2.06) compared to Suni. According to their SUCRA rankings, NivoCabo is the highest followed by LenPem.

In PFS, compared with Suni, the intervention measures with significant effect from 3 to 36 months were LenPem, Axi, NivoCabo and LenEvero in order from high to low. We summarize the details of the interventions with significant results compared with Suni in **Table 2**.

**Table 2:** Progression free survival for each time point for interventions that were significant compared to Sunitinib (shown as odds ratio and 95% confidence intervals).

Time point	Control group	LenPem	Axi	NivoCabo	LenEvero	PemAxi	AveAxi	AtezoBev	Nivolpi	Sora
3 month	Suni	2.80 (1.69,4.65)	2.55 (1.30,5.00)	2.46 (1.54,3.93)	1.97 (1.24,3.12)	1.72 (1.20,2.45)	1.64 (1.20,2.24)	×	×	×
	Placebo	√	√	√	√	√	√	√	√	×
6 month	Suni	4.47 (3.10,6.44)	2.32 (1.37,3.93)	2.52 (1.78,3.58)	1.71 (1.26,2.33)	1.52 (1.14,2.02)	1.62 (1.23,2.13)	×	×	×
	Placebo	√	√	√	√	√	√	√	×	√
12 month	Suni	3.88 (2.84,5.30)	3.18 (1.86,5.42)	2.44 (1.78,3.35)	1.95 (1.45,2.63)	1.72 (1.33,2.23)	1.71 (1.31,2.24)	1.38 (1.09,1.74)	×	×
	Placebo	√	√	√	√	√	√	√	×	√
18 month	Suni	2.95 (1.94,4.49)	3.00 (1.62,5.56)	2.49 (1.61,3.85)	1.62 (1.07,2.46)	1.76 (1.22,2.55)	1.91 (1.28,2.84)	1.52 (1.05,2.22)	×	2.38 (1.13,5.02)
	Placebo	-	-	-	-	-	-	-	-	-
24 month	Suni	3.46 (2.50,4.79)	2.81 (1.57,5.04)	2.48 (1.75,3.51)	1.61 (1.15,2.25)	1.70 (1.29,2.24)	1.91 (1.44,2.52)	×	×	×
	Placebo	-	-	-	-	-	-	-	-	-
30 month	Suni	3.26 (2.26,4.71)	2.28 (1.22,4.26)	3.10 (2.08,4.62)	2.14 (1.47,3.13)	1.80 (1.34,2.42)	-	×	1.51 (1.17,1.96)	×
	Placebo	-	-	-	-	-	-	-	-	-
36 month	Suni	3.26 (2.26,4.71)	2.20 (1.09,4.44)	3.55 (2.22,5.67)	2.14 (1.47,3.13)	-	-	-	1.58 (1.21,2.06)	×
	Placebo	-	-	-	-	-	-	-	-	-

Suni, Sunitinib; Sora, Sorafenib; Axi, Axitinib; PemAxi, Pembrolizumab plus Axitinib; AtezoBev, Atezolizumab plus Bevacizumab, AveAxi, Avelumab plus Axitinib; LenPem, Lenvatinib plus Pembrolizumab; NivoCabo, Nivolumab plus Cabozantinib; NivoIpi, Nivolumab plus lpilimumab; LenEvero, Lenvatinib plus Everolimus.

√: the treatment on the top is significant compared to the control group on the left; ×: the treatment on the top is not significant compared to the Control group on the left.

### 3.3 OS at each time point

18 out of 26 articles reported outcomes related to OS. In this study, adequate data were available at 3, 6, 12, 24 and 30 month to conduct NMA. An analysis of pairwise comparison of regimens on every OS time point is shown in **Figure 2B**. As for agents, Suni was most commonly used, with Axi and Sora being most commonly compared. A detailed comparison of 17 interventions at each time point is presented in **Supplementary Tables 3A-F** and **Supplementary Figure 4**.

At 3^rd^ month, two interventions were significantly compared with Suni, but were not significantly compared with placebo. Therefore, at 3^rd^ month, there were no interventions with significant results.

At 6^th^ month, in comparison with Suni, PemAxi (OR=2.47, 95%CI: 1.45 to 4.19), NivoCabo (OR=2.07, 95%Cl: 1.22 to 3.52), Axi (OR=2.13, 95%CI: 1.07 to 4.23) and LenPem (OR=3.05, 95%Cl: 1.46 to 6.36) were more effective, in which LenPem scored highest and PemAxi was second.

At 12^th^ month, PemAxi (OR=2.37, 95%CI: 1.61 to 3.50), NivoCabo (OR=1.85, 95%Cl: 1.24 to 2.75), Nivolpi (OR=1.48, 95%CI: 1.10 to 2.00) and LenPem (OR=2.64, 95%Cl: 1.68 to 4.14) significantly increased OS rate compared to Suni. According to their SUCRA rankings, LenPem ranked first, and PemAxi ranked second.

At 18^th^ month, there was a significant increase in OS rate with PemAxi (OR=1.67, 95%CI: 1.22 to 2.30), NivoCabo (OR=1.69, 95%Cl: 1.18 to 2.40), Nivolpi (OR=1.57, 95%CI: 1.20 to 2.05), LenPem (OR=2.33, 95%Cl: 1.58 to 3.44) and AveAxi (OR=1.41, 95%CI: 1.03 to 1.92) compared with Suni. Rankings based on SUCRA, LenPem was ranked first, followed by NivoCabo.

At 24^th^ month, OS rate was significantly increased by PemAxi (OR=1.52, 95%CI: 1.14 to 2.04), NivoCabo (OR=1.57, 95%Cl: 1.13 to 2.17), Nivolpi (OR=1.58, 95%CI: 1.23 to 2.03) and LenPem (OR=1.66, 95%Cl: 1.18 to 2.34) in comparison with Suni. In accordance with their SUCRA rankings, LenPem showed the best results, while Nivolpi was ranked second.

At 30^th^ month, OS rates were significantly higher with PemAxi (OR=1.41, 95%CI: 1.07 to 1.86), NivoCabo (OR=1.64, 95%Cl: 1.19 to 2.25), Nivolpi (OR=1.43, 95%CI: 1.12 to 1.82) and AveAxi (OR=1.71, 95%Cl: 1.31 to 2.24) than with Suni. As ranked by SUCRA, AveAxi performed best, followed by NivoCabo.

Among the treatments tested at 36^th^ month, the OS rate of PemAxi (OR=1.84, 95%CI: 1.40 to 2.42), NivoCabo (OR=1.41, 95%Cl: 1.03 to 1.92) and Nivolpi (OR=1.38, 95%CI: 1.09 to 1.76) was significantly higher than that of Suni. Rankings according to SUCRA, PemAxi had the best outcome, followed by NivoCabo.

In OS, compared with Suni, PemAxi and NivoCabo were significant from 6 to 36 months; Nivolpi significant from 12 to 36 months; LenPem significant from 6 to 24 months. We summarize the details of the interventions with significant results compared with Suni in **Table 3**.

**Table 3:** Overall survival for each time point for interventions that were significant compared to Sunitinib (shown as odds ratio and 95% confidence intervals).

Time point	Control group	IMA901Suni	PemAxi	NivoCabo	Nivolpi	Axi	LenPem	AveAxi
3 month	Suni	3.57 (1.08,11.84)	2.73 (1.19,6.23)	×	×	×	×	×
	Placebo	×	×	×	×	×	×	×
6 month	Suni	×	2.47 (1.45,4.19)	2.07 (1.22,3.52)	×	2.13 (1.07,4.23)	3.05 (1.46,6.36)	×
	Placebo	×	√	√	√	√	√	√
12 month	Suni	×	2.37 (1.61,3.50)	1.85 (1.24,2.75)	1.48 (1.10,2.00)	×	2.64 (1.68,4.14)	×
	Placebo	×	√	√	√	√	√	√
18 month	Suni	×	1.67 (1.22,2.30)	1.69 (1.18,2.40)	1.57 (1.20,2.05)	×	2.33 (1.58,3.44)	1.41 (1.03,1.92)
	Placebo	√	√	√	√	√	√	√
24 month	Suni	×	1.52 (1.14,2.04)	1.57 (1.13,2.17)	1.58 (1.23,2.03)	×	1.66 (1.18,2.34)	×
	Placebo	-	-	-	-	-	-	-
30 month	Suni	×	1.41 (1.07,1.86)	1.64 (1.19,2.25)	1.43 (1.12,1.82)	×	×	1.71 (1.31,2.24)
	Placebo	-	-	-	-	-	-	-
36 month	Suni	×	1.84 (1.40,2.42)	1.41(1.03,1.92)	1.38(1.09,1.76)	×	×	×
	Placebo	-	-	-	-	-	-	-

Suni, Sunitinib; IMA901Suni, IMA901 plus Sunitinib; Axi, Axitinib; PemAxi, Pembrolizumab plus Axitinib; AveAxi, Avelumab plus Axitinib; LenPem, Lenvatinib plus Pembrolizumab; NivoCabo, Nivolumab plus Cabozantinib; NivoIpi, Nivolumab plus lpilimumab.

√, the treatment on the top is significant compared to the control group on the left; ×, the treatment on the top is not significant compared to the Control group on the left.

### 3.4 Survival Analysis of PFS, OS, ORR and AEs (grade ≥ 3)

**Figure 3:**
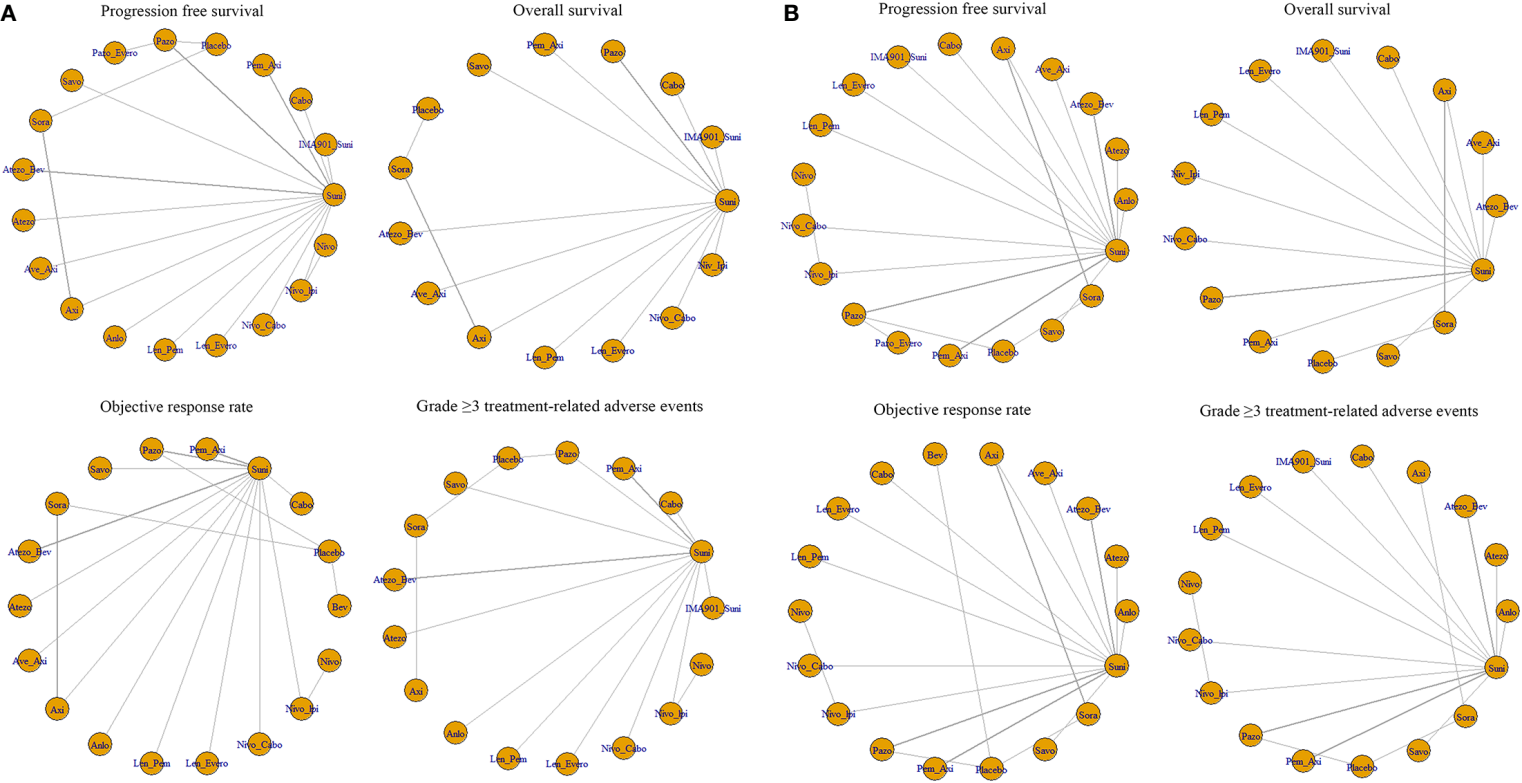
**(A)** Network meta-analysis plots for outcomes of interest in intent-to-treat population and **(B)** Network meta-regression analysis plots for outcomes of interest in intent-to-treat population. Suni, Sunitinib; Bev, Bevacizumab; Cabo, Cabozantinib; Pazo, Pazopanib; Savo, Savolitinib; Sora, Sorafenib; Axi, Axitinib; Anlo, Anlotinib; Nivo, Nivolumab; Atezo, Atezolizumab; Pem_Axi, Pembrolizumab plus Axitinib; Atezo_Bev, Atezolizumab plus Bevacizumab; Ave_Axi, Avelumab plus Axitinib; Len_Pem, Lenvatinib plus Pembrolizumab; Nivo_Cabo, Nivolumab plus Cabozantinib; Nivo_Ipi, Nivolumab plus lpilimumab; Len_Evero, Lenvatinib plus Everolimus; Pazo_Evero, Pazopanib plus Everolimus; IMA901_Suni, IMA901 plus Sunitinib.

Twenty-two of the 26 articles reported outcomes related to the HRs of PFS. We compared the 19 interventions included directly and indirectly. The network graph is shown in **Figure 3A**. The intervention measures with significant difference compared with Suni are LenPem (HR=2.56, 95%Crl: 1.55 to 4.24), Cabo (HR=2.08, 95%Crl: 1.12 to 3.87), AveAxi (HR=1.82, 95%Crl: 1.10 to 3.02), NivoCabo (HR=1.79, 95%Crl: 1.09 to 2.94) and PemAxi (HR=1.46, 95%Crl: 1.01 to 2.19). Compared with the top SUCRA-ranked intervention LenPem, Cabo ranked second. Detailed results are shown in **Supplementary Table 4A**.

The results related to OS were reported in 18 of 26 articles. A direct and indirect comparison was made between the included 15 interventions. The network graph is shown in **Figure 3A**. The interventions with significant differences from Suni are LenPem (HR=2.27, 95%Crl 1.28 to 4.03), PemAxi (HR=2.08, 95%Crl 1.07 to 4.03), and AveAxi (HR=1.93, 95%Crl 1.04 to 3.56). Among them, the highest SUCRA ranking is LenPem, followed by PemAxi. Detailed results are shown in **Supplementary Table 4B**.

Results related to ORR were reported in 20 out of 26 articles. The 18 interventions were compared directly and indirectly. The network graph is shown in **Figure 3A**. The interventions that showed significant difference compared with Suni are LenPem (HR=4.36, 95%Crl 2.28 to 8.18), Cabo (HR=4.01, 95%Crl 1.44 to 11.40), NivoCabo (HR=3.19, 95%Crl 1.68 to 6.16), AveAxi (HR=2.98, 95%Crl 1.57 to 5.54), PemAxi (HR=2.28, 95%Crl 1.35 to 3.80) and LenEvero (HR=2.04, 95%Crl 1.07 to 3.82). In order of SUCRA ranking, LenPem has the highest ranking, followed by Cabo. Detailed results are shown in **Supplementary Table 4C**.

Regarding AEs (grade ≥ 3), indirect and direct comparisons were conducted between 16 interventions. The network graph is shown in **Figure 3A**. Nivolumab, Sora, Atezolizumab, Anlotinib, Savo, Nivolpi and Atezolizumab plus bevacizumab are less toxic than Suni. Cabo, PemAxi, NivoCabo, Axi, Pazo had no significant difference compared with Suni. IMA901 plus Suni, LenPem and LenEevero are more toxic than Suni. Detailed results are shown in **Supplementary Table 4D**.

For all outcomes, it was revealed in Brooks-GelmanRubin diagnostic that the inferential iterations for each MCMC were stable and reproducible. We used history feature to confirm the convergence of the model in all outcomes as well. Detailed results are presented in **Supplementary Figures 5A-D** and **Supplementary Figures 6A-D**.

### 3.5 Heterogeneity and network meta-regression analysis

In order to better explain the heterogeneity, we performed sensitivity analysis on the four outcomes. Meta-regression analysis was performed on PFS, OS, ORR and AEs (grade ≥ 3) with the median follow-up time as the covariate. The network graphs are shown in **Figure 3B**. Detailed results are shown in **Supplementary Table 5A-D**.

For PFS, after meta-regression analysis, PemAxi, NivoCabo are not significantly compared with Suni, LenPem, Cabo and AveAxi are consistently better than Suni. For OS, PemAxi and LenPem are still better than standard Suni. For ORR, in comparison to Suni, Cabo, LenPem, PemAxi, NivoCabo and LenEvero consistently provide better results. For AEs (grade ≥ 3), after meta-regression analysis, the serious adverse events of LenPem and LenEvero were not different from that of Suni. The detailed results before and after meta-regression are shown in the **Table 4**.

**Table 4:** Outcomes of interest in intent-to-treat population compared to Sunitinib before and after meta-regression (shown as hazard ratio and 95% credible intervals).

Outcomes	Methods	Cabo	PemAxi	AveAxi	LenPem	NivoCabo	LenEvero
PFS	meta-analysis	2.08(1.12,3.87)	1.46(1.01,2.19)	1.82(1.10,3.02)	2.56(1.55,4.24)	1.79(1.09,2.94)	×
	meta-regression analysis	2.30(1.10,4.60)	×	2.10(1.02,4.40)	2.50(1.50,4.30)	×	×
OS	meta-analysis	×	2.08(1.07,4.03)	1.93(1.04,3.56)	2.27(1.28,4.03)	×	×
	meta-regression analysis	×	2.00(1.81,4.30)	×	1.90(1.30,4.50)	×	×
ORR	meta-analysis	4.01(1.44,11.40)	2.28(1.35,3.80)	2.98(1.57,5.54)	4.36(2.28,8.18)	3.19(1.68,6.16)	2.04(1.07,3.82)
	meta-regression analysis	3.80(1.10,12.00)	2.20(1.04,4.90)	×	4.30(2.10,8.90)	3.10(1.02,9.90)	2.00(1.08,4.10)
AE	meta-analysis	×	×	-	2.07 (1.46,2.93)	×	2.20 (1.55,3.14)
	meta-regression analysis	×	×	-	×	×	×

PemAxi, Pembrolizumab plus Axitinib; AveAxi, Avelumab plus Axitinib; LenPem, Lenvatinib plus Pembrolizumab; NivoCabo, Nivolumab plus Cabozantinib; LenEvero, Lenvatinib plus Everolimus; Cabo, Cabozantinib.

×: the treatment on the top is not significant compared to Sunitinib.

For meta-regression, as shown by Brooks-GelmanRubin diagnostic, inferential iterations were reproducible and stable for each MCMC. Additionally, we used the history feature to confirm the model’s convergence in all outcomes. Detailed results are presented in **Supplementary Figures 7A-D** and **Figures 8A-D**.

## 4 Discussion

### 4.1 Principal findings

This is the first Bayesian NMA investigating the pairwise effect of regimens on OS and PFS at each time node; meanwhile, the prominent innovativeness is the implementation of network meta-regression analysis adjusting confounding factor, which is the Qomolangma in NMA. There are the following findings.

Regarding PFS, compared to Suni, the interventions with significant effects were LenPem, Axi, NivoCabo and LenEvero from high to low from 3 to 36 months. PemAxi and AveAxi also showed good results compared to Suni, but due to the lack of data, it was not possible to tell whether the significance persisted until month 36. Based on Bayesian NMA of HRs of PFS and Bayesian network meta-regression analysis with median follow-up time as a covariate, the comparisons with Suni were significant in descending order of LenPem, Cabo and AveAxi. In summary, LenPem is the first choice for improving PFS.

Regarding OS, from 6 to 36 months, PemAxi and NivoCabo were significantly superior to Suni. Bayesian NMA of HRs of OS and Bayesian network regression analysis with median follow-up time as covariate showed that LenPem and PemAxi were significantly different from Suni. Considering LenPem significant only from 6 to 24 months, PemAxi is the first choice for improving OS.

Regarding ORR, in comparison to standard chemotherapy, Cabo, LenPem, PemAxi, NivoCabo and LenEvero consistently provide better results. Notably, LenEvero needs to be excluded because according to the Meta-regression analysis, LenEvero is inferior to D in both primary endpoints PFS and OS although the ORR results are significant.

Regarding AEs (grade ≥ 3), after Bayesian network meta-regression analysis with median follow-up time as a covariate, Atezo and Savo were significantly less toxic than Suni, and the rest were not significant with Suni. However, both Atezo and Savo were inferior to Suni in primary endpoints PFS and OS, so they were not considered as first-line therapeutic agents for mRCC.

These results demonstrate that the combination of ICI-TKI has significant OS, PFS and ORR benefits in patients with mRCC. Interestingly, clinical studies show the separating survival benefit of ICI-TKI much earlier than ICI-ICI in first-line treatment of mRCC (9, 35). As well, some investigations showed that the OS of ICI-TKI combination treatment effect is more favorable than dual combination immunotherapy (10, 11). Moreover, the comparison of efficacy results and tumors with sarcomatoid differentiation in clinical trials concluded that the ORR and PFS of PemAxi were superior to those of Nivolpi (36, 37). Therefore, ICI-TKI combination therapy is also the preferred therapy for aggressive, rapidly progressive renal cancer.

RCC is a highly vascularized tumor, and the expression level of VEGF-A is significantly higher in RCC patients than in patients with other types of cancer (38). In addition, TKI can increase immune infiltration directly or indirectly while improving vascularity (39, 40). Studies have shown that both Cabo and Len have modulating and immune-promoting properties (41, 42). Thus, the combination of TKI and ICI has a synergistic anti-tumor effect. Treatment-related toxicity should also be considered when TKI and ICI are used in combination. ICI produces immune-related adverse events, while TKI is chronically toxic. The toxicity of the combination, although greater than that of monotherapy such as Atezo, was not significantly compared to standard treatment. Its toxicity is within the acceptable range, only the superposition of dual adverse effects will increase the difficulty of clinical management.

In our research, although both LenPem and PemAxi showed significant advantages in terms of PFS and ORR, the toxicity of LenPem cannot be ignored, and in this clinical trial, the dose of Lenvatinib was consistently reduced by constant reductions to reduce adverse events that could discontinue treatment. In terms of OS, the remarkable performance of PemAxi lasts up to 36 months, while LenPem lasts only up to 24 months. In addition, the toxicity of PemAxi is lower and the quality of life of patients is higher. In summary, our study shows that for mRCC patients PemAxi can have better survival outcomes, lower toxicity, and higher quality of life. Therefore PemAxi appears to be the superior first-line TKI-ICI combination.

### 4.2 Previous network meta-analyses

Treatments for mRCC in the first instance has been changing rapidly in recent years, and the earlier NMAs did not incorporate the multiple ICI-TKI interventions recommended by the mRCC first-line treatment guidelines in recent years (43, 44). In 2019, Wang et al (45) published a NMA that focused only on the analysis of ICI and included many interventions that were completely withdrawn from the clinic due to high toxicity, such as IFN-α and IL-2. Manz et al (46) published a NMA in 2020, Focusing solely on TKI. Other studies have focused only on immune-based interventions (47, 48). In two recent studies, they included only a few treatment nodes and used a frequentist NMA rather than a Bayesian framework (49, 50).

### 4.3 Strengths and limitations

We evaluated 19 first-line interventions using 26 high-quality studies that were screened. First, we used a Bayesian framework that is more flexible relative to the frequentist, describing pairwise comparisons in terms of probabilistically distributed random variables (51). Second, for the analysis of PFS and OS, it lasted until 36 months. In addition, based on PFS, OS, ORR and AEs (grade ≥ 3), we performed a sensitivity analysis. We performed a network meta-regression analysis with median follow-up time as a covariate. Third, due to the inclusion of a sufficient number of studies, we performed a paired meta-analysis. Closed loops existed in the network, so heterogeneity was also evaluated and the results showed good agreement between the trials included in the study. Fourth, the stability and replicability of each MCMC chain iteration was demonstrated using Brooks-GelmanRubin diagnostics, as well as the convergence of the model was estimated.

A number of limitations are associated with this NMA. First, we have compared ICI and TKI combinations directly or indirectly; however, this approach cannot fully replace a head-to-head comparison. Therefore direct comparative clinical trials are still indispensable. Second, the quality of the trials included in this analysis may have been affected by several types of bias, which could have some impact on the validity of the overall outcomes. Third, the study population included patients with clear cell histology, so the final results are not applicable to patients with non-clear cell histology. Fourth, only trials with standard dosing regimens were included in this study, and the doses and schedules administered in actual clinical settings may differ from those of the included studies; consequently, efficacy and tolerability may be affected to some extent. Some investigations have demonstrated that modifications to the dose and schedule pattern of Suni administration may improve its efficacy and enhance tolerability (52, 53). Fifth, there was a large variation in median follow-up time across studies, and although this influence was corrected using meta-regression, the results need to be further investigated in the clinic. In addition, another part of confounding factors (e.g., PD-L1 status, number of focal metastases, patient risk class, etc.) had missing data in some trials, and we could not correct for these factors using meta-regression; therefore, the results of this Bayesian NMA need to be treated with caution.

### 4.4 Future research

We hope that future clinical studies will be more precise and focus more on the outcomes of ORR and AEs at each time point. According to the studies, the median follow-up period ranged from 6.4 to 55 months, and the wide variation in follow-up time can have an impact on outcome indicators. Although we corrected for this with meta-regression using time as a covariate, the results this result cannot be used as a proxy for accurate clinical studies. The findings would be more convincing if the ORR or AEs of different interventions were compared at the same time points.

## 5 Conclusion

Considering the lower toxicity and the higher quality of life, PemAxi should be recommended as the optimal therapy in treating mRCC. Certainly, it is necessary to conduct more head-to-head comparisons in order to confirm these findings.

## Data availability statement

The original contributions presented in the study are included in the article/**Supplementary Material**. Further inquiries can be directed to the corresponding author.

## Author contributions

SQ: Conceptualization, Methodology, Formal analysis, Writing - Original Draft. ZX: Investigation, Data Curation. XC: Methodology, Writing - Review & Editing. SW: Formal analysis, Writing - Review & Editing. HL: Formal analysis, Writing - Review & Editing. JL: Visualization, Writing - Original Draft. XG: Visualization, Writing - Original Draft. JY: Visualization, Writing - Original Draft. CL: Supervision, Conceptualization. YW: Supervision, Conceptualization. HW: Formal analysis, Supervision. All authors contributed to the article and approved the submitted version.
