# Supplementary material for: Comparing efficacy and safety of first-line treatment of metastatic renal cell carcinoma: A Bayesian network meta-regression analysis
Source: Front Oncol. 2023 Feb 22;13:1072634. doi: 10.3389/fonc.2023.1072634 (PMC9992527; doi:10.3389/fonc.2023.1072634)
Supplement: Supplementary file 1 [file DataSheet_1.zip › Appendix.docx]

Supplementary Figure 1. Summary of quality assessments using Cochrane Risk of Bias Tool 2.

Supplementary Figure 2A (Continued)

Supplementary Figure 2A. Network funnel plot of pairwise comparisons of each time point of the progression free survival.

Supplementary Figure 2B (Continued)

Supplementary Figure 2B. Network funnel plot of pairwise comparisons of each time point of the overall survival.

Supplementary Table 2A. Matrix of pairwise comparisons of regimens on 3 month progression free survival (shown as odds ratio and 95% confidence intervals).

| LenPem | 0.91 (0.39,2.12) | 0.88 (0.44,1.75) | 1.15 (0.21,6.33) | 0.70 (0.35,1.40) | 0.61 (0.33,1.14) | 0.59 (0.32,1.06) | 0.44 (0.15,1.30) | 0.42 (0.12,1.50) | 0.42 (0.18,0.97) |
| --- | --- | --- | --- | --- | --- | --- | --- | --- | --- |
| 1.10 (0.47,2.56) | Axi | 0.97 (0.42,2.20) | 1.26 (0.22,7.37) | 0.77 (0.34,1.75) | 0.67 (0.31,1.45) | 0.65 (0.31,1.36) | 0.49 (0.15,1.57) | 0.47 (0.12,1.78) | 0.46 (0.18,1.19) |
| 1.14 (0.57,2.27) | 1.04 (0.46,2.36) | NivoCab | 1.31 (0.24,7.13) | 0.80 (0.42,1.55) | 0.70 (0.39,1.26) | 0.67 (0.38,1.17) | 0.50 (0.17,1.46) | 0.48 (0.14,1.68) | 0.48 (0.21,1.08) |
| 0.87 (0.16,4.82) | 0.79 (0.14,4.64) | 0.77 (0.14,4.19) | Cabo | 0.61 (0.11,3.35) | 0.54 (0.10,2.84) | 0.51 (0.10,2.70) | 0.39 (0.06,2.56) | 0.37 (0.05,2.73) | 0.37 (0.06,2.14) |
| 1.42 (0.72,2.82) | 1.29 (0.57,2.92) | 1.25 (0.65,2.41) | 1.63 (0.30,8.88) | LenEvero | 0.87 (0.49,1.56) | 0.83 (0.48,1.45) | 0.63 (0.22,1.81) | 0.60 (0.17,2.09) | 0.60 (0.27,1.34) |
| 1.63 (0.88,3.03) | 1.48 (0.69,3.18) | 1.43 (0.79,2.58) | 1.87 (0.35,9.93) | 1.15 (0.64,2.05) | PemAxi | 0.96 (0.60,1.53) | 0.72 (0.26,2.00) | 0.69 (0.21,2.32) | 0.69 (0.33,1.45) |
| 1.71 (0.94,3.09) | 1.55 (0.74,3.26) | 1.50 (0.85,2.63) | 1.95 (0.37,10.29) | 1.20 (0.69,2.09) | 1.05 (0.65,1.67) | AveAxi | 0.75 (0.28,2.06) | 0.72 (0.22,2.39) | 0.72 (0.35,1.49) |
| 2.26 (0.77,6.68) | 2.06 (0.64,6.63) | 1.99 (0.68,5.76) | 2.59 (0.39,17.18) | 1.59 (0.55,4.60) | 1.39 (0.50,3.85) | 1.33 (0.49,3.62) | PazoEvero | 0.96 (0.21,4.30) | 0.96 (0.30,3.05) |
| 2.36 (0.67,8.38) | 2.15 (0.56,8.22) | 2.08 (0.59,7.25) | 2.71 (0.37,20.06) | 1.66 (0.48,5.79) | 1.45 (0.43,4.87) | 1.39 (0.42,4.60) | 1.05 (0.23,4.69) | Anlo | 1.00 (0.26,3.79) |
| 2.37 (1.03,5.43) | 2.15 (0.84,5.52) | 2.08 (0.93,4.66) | 2.71 (0.47,15.75) | 1.67 (0.75,3.71) | 1.45 (0.69,3.06) | 1.39 (0.67,2.87) | 1.05 (0.33,3.34) | 1.00 (0.26,3.79) | IMA901Suni |
| 2.45 (1.38,4.35) | 2.22 (1.07,4.60) | 2.15 (1.25,3.69) | 2.80 (0.54,14.66) | 1.72 (1.01,2.93) | 1.50 (0.96,2.34) | 1.43 (0.95,2.16) | 1.08 (0.43,2.70) | 1.03 (0.31,3.40) | 1.03 (0.51,2.10) |
| 2.53 (1.41,4.53) | 2.30 (1.11,4.79) | 2.22 (1.28,3.85) | 2.90 (0.55,15.21) | 1.78 (1.04,3.06) | 1.55 (0.98,2.45) | 1.48 (0.97,2.26) | 1.12 (0.41,3.03) | 1.07 (0.32,3.53) | 1.07 (0.52,2.19) |
| 2.80 (1.69,4.65) | 2.55 (1.30,5.00) | 2.46 (1.54,3.93) | 3.21 (0.63,16.41) | 1.97 (1.24,3.12) | 1.72 (1.20,2.45) | 1.64 (1.20,2.24) | 1.24 (0.48,3.22) | 0.05 (0.01,0.47) | 0.85 (0.44,1.63) |
| 3.31 (1.83,5.97) | 3.01 (1.44,6.30) | 2.91 (1.66,5.07) | 3.79 (0.72,19.94) | 2.33 (1.34,4.03) | 2.03 (1.27,3.23) | 1.94 (1.26,2.99) | 1.46 (0.54,3.98) | 1.40 (0.42,4.64) | 1.40 (0.68,2.88) |
| 4.00 (1.16,13.74) | 3.64 (0.98,13.50) | 3.51 (1.04,11.87) | 4.58 (0.63,33.27) | 2.81 (0.84,9.48) | 2.45 (0.75,7.98) | 2.35 (0.73,7.53) | 1.77 (0.40,7.73) | 4.11 (1.16,14.57) | 1.69 (0.46,6.22) |
| 4.39 (1.38,14.02) | 3.99 (1.15,13.84) | 3.86 (1.23,12.11) | 5.03 (0.73,34.93) | 3.09 (0.99,9.67) | 2.69 (0.89,8.11) | 2.58 (0.87,7.65) | 1.94 (0.50,7.58) | 1.86 (0.39,8.84) | 1.86 (0.54,6.37) |
| 4.53 (2.06,9.94) | 4.12 (1.67,10.17) | 3.97 (1.85,8.52) | 5.19 (0.91,29.54) | 3.19 (1.50,6.79) | 2.78 (1.38,5.58) | 2.66 (1.35,5.22) | 2.00 (0.65,6.19) | 1.92 (0.52,7.07) | 1.91 (0.79,4.66) |
| 7.70 (2.54,23.33) | 7.00 (2.12,23.12) | 6.75 (2.27,20.13) | 8.82 (1.31,59.36) | 5.41 (1.82,16.07) | 4.72 (1.65,13.46) | 4.51 (1.61,12.68) | 3.40 (0.86,13.43) | 3.25 (0.71,14.91) | 3.25 (0.99,10.63) |
| 9.71 (4.73,19.95) | 8.83 (3.78,20.59) | 8.52 (4.26,17.05) | 11.13 (2.01,61.53) | 6.83 (3.44,13.58) | 5.95 (3.20,11.09) | 5.69 (3.13,10.35) | 4.29 (1.56,11.83) | 9.72 (1.15,82.00) | 4.10 (1.79,9.43) |
| 29.81 (3.60,246.69) | 27.10 (3.13,234.93) | 26.16 (3.19,214.60) | 34.16 (2.48,469.89) | 20.97 (2.56,171.66) | 18.28 (2.28,146.60) | 17.48 (2.20,139.19) | 13.18 (1.37,126.66) | 1.69 (0.34,8.51) | 12.60 (1.46,108.58) |

Supplementary Table 2A (continued)

| 0.41 (0.23,0.73) | 0.40 (0.22,0.71) | 0.36 (0.21,0.59) | 0.30 (0.17,0.55) | 0.25 (0.07,0.86) | 0.23 (0.07,0.73) | 0.22 (0.10,0.48) | 0.13 (0.04,0.39) | 0.10 (0.05,0.21) | 0.03 (0.00,0.28) |
| --- | --- | --- | --- | --- | --- | --- | --- | --- | --- |
| 0.45 (0.22,0.93) | 0.43 (0.21,0.90) | 0.39 (0.20,0.77) | 0.33 (0.16,0.70) | 0.28 (0.07,1.02) | 0.25 (0.07,0.87) | 0.24 (0.10,0.60) | 0.14 (0.04,0.47) | 0.11 (0.05,0.26) | 0.04 (0.00,0.32) |
| 0.47 (0.27,0.80) | 0.45 (0.26,0.78) | 0.41 (0.25,0.65) | 0.34 (0.20,0.60) | 0.28 (0.08,0.96) | 0.26 (0.08,0.81) | 0.25 (0.12,0.54) | 0.15 (0.05,0.44) | 0.12 (0.06,0.23) | 0.04 (0.00,0.31) |
| 0.36 (0.07,1.87) | 0.34 (0.07,1.81) | 0.31 (0.06,1.59) | 0.26 (0.05,1.39) | 0.22 (0.03,1.58) | 0.20 (0.03,1.38) | 0.19 (0.03,1.10) | 0.11 (0.02,0.76) | 0.09 (0.02,0.50) | 0.03 (0.00,0.40) |
| 0.58 (0.34,0.99) | 0.56 (0.33,0.96) | 0.51 (0.32,0.80) | 0.43 (0.25,0.74) | 0.36 (0.11,1.20) | 0.32 (0.10,1.01) | 0.31 (0.15,0.67) | 0.18 (0.06,0.55) | 0.15 (0.07,0.29) | 0.05 (0.01,0.39) |
| 0.67 (0.43,1.04) | 0.64 (0.41,1.02) | 0.58 (0.41,0.83) | 0.49 (0.31,0.78) | 0.41 (0.13,1.33) | 0.37 (0.12,1.12) | 0.36 (0.18,0.72) | 0.21 (0.07,0.60) | 0.17 (0.09,0.31) | 0.05 (0.01,0.44) |
| 0.70 (0.46,1.05) | 0.67 (0.44,1.03) | 0.61 (0.45,0.83) | 0.52 (0.33,0.79) | 0.43 (0.13,1.37) | 0.39 (0.13,1.15) | 0.38 (0.19,0.74) | 0.22 (0.08,0.62) | 0.18 (0.10,0.32) | 0.06 (0.01,0.46) |
| 0.93 (0.37,2.31) | 0.89 (0.33,2.42) | 0.81 (0.31,2.10) | 0.68 (0.25,1.86) | 0.57 (0.13,2.47) | 0.52 (0.13,2.01) | 0.50 (0.16,1.55) | 0.29 (0.07,1.16) | 0.23 (0.08,0.64) | 0.08 (0.01,0.73) |
| 0.97 (0.29,3.18) | 0.93 (0.28,3.08) | 18.78 (2.13,165.56) | 0.71 (0.22,2.37) | 0.24 (0.07,0.86) | 0.54 (0.11,2.56) | 0.52 (0.14,1.93) | 0.31 (0.07,1.41) | 0.10 (0.01,0.87) | 0.59 (0.12,2.97) |
| 0.97 (0.48,1.97) | 0.93 (0.46,1.91) | 1.18 (0.61,2.28) | 0.71 (0.35,1.47) | 0.59 (0.16,2.18) | 0.54 (0.16,1.85) | 0.52 (0.21,1.27) | 0.31 (0.09,1.01) | 0.24 (0.11,0.56) | 0.08 (0.01,0.68) |
| Pazo | 0.97 (0.65,1.43) | 0.87 (0.67,1.14) | 0.74 (0.49,1.11) | 0.61 (0.19,1.94) | 0.56 (0.20,1.53) | 0.54 (0.28,1.04) | 0.32 (0.11,0.88) | 0.25 (0.16,0.39) | 0.08 (0.01,0.65) |
| 1.03 (0.70,1.53) | AtezoBev | 0.90 (0.68,1.20) | 0.76 (0.51,1.16) | 0.63 (0.20,2.02) | 0.58 (0.20,1.70) | 0.56 (0.29,1.09) | 0.33 (0.12,0.92) | 0.26 (0.15,0.47) | 0.08 (0.01,0.67) |
| 1.14 (0.87,1.50) | 1.11 (0.83,1.47) | Suni | 0.85 (0.63,1.14) | 0.70 (0.23,2.16) | 0.64 (0.22,1.81) | 0.62 (0.34,1.13) | 0.36 (0.14,0.98) | 0.29 (0.17,0.48) | 0.09 (0.01,0.73) |
| 1.35 (0.90,2.03) | 1.31 (0.86,1.98) | 1.18 (0.87,1.60) | Nivolpi | 0.83 (0.26,2.65) | 0.75 (0.25,2.23) | 0.73 (0.37,1.43) | 0.43 (0.17,1.10) | 0.34 (0.19,0.62) | 0.11 (0.01,0.88) |
| 1.64 (0.51,5.20) | 1.58 (0.50,5.04) | 1.43 (0.46,4.40) | 1.21 (0.38,3.87) | Savo | 0.91 (0.20,4.22) | 0.88 (0.25,3.16) | 0.52 (0.12,2.32) | 0.41 (0.12,1.42) | 0.13 (0.01,1.39) |
| 1.80 (0.66,4.92) | 1.74 (0.59,5.12) | 1.57 (0.55,4.45) | 1.33 (0.45,3.93) | 1.10 (0.24,5.09) | Bev | 0.97 (0.29,3.23) | 0.57 (0.14,2.40) | 0.45 (0.18,1.12) | 0.15 (0.01,1.47) |
| 1.85 (0.96,3.58) | 1.79 (0.92,3.48) | 1.62 (0.89,2.95) | 1.37 (0.70,2.68) | 1.13 (0.32,4.05) | 1.03 (0.31,3.44) | Atezo | 0.59 (0.19,1.87) | 0.47 (0.21,1.03) | 0.15 (0.02,1.29) |
| 3.15 (1.13,8.75) | 3.04 (1.09,8.48) | 2.75 (1.03,7.37) | 2.32 (0.91,5.95) | 1.92 (0.43,8.58) | 1.75 (0.42,7.36) | 1.70 (0.54,5.39) | Nivo | 0.79 (0.26,2.41) | 0.26 (0.03,2.51) |
| 3.97 (2.57,6.12) | 3.84 (2.14,6.89) | 3.47 (2.08,5.78) | 2.93 (1.62,5.31) | 2.43 (0.71,8.35) | 2.21 (0.89,5.49) | 2.14 (0.97,4.72) | 1.26 (0.42,3.83) | Placebo | 0.33 (0.04,2.70) |
| 12.19 (1.54,96.51) | 11.78 (1.48,93.43) | 10.65 (1.37,82.82) | 9.01 (1.13,71.61) | 7.45 (0.72,77.32) | 6.79 (0.68,67.80) | 6.58 (0.78,55.82) | 3.87 (0.40,37.72) | 3.07 (0.37,25.43) | Sora |

Abbreviations: Suni: Sunitinib; Bev: Bevacizumab; Cabo: Cabozantinib; Pazo: Pazopanib; Savo: Savolitinib; Sora: Sorafenib; Axi: Axitinib; Anlo: Anlotinib; Nivo: Nivolumab; Atezo: Atezolizumab; PemAxi: Pembrolizumab plus Axitinib; AtezoBev: Atezolizumab plus Bevacizumab; AveAxi: Avelumab plus Axitinib; LenPem: Lenvatinib plus Pembrolizumab; NivoCabo: Nivolumab plus Cabozantinib; NivoIpi: Nivolumab plus lpilimumab; LenEvero: Lenvatinib plus Everolimus; PazoEvero: Pazopanib plus Everolimus; IMA901Suni: IMA901 plus Sunitinib. Yellow suggests a relative treatment benefit, blue suggests a relative treatment harm, dark red suggests not significant.

Supplementary Table 2B. Matrix of pairwise comparisons of regimens on 6 month progression free survival (shown as odds ratio and 95% confidence intervals).

| LenPem | 0.56 (0.34,0.94) | 0.52 (0.27,0.99) | 0.50 (0.18,1.42) | 0.38 (0.24,0.62) | 0.36 (0.23,0.57) | 0.34 (0.21,0.54) | 0.36 (0.10,1.30) | 0.34 (0.11,0.99) | 0.31 (0.16,0.62) |
| --- | --- | --- | --- | --- | --- | --- | --- | --- | --- |
| 1.77 (1.07,2.94) | NivoCabo | 0.92 (0.49,1.73) | 0.89 (0.32,2.49) | 0.68 (0.43,1.08) | 0.64 (0.41,1.00) | 0.60 (0.38,0.94) | 0.64 (0.18,2.30) | 0.59 (0.20,1.75) | 0.55 (0.28,1.10) |
| 1.93 (1.01,3.66) | 1.09 (0.58,2.05) | Axi | 0.97 (0.32,2.92) | 0.74 (0.40,1.36) | 0.70 (0.39,1.27) | 0.65 (0.36,1.19) | 0.70 (0.23,2.11) | 0.65 (0.20,2.04) | 0.60 (0.47,0.78) |
| 1.99 (0.71,5.59) | 1.12 (0.40,3.14) | 1.03 (0.34,3.10) | Cabo | 0.76 (0.28,2.10) | 0.72 (0.26,1.97) | 0.67 (0.25,1.85) | 0.72 (0.15,3.44) | 0.67 (0.16,2.72) | 0.62 (0.20,1.93) |
| 2.61 (1.62,4.21) | 1.47 (0.92,2.35) | 1.35 (0.73,2.50) | 1.31 (0.48,3.63) | LenEvero | 0.95 (0.63,1.43) | 0.89 (0.58,1.35) | 0.95 (0.27,3.35) | 0.88 (0.30,2.55) | 0.82 (0.42,1.58) |
| 2.76 (1.75,4.35) | 1.56 (1.00,2.43) | 1.43 (0.79,2.59) | 1.39 (0.51,3.79) | 1.06 (0.70,1.59) | AVEAxi | 0.93 (0.63,1.39) | 1.00 (0.29,3.51) | 0.92 (0.32,2.66) | 0.86 (0.45,1.65) |
| 2.95 (1.85,4.69) | 1.67 (1.06,2.62) | 1.53 (0.84,2.79) | 1.48 (0.54,4.07) | 1.13 (0.74,1.72) | 1.07 (0.72,1.59) | PemAxi | 1.07 (0.30,3.76) | 0.99 (0.34,2.86) | 0.92 (0.48,1.77) |
| 2.76 (0.77,9.89) | 1.56 (0.44,5.56) | 1.43 (0.47,4.32) | 1.39 (0.29,6.60) | 1.06 (0.30,3.73) | 1.00 (0.29,3.51) | 0.93 (0.27,3.29) | Bev | 0.92 (0.19,4.56) | 0.86 (0.29,2.53) |
| 2.98 (1.01,8.83) | 1.68 (0.57,4.96) | 1.55 (0.49,4.89) | 1.50 (0.37,6.13) | 1.14 (0.39,3.32) | 1.08 (0.38,3.12) | 1.01 (0.35,2.92) | 1.08 (0.22,5.33) | Savo | 0.93 (0.29,3.03) |
| 3.20 (1.60,6.37) | 1.80 (0.91,3.57) | 1.66 (1.29,2.14) | 1.61 (0.52,4.98) | 1.22 (0.63,2.37) | 1.16 (0.61,2.21) | 1.08 (0.56,2.08) | 1.16 (0.40,3.40) | 1.07 (0.33,3.48) | Sora |
| 3.70 (2.38,5.76) | 2.09 (1.36,3.21) | 1.92 (1.07,3.44) | 1.86 (0.69,5.06) | 1.42 (0.95,2.11) | 1.34 (0.93,1.94) | 1.26 (0.86,1.83) | 1.34 (0.39,4.69) | 1.24 (0.43,3.56) | 1.16 (0.61,2.19) |
| 3.69 (1.43,9.53) | 2.08 (0.81,5.35) | 1.91 (0.69,5.32) | 1.86 (0.50,6.85) | 1.41 (0.56,3.58) | 1.34 (0.53,3.35) | 1.25 (0.50,3.14) | 1.34 (0.30,6.03) | 0.41 (0.14,1.22) | 0.83 (0.34,1.98) |
| 4.47 (3.10,6.44) | 2.52 (1.78,3.58) | 2.32 (1.37,3.93) | 2.25 (0.85,5.92) | 1.71 (1.26,2.33) | 1.62 (1.23,2.13) | 1.52 (1.14,2.02) | 1.62 (0.48,5.52) | 1.50 (0.54,4.17) | 1.40 (0.78,2.51) |
| 4.65 (2.43,8.92) | 2.63 (1.38,4.99) | 2.41 (1.14,5.13) | 2.34 (0.77,7.09) | 1.78 (0.96,3.32) | 1.69 (0.92,3.09) | 1.58 (0.86,2.90) | 1.69 (0.44,6.43) | 1.56 (0.49,4.96) | 1.46 (0.66,3.22) |
| 5.06 (3.30,7.75) | 2.86 (1.89,4.32) | 2.62 (1.48,4.65) | 2.54 (0.94,6.87) | 1.94 (1.33,2.83) | 1.84 (1.29,2.61) | 1.71 (1.20,2.46) | 1.84 (0.53,6.37) | 1.70 (0.60,4.83) | 1.58 (0.85,2.96) |
| 5.25 (3.37,8.19) | 2.97 (1.93,4.56) | 2.73 (1.52,4.89) | 2.64 (0.97,7.18) | 2.01 (1.35,3.00) | 1.91 (1.31,2.76) | 1.78 (1.22,2.61) | 1.91 (0.55,6.66) | 1.76 (0.61,5.05) | 1.64 (0.87,3.11) |
| 6.45 (2.59,16.08) | 3.64 (1.47,9.02) | 3.35 (1.24,9.00) | 3.25 (0.90,11.67) | 2.47 (1.01,6.03) | 2.34 (0.97,5.65) | 2.19 (0.90,5.30) | 2.34 (0.53,10.32) | 2.16 (0.58,8.11) | 2.02 (0.73,5.61) |
| 6.71 (3.45,13.05) | 3.79 (1.96,7.30) | 3.48 (1.62,7.49) | 3.37 (1.11,10.30) | 2.57 (1.36,4.85) | 2.43 (1.31,4.52) | 2.27 (1.22,4.25) | 2.43 (0.63,9.34) | 2.25 (0.70,7.21) | 2.10 (0.94,4.71) |
| 9.04 (4.27,19.11) | 5.10 (2.43,10.71) | 4.69 (3.19,6.90) | 4.55 (1.41,14.62) | 3.46 (1.68,7.13) | 3.28 (1.61,6.66) | 3.06 (1.50,6.25) | 3.28 (1.16,9.24) | 3.03 (0.90,10.20) | 2.83 (2.11,3.78) |
| 9.94 (3.73,26.46) | 5.61 (2.12,14.85) | 5.15 (1.80,14.74) | 5.00 (1.33,18.86) | 3.81 (1.46,9.94) | 3.61 (1.40,9.32) | 3.37 (1.30,8.73) | 3.61 (0.78,16.57) | 3.33 (0.85,13.09) | 3.11 (1.05,9.16) |

Supplementary Table 2B (continued)

| 0.27 (0.17,0.42) | 0.27 (0.10,0.70) | 0.22 (0.16,0.32) | 0.21 (0.11,0.41) | 0.20 (0.13,0.30) | 0.19 (0.12,0.30) | 0.16 (0.06,0.39) | 0.15 (0.08,0.29) | 0.11 (0.05,0.23) | 0.10 (0.04,0.27) |
| --- | --- | --- | --- | --- | --- | --- | --- | --- | --- |
| 0.48 (0.31,0.73) | 0.48 (0.19,1.23) | 0.40 (0.28,0.56) | 0.38 (0.20,0.72) | 0.35 (0.23,0.53) | 0.34 (0.22,0.52) | 0.27 (0.11,0.68) | 0.26 (0.14,0.51) | 0.20 (0.09,0.41) | 0.18 (0.07,0.47) |
| 0.52 (0.29,0.93) | 0.52 (0.19,1.45) | 0.43 (0.25,0.73) | 0.41 (0.19,0.88) | 0.38 (0.22,0.68) | 0.37 (0.20,0.66) | 0.30 (0.11,0.80) | 0.29 (0.13,0.62) | 0.21 (0.14,0.31) | 0.19 (0.07,0.55) |
| 0.54 (0.20,1.46) | 0.54 (0.15,1.99) | 0.44 (0.17,1.17) | 0.43 (0.14,1.29) | 0.39 (0.15,1.06) | 0.38 (0.14,1.03) | 0.31 (0.09,1.11) | 0.30 (0.10,0.90) | 0.22 (0.07,0.71) | 0.20 (0.05,0.75) |
| 0.71 (0.47,1.05) | 0.71 (0.28,1.79) | 0.58 (0.43,0.80) | 0.56 (0.30,1.04) | 0.52 (0.35,0.75) | 0.50 (0.33,0.74) | 0.40 (0.17,0.99) | 0.39 (0.21,0.74) | 0.29 (0.14,0.60) | 0.26 (0.10,0.69) |
| 0.74 (0.51,1.08) | 0.75 (0.30,1.87) | 0.62 (0.47,0.81) | 0.59 (0.32,1.08) | 0.54 (0.38,0.77) | 0.52 (0.36,0.76) | 0.43 (0.18,1.03) | 0.41 (0.22,0.76) | 0.30 (0.15,0.62) | 0.28 (0.11,0.72) |
| 0.80 (0.55,1.16) | 0.80 (0.32,2.01) | 0.66 (0.50,0.88) | 0.63 (0.34,1.17) | 0.58 (0.41,0.84) | 0.56 (0.38,0.82) | 0.46 (0.19,1.11) | 0.44 (0.24,0.82) | 0.33 (0.16,0.67) | 0.30 (0.11,0.77) |
| 0.74 (0.21,2.60) | 0.75 (0.17,3.37) | 0.62 (0.18,2.10) | 0.59 (0.16,2.26) | 0.54 (0.16,1.89) | 0.52 (0.15,1.83) | 0.43 (0.10,1.88) | 0.41 (0.11,1.58) | 0.30 (0.11,0.86) | 0.28 (0.06,1.27) |
| 0.81 (0.28,2.31) | 2.45 (0.82,7.31) | 0.67 (0.24,1.85) | 0.64 (0.20,2.04) | 0.59 (0.21,1.68) | 0.57 (0.20,1.63) | 0.46 (0.12,1.73) | 0.44 (0.14,1.42) | 0.33 (0.10,1.11) | 0.30 (0.08,1.18) |
| 0.86 (0.46,1.63) | 1.21 (0.50,2.91) | 0.71 (0.40,1.28) | 0.69 (0.31,1.52) | 0.63 (0.34,1.18) | 0.61 (0.32,1.15) | 0.50 (0.18,1.38) | 0.48 (0.21,1.07) | 0.35 (0.26,0.47) | 0.32 (0.11,0.95) |
| AtezoBev | 1.00 (0.40,2.49) | 0.83 (0.65,1.06) | 0.80 (0.44,1.44) | 0.73 (0.53,1.02) | 0.70 (0.50,1.00) | 0.57 (0.24,1.38) | 0.55 (0.30,1.02) | 0.41 (0.20,0.82) | 0.37 (0.15,0.96) |
| 1.00 (0.40,2.48) | Anlo | 1.24 (0.32,4.76) | 0.79 (0.28,2.22) | 0.73 (0.30,1.80) | 0.70 (0.28,1.75) | 0.57 (0.17,1.92) | 0.55 (0.19,1.55) | 0.54 (0.24,1.21) | 0.37 (0.11,1.31) |
| 1.21 (0.94,1.55) | 0.81 (0.21,3.11) | Suni | 1.04 (0.61,1.78) | 0.88 (0.71,1.10) | 0.85 (0.66,1.09) | 0.69 (0.30,1.60) | 0.67 (0.38,1.16) | 0.49 (0.26,0.95) | 0.45 (0.18,1.12) |
| 1.26 (0.69,2.27) | 1.26 (0.45,3.53) | 0.96 (0.56,1.65) | IMA901Suni | 0.92 (0.51,1.65) | 0.89 (0.49,1.60) | 0.72 (0.27,1.95) | 0.69 (0.32,1.51) | 0.51 (0.22,1.20) | 0.47 (0.16,1.35) |
| 1.37 (0.98,1.90) | 1.37 (0.56,3.38) | 1.13 (0.91,1.41) | 1.09 (0.61,1.95) | Pazo | 0.96 (0.69,1.34) | 0.78 (0.35,1.76) | 0.75 (0.41,1.37) | 0.56 (0.28,1.12) | 0.51 (0.20,1.30) |
| 1.42 (1.00,2.02) | 1.42 (0.57,3.54) | 1.17 (0.91,1.51) | 1.13 (0.62,2.05) | 1.04 (0.74,1.45) | Nivolpi | 0.81 (0.34,1.95) | 0.78 (0.43,1.44) | 0.58 (0.29,1.17) | 0.53 (0.22,1.27) |
| 1.74 (0.73,4.17) | 1.75 (0.52,5.87) | 1.44 (0.62,3.33) | 1.39 (0.51,3.75) | 1.28 (0.57,2.86) | 1.23 (0.51,2.94) | PazoEvero | 0.96 (0.35,2.63) | 0.71 (0.25,2.07) | 0.65 (0.19,2.23) |
| 1.81 (0.98,3.33) | 1.82 (0.64,5.13) | 1.50 (0.86,2.62) | 1.44 (0.66,3.13) | 1.33 (0.73,2.41) | 1.28 (0.69,2.35) | 1.04 (0.38,2.84) | Atezo | 0.74 (0.31,1.75) | 0.67 (0.23,1.96) |
| 2.44 (1.21,4.91) | 1.86 (0.83,4.20) | 2.02 (1.05,3.89) | 1.94 (0.83,4.53) | 1.79 (0.90,3.56) | 1.72 (0.85,3.47) | 1.40 (0.48,4.05) | 1.35 (0.57,3.18) | Placebo | 0.91 (0.30,2.79) |
| 2.68 (1.05,6.88) | 2.69 (0.76,9.52) | 2.22 (0.90,5.51) | 2.14 (0.74,6.14) | 1.96 (0.77,5.01) | 1.89 (0.79,4.53) | 1.54 (0.45,5.30) | 1.48 (0.51,4.30) | 1.10 (0.36,3.37) | Nivo |

Abbreviations: Suni: Sunitinib; Bev: Bevacizumab; Cabo: Cabozantinib; Pazo: Pazopanib; Savo: Savolitinib; Sora: Sorafenib; Axi: Axitinib; Anlo: Anlotinib; Nivo: Nivolumab; Atezo: Atezolizumab; PemAxi: Pembrolizumab plus Axitinib; AtezoBev: Atezolizumab plus Bevacizumab; AveAxi: Avelumab plus Axitinib; LenPem: Lenvatinib plus Pembrolizumab; NivoCabo: Nivolumab plus Cabozantinib; NivoIpi: Nivolumab plus lpilimumab; LenEvero: Lenvatinib plus Everolimus; PazoEvero: Pazopanib plus Everolimus; IMA901Suni: IMA901 plus Sunitinib. Yellow suggests a relative treatment benefit, blue suggests a relative treatment harm, dark red suggests not significant.

Supplementary Table 2C. Matrix of pairwise comparisons of regimens on 12 month progression free survival (shown as odds ratio and 95% confidence intervals).

| LenPem | 1.78 (0.18,17.46) | 0.82 (0.44,1.52) | 0.63 (0.40,0.98) | 0.50 (0.33,0.78) | 0.45 (0.30,0.67) | 0.48 (0.15,1.52) | 0.44 (0.29,0.67) | 0.41 (0.21,0.82) | 0.37 (0.15,0.92) |
| --- | --- | --- | --- | --- | --- | --- | --- | --- | --- |
| 0.56 (0.06,5.48) | Bev | 0.46 (0.05,4.12) | 0.35 (0.04,3.46) | 0.28 (0.03,2.76) | 0.25 (0.03,2.42) | 0.27 (0.02,3.32) | 0.25 (0.03,2.41) | 0.23 (0.03,2.04) | 0.21 (0.02,2.34) |
| 1.22 (0.66,2.27) | 2.18 (0.24,19.56) | Axi | 0.77 (0.41,1.43) | 0.62 (0.33,1.13) | 0.54 (0.30,0.98) | 0.58 (0.17,2.00) | 0.54 (0.30,0.98) | 0.50 (0.38,0.67) | 0.46 (0.17,1.23) |
| 1.59 (1.02,2.47) | 2.83 (0.29,27.70) | 1.30 (0.70,2.42) | NivoCabo | 0.80 (0.52,1.23) | 0.71 (0.47,1.06) | 0.76 (0.24,2.40) | 0.70 (0.46,1.06) | 0.66 (0.33,1.29) | 0.59 (0.24,1.45) |
| 1.98 (1.29,3.05) | 3.54 (0.36,34.56) | 1.62 (0.88,3.00) | 1.25 (0.81,1.93) | LenEvero | 0.88 (0.59,1.31) | 0.95 (0.30,2.99) | 0.88 (0.59,1.31) | 0.82 (0.42,1.61) | 0.74 (0.31,1.81) |
| 2.25 (1.50,3.37) | 4.01 (0.41,38.97) | 1.84 (1.02,3.33) | 1.42 (0.94,2.13) | 1.13 (0.76,1.68) | PemAxi | 1.08 (0.35,3.36) | 0.99 (0.69,1.44) | 0.93 (0.48,1.79) | 0.84 (0.35,2.02) |
| 2.09 (0.66,6.60) | 3.72 (0.30,46.13) | 1.71 (0.50,5.85) | 1.32 (0.42,4.17) | 1.05 (0.33,3.32) | 0.93 (0.30,2.90) | Savo | 0.92 (0.30,2.89) | 0.86 (0.24,3.05) | 0.78 (0.19,3.14) |
| 2.26 (1.50,3.41) | 4.04 (0.41,39.25) | 1.85 (1.02,3.37) | 1.43 (0.94,2.16) | 1.14 (0.76,1.70) | 1.01 (0.69,1.46) | 1.08 (0.35,3.39) | AveAxi | 0.93 (0.48,1.81) | 0.85 (0.35,2.04) |
| 2.42 (1.23,4.78) | 4.32 (0.49,38.10) | 1.98 (1.50,2.63) | 1.53 (0.77,3.02) | 1.22 (0.62,2.39) | 1.08 (0.56,2.08) | 1.16 (0.33,4.10) | 1.07 (0.55,2.07) | Sora | 0.91 (0.32,2.55) |
| 2.67 (1.09,6.53) | 4.77 (0.43,53.04) | 2.19 (0.81,5.91) | 1.68 (0.69,4.12) | 1.35 (0.55,3.28) | 1.19 (0.49,2.86) | 1.28 (0.32,5.13) | 1.18 (0.49,2.85) | 1.10 (0.39,3.10) | PazoEvero |
| 2.79 (1.30,5.97) | 4.97 (0.47,52.85) | 2.28 (0.95,5.48) | 1.76 (0.82,3.77) | 1.40 (0.66,2.99) | 1.24 (0.59,2.60) | 1.33 (0.36,4.94) | 1.23 (0.59,2.59) | 1.15 (0.46,2.89) | 1.04 (0.35,3.10) |
| 2.80 (1.23,6.41) | 5.00 (0.46,54.34) | 2.30 (0.90,5.84) | 1.77 (0.77,4.05) | 1.41 (0.62,3.21) | 1.25 (0.56,2.80) | 0.50 (0.17,1.45) | 1.24 (0.55,2.79) | 0.72 (0.34,1.55) | 1.05 (0.34,3.27) |
| 2.81 (1.90,4.17) | 5.02 (0.52,48.70) | 2.31 (1.29,4.14) | 1.78 (1.20,2.63) | 1.42 (0.97,2.08) | 1.25 (0.88,1.78) | 1.35 (0.43,4.19) | 1.24 (0.87,1.78) | 1.16 (0.61,2.22) | 1.05 (0.44,2.52) |
| 3.88 (2.84,5.30) | 6.92 (0.72,66.24) | 3.18 (1.86,5.42) | 2.44 (1.78,3.35) | 1.95 (1.45,2.63) | 1.72 (1.33,2.23) | 1.86 (0.61,5.63) | 1.71 (1.31,2.24) | 1.60 (0.88,2.93) | 1.45 (0.63,3.35) |
| 4.17 (2.21,7.89) | 7.45 (0.73,76.31) | 3.42 (1.58,7.39) | 2.63 (1.39,4.99) | 2.11 (1.12,3.95) | 1.86 (1.01,3.43) | 2.00 (0.58,6.91) | 1.85 (1.00,3.42) | 1.73 (0.76,3.92) | 1.56 (0.57,4.27) |
| 4.17 (2.82,6.17) | 7.44 (0.77,72.14) | 3.42 (1.90,6.13) | 2.63 (1.77,3.90) | 2.10 (1.44,3.08) | 1.86 (1.31,2.63) | 2.00 (0.64,6.20) | 1.84 (1.29,2.63) | 1.72 (0.90,3.30) | 1.56 (0.65,3.73) |
| 4.73 (2.74,8.14) | 8.43 (0.84,84.35) | 3.87 (1.93,7.76) | 2.98 (1.73,5.14) | 2.38 (1.39,4.07) | 2.10 (1.26,3.52) | 2.26 (0.69,7.48) | 2.09 (1.24,3.51) | 1.95 (0.92,4.13) | 1.77 (0.69,4.57) |
| 4.72 (3.22,6.92) | 8.42 (0.87,81.54) | 3.87 (2.17,6.89) | 2.98 (2.03,4.37) | 2.38 (1.64,3.45) | 2.10 (1.50,2.95) | 2.26 (0.73,7.00) | 2.09 (1.48,2.95) | 1.95 (1.03,3.71) | 1.77 (0.79,3.97) |
| 6.09 (2.19,16.92) | 10.87 (0.93,127.23) | 4.99 (1.65,15.14) | 3.84 (1.38,10.68) | 3.07 (1.11,8.50) | 2.71 (0.99,7.42) | 2.92 (0.67,12.75) | 2.69 (0.98,7.39) | 2.52 (0.80,7.91) | 2.28 (0.63,8.24) |
| 5.64 (2.51,12.69) | 10.06 (1.19,84.83) | 4.62 (2.74,7.80) | 3.56 (1.58,8.01) | 2.84 (1.27,6.37) | 2.51 (1.14,5.54) | 2.70 (0.71,10.29) | 2.49 (1.13,5.52) | 2.33 (1.50,3.63) | 2.11 (0.69,6.49) |

Supplementary Table 2C (continued)

| 0.36 (0.17,0.77) | 0.36 (0.16,0.82) | 0.36 (0.24,0.53) | 0.26 (0.19,0.35) | 0.24 (0.13,0.45) | 0.24 (0.16,0.36) | 0.21 (0.12,0.36) | 0.21 (0.14,0.31) | 0.16 (0.06,0.46) | 0.18 (0.08,0.40) |
| --- | --- | --- | --- | --- | --- | --- | --- | --- | --- |
| 0.20 (0.02,2.14) | 0.20 (0.02,2.17) | 0.20 (0.02,1.93) | 0.14 (0.02,1.38) | 0.13 (0.01,1.37) | 0.13 (0.01,1.30) | 0.12 (0.01,1.19) | 0.12 (0.01,1.15) | 0.09 (0.01,1.08) | 0.10 (0.01,0.84) |
| 0.44 (0.18,1.05) | 0.44 (0.17,1.11) | 0.43 (0.24,0.78) | 0.31 (0.18,0.54) | 0.29 (0.14,0.63) | 0.29 (0.16,0.53) | 0.26 (0.13,0.52) | 0.26 (0.15,0.46) | 0.20 (0.07,0.61) | 0.22 (0.13,0.37) |
| 0.57 (0.27,1.22) | 0.57 (0.25,1.29) | 0.56 (0.38,0.84) | 0.41 (0.30,0.56) | 0.38 (0.20,0.72) | 0.38 (0.26,0.56) | 0.34 (0.19,0.58) | 0.34 (0.23,0.49) | 0.26 (0.09,0.72) | 0.28 (0.12,0.63) |
| 0.71 (0.33,1.52) | 0.71 (0.31,1.61) | 0.70 (0.48,1.03) | 0.51 (0.38,0.69) | 0.47 (0.25,0.89) | 0.48 (0.32,0.70) | 0.42 (0.25,0.72) | 0.42 (0.29,0.61) | 0.33 (0.12,0.90) | 0.35 (0.16,0.79) |
| 0.81 (0.38,1.69) | 0.80 (0.36,1.80) | 0.80 (0.56,1.13) | 0.58 (0.45,0.75) | 0.54 (0.29,0.99) | 0.54 (0.38,0.77) | 0.48 (0.28,0.80) | 0.48 (0.34,0.67) | 0.37 (0.13,1.01) | 0.40 (0.18,0.88) |
| 0.75 (0.20,2.77) | 2.01 (0.69,5.87) | 0.74 (0.24,2.30) | 0.54 (0.18,1.63) | 0.50 (0.14,1.73) | 0.50 (0.16,1.55) | 0.44 (0.13,1.46) | 0.44 (0.14,1.37) | 0.34 (0.08,1.50) | 0.37 (0.10,1.41) |
| 0.81 (0.39,1.71) | 0.81 (0.36,1.81) | 0.80 (0.56,1.15) | 0.58 (0.45,0.76) | 0.54 (0.29,1.00) | 0.54 (0.38,0.77) | 0.48 (0.28,0.80) | 0.48 (0.34,0.68) | 0.37 (0.14,1.02) | 0.40 (0.18,0.89) |
| 0.87 (0.35,2.18) | 1.38 (0.64,2.97) | 0.86 (0.45,1.64) | 0.62 (0.34,1.14) | 0.58 (0.26,1.32) | 0.58 (0.30,1.11) | 0.51 (0.24,1.08) | 0.51 (0.27,0.97) | 0.40 (0.13,1.25) | 0.43 (0.28,0.67) |
| 0.96 (0.32,2.85) | 0.95 (0.31,2.96) | 0.95 (0.40,2.27) | 0.69 (0.30,1.59) | 0.64 (0.23,1.75) | 0.64 (0.27,1.53) | 0.57 (0.22,1.46) | 0.57 (0.25,1.27) | 0.44 (0.12,1.58) | 0.47 (0.15,1.46) |
| Cabo | 0.99 (0.35,2.79) | 0.99 (0.47,2.06) | 0.72 (0.36,1.44) | 0.67 (0.27,1.62) | 0.67 (0.32,1.39) | 0.59 (0.26,1.35) | 0.59 (0.28,1.22) | 0.46 (0.14,1.51) | 0.49 (0.18,1.37) |
| 1.01 (0.36,2.83) | Anlo | 1.00 (0.45,2.22) | 1.34 (0.35,5.16) | 0.67 (0.26,1.73) | 0.67 (0.30,1.50) | 0.59 (0.24,1.44) | 0.59 (0.27,1.32) | 0.46 (0.13,1.59) | 0.28 (0.11,0.71) |
| 1.01 (0.48,2.11) | 1.00 (0.45,2.24) | AtezoBev | 0.73 (0.57,0.92) | 0.67 (0.37,1.23) | 0.68 (0.48,0.94) | 0.60 (0.36,0.99) | 0.60 (0.43,0.82) | 0.46 (0.17,1.26) | 0.50 (0.23,1.09) |
| 1.39 (0.69,2.79) | 0.74 (0.19,2.86) | 1.38 (1.09,1.74) | Suni | 0.93 (0.53,1.62) | 0.93 (0.73,1.18) | 1.22 (0.78,1.90) | 0.82 (0.66,1.02) | 0.64 (0.24,1.68) | 0.69 (0.33,1.45) |
| 1.50 (0.62,3.65) | 1.49 (0.58,3.83) | 1.48 (0.81,2.71) | 1.08 (0.62,1.88) | Atezo | 1.00 (0.55,1.83) | 0.88 (0.43,1.80) | 0.88 (0.49,1.61) | 0.69 (0.22,2.10) | 0.74 (0.29,1.88) |
| 1.50 (0.72,3.12) | 1.49 (0.67,3.31) | 1.48 (1.06,2.07) | 1.08 (0.85,1.36) | 1.00 (0.55,1.83) | Nivolpi | 0.88 (0.53,1.46) | 0.88 (0.64,1.22) | 0.68 (0.27,1.76) | 0.74 (0.34,1.62) |
| 1.70 (0.74,3.87) | 1.69 (0.70,4.09) | 1.68 (1.01,2.78) | 0.82 (0.53,1.28) | 1.13 (0.56,2.31) | 1.13 (0.68,1.88) | IMA901Suni | 1.00 (0.61,1.64) | 0.78 (0.27,2.26) | 0.84 (0.35,2.00) |
| 1.69 (0.82,3.51) | 1.68 (0.76,3.73) | 1.68 (1.21,2.32) | 1.22 (0.98,1.52) | 1.13 (0.62,2.05) | 1.13 (0.82,1.56) | 1.00 (0.61,1.64) | Pazo | 0.77 (0.29,2.10) | 0.84 (0.38,1.83) |
| 2.19 (0.66,7.23) | 2.17 (0.63,7.49) | 2.16 (0.80,5.89) | 1.57 (0.59,4.16) | 1.46 (0.48,4.47) | 1.46 (0.57,3.75) | 1.29 (0.44,3.76) | 1.29 (0.48,3.50) | Nivo | 1.08 (0.32,3.69) |
| 2.02 (0.73,5.62) | 3.53 (1.40,8.88) | 2.00 (0.91,4.39) | 1.45 (0.69,3.08) | 1.35 (0.53,3.43) | 1.35 (0.62,2.97) | 1.19 (0.50,2.85) | 1.19 (0.55,2.61) | 0.93 (0.27,3.16) | Placebo |

Abbreviations: Suni: Sunitinib; Bev: Bevacizumab; Cabo: Cabozantinib; Pazo: Pazopanib; Savo: Savolitinib; Sora: Sorafenib; Axi: Axitinib; Anlo: Anlotinib; Nivo: Nivolumab; Atezo: Atezolizumab; PemAxi: Pembrolizumab plus Axitinib; AtezoBev: Atezolizumab plus Bevacizumab; AveAxi: Avelumab plus Axitinib; LenPem: Lenvatinib plus Pembrolizumab; NivoCabo: Nivolumab plus Cabozantinib; NivoIpi: Nivolumab plus lpilimumab; LenEvero: Lenvatinib plus Everolimus; PazoEvero: Pazopanib plus Everolimus; IMA901Suni: IMA901 plus Sunitinib. Yellow suggests a relative treatment benefit, blue suggests a relative treatment harm, dark red suggests not significant.

Supplementary Table 2D. Matrix of pairwise comparisons of regimens on 18 month progression free survival (shown as odds ratio and 95% confidence intervals).

| Axi | 0.98 (0.47,2.07) | 0.83 (0.39,1.77) | 0.79 (0.52,1.21) | 0.64 (0.30,1.33) | 0.59 (0.29,1.21) | 0.54 (0.26,1.14) | 0.51 (0.25,1.05) |
| --- | --- | --- | --- | --- | --- | --- | --- |
| 1.02 (0.48,2.14) | LenPem | 0.84 (0.46,1.55) | 0.81 (0.34,1.90) | 0.65 (0.36,1.15) | 0.60 (0.34,1.04) | 0.55 (0.30,0.99) | 0.52 (0.29,0.91) |
| 1.20 (0.57,2.56) | 1.18 (0.65,2.17) | NivoCabo | 0.95 (0.40,2.26) | 0.76 (0.42,1.38) | 0.71 (0.40,1.25) | 0.65 (0.35,1.19) | 0.61 (0.34,1.09) |
| 1.26 (0.83,1.91) | 1.24 (0.53,2.92) | 1.05 (0.44,2.48) | Sora | 0.80 (0.34,1.87) | 0.74 (0.32,1.70) | 0.68 (0.29,1.60) | 0.64 (0.28,1.47) |
| 1.57 (0.75,3.28) | 1.55 (0.87,2.76) | 1.31 (0.72,2.36) | 1.25 (0.54,2.91) | AveAxi | 0.93 (0.54,1.59) | 0.85 (0.48,1.52) | 0.80 (0.46,1.39) |
| 1.70 (0.83,3.49) | 1.67 (0.96,2.92) | 1.41 (0.80,2.50) | 1.35 (0.59,3.10) | 1.08 (0.63,1.86) | PemAxi | 0.92 (0.53,1.60) | 0.86 (0.50,1.49) |
| 1.85 (0.88,3.90) | 1.82 (1.01,3.30) | 1.54 (0.84,2.82) | 1.47 (0.63,3.46) | 1.18 (0.66,2.10) | 1.09 (0.62,1.90) | LenEvero | 0.94 (0.54,1.65) |
| 1.97 (0.95,4.06) | 1.94 (1.10,3.40) | 1.64 (0.92,2.91) | 1.56 (0.68,3.59) | 1.25 (0.72,2.17) | 1.16 (0.67,2.00) | 1.06 (0.60,1.87) | AtezoBev |
| 1.98 (0.62,6.36) | 1.95 (0.66,5.71) | 1.65 (0.56,4.86) | 1.57 (0.46,5.42) | 1.26 (0.43,3.66) | 1.17 (0.40,3.38) | 1.07 (0.36,3.14) | 1.01 (0.36,2.84) |
| 2.73 (1.33,5.62) | 2.69 (1.53,4.72) | 2.27 (1.28,4.03) | 2.17 (0.94,4.99) | 1.74 (1.00,3.00) | 1.61 (0.95,2.72) | 1.48 (0.84,2.59) | 1.39 (0.82,2.36) |
| 3.00 (1.62,5.56) | 2.95 (1.94,4.49) | 2.49 (1.61,3.85) | 2.38 (1.13,5.02) | 1.91 (1.28,2.84) | 1.76 (1.22,2.55) | 1.62 (1.07,2.46) | 1.52 (1.05,2.22) |
| 3.09 (1.22,7.85) | 3.04 (1.35,6.86) | 2.57 (1.13,5.85) | 2.46 (0.89,6.81) | 1.97 (0.88,4.39) | 1.82 (0.83,4.00) | 1.67 (0.74,3.77) | 1.57 (0.71,3.47) |
| 3.62 (1.04,12.62) | 3.56 (1.11,11.40) | 3.01 (0.93,9.69) | 2.87 (0.77,10.72) | 2.30 (0.72,7.32) | 2.13 (0.68,6.70) | 1.95 (0.61,6.26) | 1.84 (0.58,5.81) |
| 3.51 (1.45,8.53) | 3.45 (1.61,7.41) | 2.92 (1.35,6.32) | 0.29 (0.11,0.82) | 2.23 (1.05,4.74) | 2.07 (0.99,4.31) | 1.90 (0.88,4.07) | 1.78 (0.85,3.74) |
| 3.91 (1.74,8.77) | 3.84 (1.97,7.51) | 3.25 (1.64,6.41) | 3.10 (1.25,7.70) | 2.48 (1.28,4.79) | 2.30 (1.21,4.35) | 2.11 (1.08,4.12) | 1.98 (1.04,3.78) |
| 3.94 (1.93,8.05) | 3.88 (2.23,6.74) | 3.27 (1.86,5.77) | 3.13 (1.37,7.14) | 2.50 (1.46,4.29) | 2.32 (1.36,3.95) | 2.13 (1.22,3.70) | 2.00 (1.25,3.22) |

Table 2D (continued)

| 0.50 (0.16,1.62) | 0.37 (0.18,0.75) | 0.33 (0.18,0.62) | 0.32 (0.13,0.82) | 0.28 (0.08,0.96) | 0.28 (0.12,0.69) | 0.26 (0.11,0.57) | 0.25 (0.12,0.52) | |
| --- | --- | --- | --- | --- | --- | --- | --- | --- |
| 0.51 (0.18,1.50) | 0.37 (0.21,0.65) | 0.34 (0.22,0.52) | 0.33 (0.15,0.74) | 0.28 (0.09,0.90) | 0.29 (0.13,0.62) | 0.26 (0.13,0.51) | 0.26 (0.15,0.45) | |
| 0.61 (0.21,1.79) | 0.44 (0.25,0.78) | 0.40 (0.26,0.62) | 0.39 (0.17,0.88) | 0.33 (0.10,1.07) | 0.34 (0.16,0.74) | 0.31 (0.16,0.61) | 0.31 (0.17,0.54) | |
| 0.64 (0.18,2.19) | 0.46 (0.20,1.06) | 0.42 (0.20,0.89) | 0.41 (0.15,1.13) | 0.35 (0.09,1.30) | 3.39 (1.22,9.45) | 0.32 (0.13,0.80) | 0.32 (0.14,0.73) | |
| 0.79 (0.27,2.31) | 0.58 (0.33,1.00) | 0.52 (0.35,0.78) | 0.51 (0.23,1.14) | 0.43 (0.14,1.38) | 0.45 (0.21,0.95) | 0.40 (0.21,0.78) | 0.40 (0.23,0.68) | |
| 0.86 (0.30,2.49) | 0.62 (0.37,1.05) | 0.57 (0.39,0.82) | 0.55 (0.25,1.21) | 0.47 (0.15,1.48) | 0.48 (0.23,1.01) | 0.44 (0.23,0.82) | 0.43 (0.25,0.74) | |
| 0.94 (0.32,2.74) | 0.68 (0.39,1.19) | 0.62 (0.41,0.94) | 0.60 (0.27,1.35) | 0.51 (0.16,1.64) | 0.53 (0.25,1.13) | 0.47 (0.24,0.93) | 0.47 (0.27,0.82) | |
| 0.99 (0.35,2.80) | 0.72 (0.42,1.22) | 0.66 (0.45,0.96) | 0.64 (0.29,1.40) | 0.54 (0.17,1.72) | 0.56 (0.27,1.18) | 0.50 (0.26,0.96) | 0.50 (0.31,0.80) | |
| PazoEvero | 0.72 (0.25,2.09) | 0.66 (0.25,1.78) | 0.64 (0.19,2.15) | 0.55 (0.13,2.38) | 0.56 (0.17,1.83) | 0.51 (0.17,1.55) | 0.50 (0.20,1.26) | |
| 1.38 (0.48,3.98) | Nivolpi | 0.91 (0.63,1.32) | 0.88 (0.40,1.95) | 0.76 (0.27,2.09) | 0.78 (0.37,1.63) | 0.70 (0.37,1.33) | 0.69 (0.41,1.17) | |
| 1.51 (0.56,4.08) | 1.10 (0.76,1.59) | Suni | 0.97 (0.48,1.95) | 0.83 (0.28,2.45) | 0.85 (0.45,1.62) | 0.77 (0.46,1.30) | 0.76 (0.53,1.09) | |
| 1.56 (0.47,5.24) | 1.13 (0.51,2.49) | 1.03 (0.51,2.07) | Cabo | 0.85 (0.24,3.10) | 0.88 (0.34,2.26) | 0.79 (0.33,1.89) | 0.79 (0.36,1.72) | |
| 1.83 (0.42,7.94) | 1.32 (0.48,3.67) | 1.21 (0.41,3.57) | 1.17 (0.32,4.25) | Nivo | 1.03 (0.29,3.63) | 0.93 (0.28,3.09) | 0.92 (0.29,2.88) | |
| 1.77 (0.55,5.76) | 1.28 (0.61,2.69) | 1.17 (0.62,2.21) | 1.14 (0.44,2.92) | 0.97 (0.28,3.42) | Atezo | 0.90 (0.39,2.05) | 0.89 (0.43,1.85) | |
| 1.97 (0.64,6.04) | 1.43 (0.75,2.72) | 1.30 (0.77,2.20) | 1.26 (0.53,3.02) | 1.08 (0.32,3.60) | 1.11 (0.49,2.54) | IMA901Suni | 0.99 (0.53,1.87) | |
| 1.99 (0.79,5.00) | 1.44 (0.86,2.42) | 1.31 (0.92,1.88) | 1.27 (0.58,2.79) | 1.09 (0.35,3.42) | 1.12 (0.54,2.33) | 1.01 (0.53,1.90) | Pazo |  |

Abbreviations: Suni: Sunitinib; Cabo: Cabozantinib; Pazo: Pazopanib; Sora: Sorafenib; Axi: Axitinib; Nivo: Nivolumab; Atezo: Atezolizumab; PemAxi: Pembrolizumab plus Axitinib; AtezoBev: Atezolizumab plus Bevacizumab; AveAxi: Avelumab plus Axitinib; LenPem: Lenvatinib plus Pembrolizumab; NivoCabo: Nivolumab plus Cabozantinib; NivoIpi: Nivolumab plus lpilimumab; LenEvero: Lenvatinib plus Everolimus; PazoEvero: Pazopanib plus Everolimus; IMA901Suni: IMA901 plus Sunitinib. Yellow suggests a relative treatment benefit, blue suggests a relative treatment harm, dark red suggests not significant.

Supplementary Table 2E. Matrix of pairwise comparisons of regimens on 24 month progression free survival (shown as odds ratio and 95% confidence intervals).

| LenPem | 0.81 (0.42,1.58) | 0.72 (0.44,1.15) | 0.55 (0.36,0.85) | 0.49 (0.32,0.75) | 0.46 (0.29,0.74) | 0.43 (0.22,0.84) | 0.42 (0.21,0.86) |
| --- | --- | --- | --- | --- | --- | --- | --- |
| 1.23 (0.63,2.40) | Axi | 0.88 (0.45,1.74) | 0.68 (0.36,1.29) | 0.60 (0.32,1.15) | 0.57 (0.29,1.12) | 0.52 (0.23,1.21) | 0.52 (0.22,1.23) |
| 1.40 (0.87,2.25) | 1.13 (0.58,2.24) | NivoCabo | 0.77 (0.49,1.20) | 0.69 (0.44,1.07) | 0.65 (0.40,1.05) | 0.59 (0.30,1.19) | 0.59 (0.29,1.22) |
| 1.81 (1.18,2.79) | 1.47 (0.77,2.82) | 1.30 (0.83,2.03) | AveAxi | 0.89 (0.60,1.32) | 0.84 (0.55,1.31) | 0.77 (0.40,1.50) | 0.77 (0.39,1.53) |
| 2.03 (1.33,3.12) | 1.65 (0.87,3.15) | 1.46 (0.93,2.27) | 1.12 (0.76,1.66) | PemAxi | 0.95 (0.61,1.46) | 0.87 (0.45,1.67) | 0.86 (0.43,1.71) |
| 2.15 (1.35,3.43) | 1.75 (0.89,3.42) | 1.54 (0.95,2.50) | 1.19 (0.77,1.83) | 1.06 (0.69,1.63) | LenEvero | 0.92 (0.46,1.82) | 0.91 (0.45,1.86) |
| 2.35 (1.19,4.64) | 1.91 (0.83,4.40) | 1.68 (0.84,3.36) | 1.29 (0.67,2.51) | 1.15 (0.60,2.23) | 1.09 (0.55,2.17) | Atezo | 1.00 (0.42,2.37) |
| 2.36 (1.16,4.78) | 1.92 (0.81,4.52) | 1.69 (0.82,3.46) | 1.30 (0.65,2.59) | 1.16 (0.58,2.30) | 1.10 (0.54,2.24) | 1.00 (0.42,2.39) | Cabo |
| 2.89 (1.88,4.44) | 2.35 (1.23,4.49) | 2.07 (1.32,3.24) | 1.59 (1.07,2.37) | 1.42 (0.96,2.11) | 1.34 (0.87,2.08) | 1.23 (0.64,2.38) | 1.23 (0.62,2.44) |
| 3.00 (1.99,4.52) | 2.44 (1.29,4.60) | 2.15 (1.40,3.30) | 1.65 (1.14,2.41) | 1.48 (1.02,2.14) | 1.40 (0.92,2.12) | 1.28 (0.67,2.44) | 1.27 (0.65,2.50) |
| 3.18 (1.16,8.73) | 2.59 (0.84,7.92) | 2.28 (0.82,6.30) | 1.75 (0.65,4.74) | 1.56 (0.58,4.23) | 1.48 (0.54,4.07) | 1.35 (0.44,4.18) | 1.35 (0.43,4.23) |
| 3.46 (2.50,4.79) | 2.81 (1.57,5.04) | 2.48 (1.75,3.51) | 1.91 (1.44,2.52) | 1.70 (1.29,2.24) | 1.61 (1.15,2.25) | 1.47 (0.81,2.68) | 1.47 (0.78,2.75) |
| 4.13 (1.79,9.52) | 3.35 (1.28,8.81) | 2.95 (1.27,6.88) | 2.27 (1.00,5.16) | 2.03 (0.89,4.60) | 1.92 (0.83,4.45) | 1.76 (0.66,4.66) | 1.75 (0.65,4.73) |
| 4.86 (2.73,8.68) | 3.95 (1.86,8.41) | 3.48 (1.93,6.30) | 2.68 (1.54,4.67) | 2.39 (1.38,4.15) | 2.26 (1.26,4.06) | 2.07 (0.96,4.46) | 2.06 (0.94,4.55) |
| 5.03 (3.31,7.65) | 4.09 (2.16,7.76) | 3.60 (2.33,5.58) | 2.77 (1.89,4.07) | 2.47 (1.69,3.62) | 2.34 (1.53,3.58) | 2.14 (1.11,4.12) | 2.13 (1.08,4.22) |

Supplementary Table 2E (continued)

| 0.35 (0.23,0.53) | 0.33 (0.22,0.50) | 0.31 (0.11,0.86) | 0.29 (0.21,0.40) | 0.24 (0.11,0.56) | 0.21 (0.12,0.37) | 0.20 (0.13,0.30) |
| --- | --- | --- | --- | --- | --- | --- |
| 0.43 (0.22,0.81) | 0.41 (0.22,0.77) | 0.39 (0.13,1.18) | 0.36 (0.20,0.64) | 0.30 (0.11,0.78) | 0.25 (0.12,0.54) | 0.24 (0.13,0.46) |
| 0.48 (0.31,0.76) | 0.47 (0.30,0.71) | 0.44 (0.16,1.21) | 0.40 (0.29,0.57) | 0.34 (0.15,0.79) | 0.29 (0.16,0.52) | 0.28 (0.18,0.43) |
| 0.63 (0.42,0.93) | 0.60 (0.42,0.88) | 0.57 (0.21,1.54) | 0.52 (0.40,0.69) | 0.44 (0.19,1.00) | 0.37 (0.21,0.65) | 0.36 (0.25,0.53) |
| 0.70 (0.47,1.04) | 0.68 (0.47,0.98) | 0.64 (0.24,1.73) | 0.59 (0.45,0.77) | 0.49 (0.22,1.12) | 0.42 (0.24,0.73) | 0.40 (0.28,0.59) |
| 0.74 (0.48,1.15) | 0.72 (0.47,1.09) | 0.68 (0.25,1.86) | 0.62 (0.44,0.87) | 0.52 (0.22,1.21) | 0.44 (0.25,0.79) | 0.43 (0.28,0.65) |
| 0.81 (0.42,1.57) | 0.78 (0.41,1.50) | 0.74 (0.24,2.28) | 0.68 (0.37,1.24) | 0.57 (0.21,1.51) | 0.48 (0.22,1.04) | 0.47 (0.24,0.90) |
| 0.82 (0.41,1.62) | 0.79 (0.40,1.54) | 0.74 (0.24,2.32) | 0.68 (0.36,1.28) | 0.57 (0.21,1.54) | 0.48 (0.22,1.07) | 0.47 (0.24,0.93) |
| AtezoBev | 0.96 (0.66,1.40) | 0.91 (0.34,2.46) | 0.84 (0.63,1.11) | 0.70 (0.31,1.59) | 0.59 (0.34,1.04) | 0.57 (0.39,0.85) |
| 1.04 (0.71,1.51) | Nivolpi | 0.94 (0.35,2.53) | 0.87 (0.68,1.11) | 0.73 (0.32,1.64) | 0.62 (0.36,1.06) | 0.60 (0.42,0.86) |
| 1.10 (0.41,2.98) | 1.06 (0.40,2.84) | PazoEvero | 0.92 (0.35,2.39) | 0.77 (0.23,2.63) | 0.65 (0.22,1.91) | 0.63 (0.25,1.58) |
| 1.20 (0.90,1.58) | 1.15 (0.90,1.48) | 1.09 (0.42,2.83) | Suni | 0.84 (0.39,1.81) | 1.41 (0.87,2.27) | 0.69 (0.53,0.90) |
| 1.43 (0.63,3.24) | 1.37 (0.61,3.09) | 1.30 (0.38,4.42) | 1.19 (0.55,2.58) | Sora | 0.85 (0.34,2.10) | 0.82 (0.36,1.85) |
| 1.68 (0.96,2.93) | 1.62 (0.94,2.78) | 1.53 (0.52,4.45) | 0.71 (0.44,1.15) | 1.18 (0.48,2.92) | IMA901Suni | 0.97 (0.56,1.67) |
| 1.74 (1.18,2.56) | 1.68 (1.17,2.41) | 1.58 (0.63,3.96) | 1.45 (1.12,1.89) | 1.22 (0.54,2.75) | 1.03 (0.60,1.79) | Pazo |

Abbreviations: Suni: Sunitinib; Cabo: Cabozantinib; Pazo: Pazopanib; Sora: Sorafenib; Axi: Axitinib; Nivo: Nivolumab; Atezo: Atezolizumab; PemAxi: Pembrolizumab plus Axitinib; AtezoBev: Atezolizumab plus Bevacizumab; AveAxi: Avelumab plus Axitinib; LenPem: Lenvatinib plus Pembrolizumab; NivoCabo: Nivolumab plus Cabozantinib; NivoIpi: Nivolumab plus lpilimumab; LenEvero: Lenvatinib plus Everolimus; PazoEvero: Pazopanib plus Everolimus; IMA901Suni: IMA901 plus Sunitinib. Yellow suggests a relative treatment benefit, blue suggests a relative treatment harm, dark red suggests not significant.

Supplementary Table 2F. Matrix of pairwise comparisons of regimens on 30 month progression free survival (shown as odds ratio and 95% confidence intervals).

| LenPem | 0.95 (0.55,1.64) | 0.70 (0.34,1.44) | 0.66 (0.39,1.11) | 0.55 (0.35,0.89) | 0.48 (0.23,1.01) | 0.46 (0.30,0.73) | 0.31 (0.21,0.44) | 0.29 (0.14,0.60) | 0.25 (0.09,0.72) | 0.28 (0.17,0.45) | 0.26 (0.12,0.53) |
| --- | --- | --- | --- | --- | --- | --- | --- | --- | --- | --- | --- |
| 1.05 (0.61,1.81) | NivoCabo | 0.74 (0.35,1.55) | 0.69 (0.40,1.20) | 0.58 (0.35,0.96) | 0.51 (0.24,1.08) | 0.49 (0.30,0.79) | 0.32 (0.22,0.48) | 0.31 (0.15,0.65) | 0.27 (0.09,0.77) | 0.29 (0.17,0.49) | 0.27 (0.13,0.57) |
| 1.43 (0.69,2.96) | 1.36 (0.65,2.86) | Axi | 0.94 (0.45,1.96) | 0.79 (0.40,1.58) | 0.69 (0.28,1.69) | 0.66 (0.34,1.31) | 0.44 (0.23,0.82) | 0.42 (0.17,1.01) | 0.36 (0.11,1.16) | 0.40 (0.20,0.80) | 0.37 (0.15,0.89) |
| 1.52 (0.90,2.58) | 1.44 (0.83,2.51) | 1.06 (0.51,2.21) | LenEvero | 0.84 (0.52,1.36) | 0.73 (0.35,1.54) | 0.71 (0.45,1.12) | 0.47 (0.32,0.68) | 0.44 (0.21,0.92) | 0.38 (0.13,1.10) | 0.42 (0.26,0.69) | 0.39 (0.19,0.81) |
| 1.81 (1.13,2.90) | 1.72 (1.05,2.82) | 1.26 (0.63,2.52) | 1.19 (0.74,1.92) | PemAxi | 0.87 (0.43,1.76) | 0.84 (0.57,1.24) | 0.55 (0.41,0.74) | 0.53 (0.26,1.05) | 0.46 (0.16,1.27) | 0.50 (0.33,0.77) | 0.46 (0.23,0.93) |
| 2.07 (0.99,4.34) | 1.97 (0.93,4.19) | 1.45 (0.59,3.55) | 1.36 (0.65,2.87) | 1.15 (0.57,2.32) | Cabo | 0.96 (0.48,1.92) | 0.64 (0.34,1.21) | 0.60 (0.25,1.48) | 0.52 (0.16,1.69) | 0.57 (0.28,1.17) | 0.53 (0.21,1.30) |
| 2.15 (1.37,3.38) | 2.05 (1.27,3.30) | 1.51 (0.76,2.97) | 1.42 (0.90,2.24) | 1.19 (0.81,1.76) | 1.04 (0.52,2.07) | Nivolpi | 0.66 (0.51,0.86) | 0.63 (0.32,1.24) | 0.55 (0.20,1.50) | 0.60 (0.40,0.90) | 0.55 (0.28,1.09) |
| 3.26 (2.26,4.71) | 3.10 (2.08,4.62) | 2.28 (1.22,4.26) | 2.14 (1.47,3.13) | 1.80 (1.34,2.42) | 1.57 (0.83,2.98) | 1.51 (1.17,1.96) | Suni | 0.95 (0.51,1.78) | 0.82 (0.31,2.20) | 0.90 (0.66,1.24) | 0.83 (0.44,1.57) |
| 3.43 (1.66,7.10) | 3.26 (1.55,6.86) | 2.40 (0.99,5.82) | 2.26 (1.09,4.70) | 1.90 (0.95,3.79) | 1.66 (0.68,4.06) | 1.59 (0.81,3.14) | 1.05 (0.56,1.97) | AtezoBev | 0.87 (0.27,2.78) | 0.95 (0.47,1.92) | 0.88 (0.36,2.14) |
| 3.95 (1.39,11.26) | 3.76 (1.30,10.83) | 2.76 (0.86,8.84) | 2.60 (0.91,7.43) | 2.19 (0.79,6.08) | 1.91 (0.59,6.15) | 1.83 (0.67,5.06) | 1.21 (0.45,3.23) | 1.15 (0.36,3.69) | Sora | 1.10 (0.39,3.07) | 1.01 (0.31,3.24) |
| 3.61 (2.22,5.87) | 3.43 (2.05,5.72) | 2.52 (1.25,5.09) | 2.37 (1.45,3.89) | 1.99 (1.29,3.08) | 1.74 (0.85,3.56) | 1.67 (1.11,2.53) | 1.11 (0.80,1.52) | 1.05 (0.52,2.12) | 0.91 (0.33,2.56) | Pazo | 0.92 (0.45,1.87) |
| 3.92 (1.88,8.16) | 3.72 (1.76,7.88) | 2.74 (1.12,6.68) | 2.58 (1.23,5.39) | 2.17 (1.08,4.36) | 1.89 (0.77,4.65) | 1.82 (0.92,3.61) | 1.20 (0.64,2.27) | 1.14 (0.47,2.78) | 0.99 (0.31,3.19) | 1.09 (0.53,2.21) | Atezo |

Abbreviations: Suni: Sunitinib; Cabo: Cabozantinib; Pazo: Pazopanib; Sora: Sorafenib; Axi: Axitinib; Nivo: Nivolumab; Atezo: Atezolizumab; PemAxi: Pembrolizumab plus Axitinib; AtezoBev: Atezolizumab plus Bevacizumab; LenPem: Lenvatinib plus Pembrolizumab; NivoCabo: Nivolumab plus Cabozantinib; NivoIpi: Nivolumab plus lpilimumab; LenEvero: Lenvatinib plus Everolimus. Yellow suggests a relative treatment benefit, blue suggests a relative treatment harm, dark red suggests not significant.

Supplementary Figure 3. Forst plolt of pairwise comparisons of regimens on 36 month progression free survival. OR (95% CI): odds ratio (95% confidence intervals).

Supplementary Table 3A. Matrix of pairwise comparisons of regimens on 3 month overall survival (shown as odds ratio and 95% confidence intervals).

| Bev | 0.10 (0.00,9.90) | 0.08 (0.00,6.95) | 0.13 (0.01,2.62) | 0.08 (0.00,8.46) | 0.07 (0.00,11.46) | 0.06 (0.00,4.89) | 0.08 (0.00,1.53) | 0.05 (0.00,4.13) |
| --- | --- | --- | --- | --- | --- | --- | --- | --- |
| 9.56 (0.10,904.96) | IMA901Suni | 0.76 (0.18,3.27) | 1.26 (0.04,39.01) | 0.74 (0.09,5.77) | 0.72 (0.05,11.03) | 0.55 (0.14,2.21) | 0.78 (0.02,25.38) | 0.47 (0.12,1.79) |
| 12.52 (0.14,1089.57) | 1.31 (0.31,5.61) | PemAxi | 1.65 (0.06,45.66) | 0.97 (0.15,6.23) | 0.94 (0.07,12.53) | 0.72 (0.24,2.14) | 1.02 (0.04,29.76) | 0.62 (0.22,1.70) |
| 7.57 (0.38,150.45) | 0.79 (0.03,24.46) | 0.60 (0.02,16.71) | Sora | 0.58 (0.02,21.88) | 0.57 (0.01,32.44) | 0.43 (0.02,11.67) | 0.62 (0.34,1.12) | 0.37 (0.01,9.78) |
| 12.95 (0.12,1419.02) | 1.35 (0.17,10.59) | 1.03 (0.16,6.67) | 1.71 (0.05,64.02) | Cabo | 0.97 (0.05,18.94) | 0.74 (0.12,4.56) | 1.06 (0.03,41.54) | 0.64 (0.11,3.74) |
| 13.34 (0.09,2040.41) | 1.40 (0.09,21.47) | 1.07 (0.08,14.23) | 1.76 (0.03,100.68) | 1.03 (0.05,20.10) | Savo | 0.76 (0.06,9.86) | 1.09 (0.02,65.01) | 0.66 (0.05,8.21) |
| 17.47 (0.20,1492.19) | 1.83 (0.45,7.38) | 1.40 (0.47,4.16) | 2.31 (0.09,62.12) | 1.35 (0.22,8.31) | 1.31 (0.10,16.93) | NivoCabo | 1.43 (0.05,40.50) | 0.86 (0.34,2.17) |
| 12.24 (0.65,229.29) | 1.28 (0.04,41.58) | 0.98 (0.03,28.44) | 1.62 (0.90,2.92) | 0.95 (0.02,37.10) | 0.92 (0.02,54.72) | 0.70 (0.02,19.87) | Placebo | 0.60 (0.02,16.67) |
| 20.30 (0.24,1700.42) | 2.12 (0.56,8.05) | 1.62 (0.59,4.46) | 2.68 (0.10,70.31) | 1.57 (0.27,9.20) | 1.52 (0.12,19.01) | 1.16 (0.46,2.93) | 1.66 (0.06,45.86) | Nivolpi |
| 20.61 (0.24,1745.97) | 2.15 (0.55,8.49) | 1.65 (0.57,4.75) | 2.72 (0.10,72.49) | 1.59 (0.26,9.61) | 1.54 (0.12,19.69) | 1.18 (0.44,3.13) | 1.68 (0.06,47.27) | 1.01 (0.42,2.46) |
| 22.17 (0.24,2029.35) | 2.32 (0.47,11.53) | 1.77 (0.46,6.82) | 2.93 (0.10,86.52) | 1.71 (0.24,12.42) | 1.66 (0.11,24.19) | 1.27 (0.35,4.58) | 1.81 (0.06,56.33) | 1.09 (0.32,3.68) |
| 23.84 (0.27,2138.89) | 2.49 (0.53,11.70) | 1.90 (0.53,6.84) | 3.15 (0.11,90.58) | 1.84 (0.27,12.75) | 1.79 (0.13,25.14) | 1.36 (0.41,4.58) | 1.95 (0.06,59.00) | 1.17 (0.38,3.67) |
| 25.32 (0.27,2385.77) | 2.65 (0.49,14.25) | 2.02 (0.48,8.55) | 0.25 (0.01,7.82) | 1.96 (0.25,15.13) | 1.90 (0.12,28.99) | 1.45 (0.36,5.77) | 2.07 (0.06,66.82) | 1.25 (0.33,4.66) |
| 28.18 (0.34,2355.64) | 2.95 (0.78,11.10) | 2.25 (0.83,6.13) | 3.72 (0.14,97.33) | 2.18 (0.37,12.71) | 2.11 (0.17,26.30) | 1.61 (0.65,4.02) | 2.30 (0.08,63.49) | 1.39 (0.62,3.13) |
| 30.65 (0.35,2712.91) | 3.21 (0.71,14.46) | 2.45 (0.72,8.38) | 4.05 (0.14,114.36) | 2.37 (0.35,15.88) | 2.30 (0.17,31.58) | 1.75 (0.55,5.59) | 2.50 (0.08,74.51) | 1.51 (0.51,4.46) |
| 34.15 (0.42,2752.10) | 3.57 (1.08,11.84) | 2.73 (1.19,6.23) | 4.51 (0.18,112.23) | 2.64 (0.50,14.02) | 2.56 (0.22,29.87) | 1.95 (0.96,4.00) | 2.79 (0.11,73.26) | 1.68 (0.94,3.02) |
| 50.02 (0.57,4381.95) | 5.23 (1.20,22.88) | 4.00 (1.21,13.16) | 6.61 (0.24,184.06) | 3.86 (0.59,25.29) | 3.75 (0.28,50.64) | 2.86 (0.93,8.76) | 4.09 (0.14,119.94) | 2.46 (0.87,6.97) |

Supplementary Table 3A (continued)

| 0.05 (0.00,4.11) | 0.05 (0.00,4.13) | 0.04 (0.00,3.76) | 0.04 (0.00,3.72) | 0.04 (0.00,2.97) | 0.03 (0.00,2.89) | 0.03 (0.00,2.36) | 0.02 (0.00,1.75) |
| --- | --- | --- | --- | --- | --- | --- | --- |
| 0.46 (0.12,1.83) | 0.43 (0.09,2.14) | 0.40 (0.09,1.88) | 0.38 (0.07,2.03) | 0.34 (0.09,1.28) | 0.31 (0.07,1.41) | 0.28 (0.08,0.93) | 0.19 (0.04,0.84) |
| 0.61 (0.21,1.75) | 0.56 (0.15,2.17) | 0.53 (0.15,1.89) | 0.49 (0.12,2.09) | 0.44 (0.16,1.21) | 0.41 (0.12,1.40) | 0.37 (0.16,0.84) | 0.25 (0.08,0.82) |
| 0.37 (0.01,9.79) | 0.34 (0.01,10.09) | 0.32 (0.01,9.14) | 3.94 (0.13,121.18) | 0.27 (0.01,7.03) | 0.25 (0.01,6.98) | 0.22 (0.01,5.52) | 0.15 (0.01,4.22) |
| 0.63 (0.10,3.80) | 0.58 (0.08,4.24) | 0.54 (0.08,3.76) | 0.51 (0.07,3.96) | 0.46 (0.08,2.68) | 0.42 (0.06,2.84) | 0.38 (0.07,2.02) | 0.26 (0.04,1.70) |
| 0.65 (0.05,8.25) | 0.60 (0.04,8.76) | 0.56 (0.04,7.87) | 0.53 (0.03,8.05) | 0.47 (0.04,5.89) | 0.44 (0.03,5.98) | 0.39 (0.03,4.56) | 0.27 (0.02,3.60) |
| 0.85 (0.32,2.25) | 0.79 (0.22,2.85) | 0.73 (0.22,2.46) | 0.69 (0.17,2.75) | 0.62 (0.25,1.55) | 0.57 (0.18,1.82) | 0.51 (0.25,1.05) | 0.35 (0.11,1.07) |
| 0.59 (0.02,16.68) | 0.55 (0.02,17.17) | 0.51 (0.02,15.55) | 0.48 (0.01,15.61) | 0.43 (0.02,11.97) | 0.40 (0.01,11.88) | 0.36 (0.01,9.41) | 0.24 (0.01,7.18) |
| 0.99 (0.41,2.39) | 0.92 (0.27,3.09) | 0.85 (0.27,2.66) | 0.80 (0.21,3.00) | 0.72 (0.32,1.63) | 0.66 (0.22,1.96) | 0.59 (0.33,1.07) | 0.41 (0.14,1.15) |
| Pazo | 0.93 (0.26,3.27) | 0.86 (0.26,2.82) | 0.81 (0.21,3.16) | 0.73 (0.30,1.75) | 0.67 (0.22,2.08) | 0.60 (0.31,1.17) | 0.41 (0.14,1.22) |
| 1.08 (0.31,3.78) | Axi | 0.93 (0.22,3.95) | 0.88 (0.18,4.30) | 0.79 (0.24,2.63) | 0.72 (0.18,2.94) | 0.65 (0.22,1.88) | 0.44 (0.11,1.74) |
| 1.16 (0.35,3.77) | 1.08 (0.25,4.56) | LenPem | 0.94 (0.20,4.36) | 0.85 (0.27,2.62) | 0.78 (0.20,2.96) | 0.70 (0.26,1.85) | 0.48 (0.13,1.75) |
| 1.23 (0.32,4.77) | 1.14 (0.23,5.61) | 1.06 (0.23,4.92) | Anlo | 0.90 (0.24,3.33) | 0.83 (0.19,3.68) | 3.34 (0.11,102.69) | 0.51 (0.12,2.18) |
| 1.37 (0.57,3.28) | 1.27 (0.38,4.25) | 1.18 (0.38,3.66) | 1.11 (0.30,4.13) | AtezoBev | 0.92 (0.31,2.69) | 0.83 (0.47,1.46) | 0.56 (0.20,1.58) |
| 1.49 (0.48,4.60) | 1.38 (0.34,5.62) | 1.29 (0.34,4.90) | 1.21 (0.27,5.39) | 1.09 (0.37,3.19) | LenEvero | 0.90 (0.36,2.24) | 0.61 (0.17,2.15) |
| 1.66 (0.85,3.23) | 1.54 (0.53,4.47) | 1.43 (0.54,3.81) | 0.30 (0.01,9.18) | 1.21 (0.69,2.14) | 1.11 (0.45,2.78) | Suni | 0.68 (0.29,1.61) |
| 2.43 (0.82,7.21) | 2.26 (0.57,8.87) | 2.10 (0.57,7.71) | 1.98 (0.46,8.52) | 1.77 (0.63,4.97) | 1.63 (0.47,5.72) | 1.46 (0.62,3.46) | AveAxi |

Abbreviations: Suni: Sunitinib; Bev: Bevacizumab; Cabo: Cabozantinib; Pazo: Pazopanib; Savo: Savolitinib; Sora: Sorafenib; Axi: Axitinib; Anlo: Anlotinib; PemAxi: Pembrolizumab plus Axitinib; AtezoBev: Atezolizumab plus Bevacizumab; AveAxi: Avelumab plus Axitinib; LenPem: Lenvatinib plus Pembrolizumab; NivoCabo: Nivolumab plus Cabozantinib; NivoIpi: Nivolumab plus lpilimumab; LenEvero: Lenvatinib plus Everolimus; IMA901Suni: IMA901 plus Sunitinib.

Yellow suggests a relative treatment benefit, blue suggests a relative treatment harm, dark red suggests not significant.

Supplementary Table 3B. Matrix of pairwise comparisons of regimens on 6 month overall survival (shown as odds ratio and 95% confidence intervals).

| LenPem | 1.15 (0.18,7.55) | 0.81 (0.33,2.00) | 0.68 (0.28,1.68) | 0.70 (0.26,1.91) | 0.67 (0.20,2.29) | 0.56 (0.23,1.39) | 0.43 (0.19,1.00) | 0.40 (0.14,1.19) |
| --- | --- | --- | --- | --- | --- | --- | --- | --- |
| 0.87 (0.13,5.66) | Savo | 0.70 (0.11,4.27) | 0.59 (0.10,3.59) | 0.60 (0.09,3.88) | 0.58 (0.08,4.26) | 0.48 (0.08,2.96) | 0.38 (0.06,2.21) | 0.35 (0.05,2.34) |
| 1.24 (0.50,3.06) | 1.43 (0.23,8.72) | PemAxi | 0.84 (0.40,1.78) | 0.86 (0.36,2.06) | 0.83 (0.27,2.54) | 0.69 (0.33,1.47) | 0.54 (0.28,1.04) | 0.50 (0.19,1.29) |
| 1.47 (0.60,3.63) | 1.70 (0.28,10.35) | 1.19 (0.56,2.51) | NivoCabo | 1.03 (0.43,2.44) | 0.99 (0.33,3.01) | 0.82 (0.39,1.74) | 0.64 (0.33,1.24) | 0.59 (0.23,1.54) |
| 1.43 (0.52,3.91) | 1.65 (0.26,10.62) | 1.16 (0.49,2.76) | 0.97 (0.41,2.32) | Axi | 0.97 (0.29,3.19) | 0.80 (0.34,1.91) | 0.62 (0.28,1.37) | 0.58 (0.20,1.65) |
| 1.48 (0.44,5.04) | 1.71 (0.24,12.48) | 1.20 (0.39,3.65) | 1.01 (0.33,3.06) | 1.04 (0.31,3.42) | Cabo | 0.83 (0.27,2.53) | 0.64 (0.22,1.85) | 0.60 (0.17,2.10) |
| 1.79 (0.72,4.44) | 2.07 (0.34,12.62) | 1.45 (0.68,3.07) | 1.22 (0.57,2.58) | 1.25 (0.52,2.98) | 1.21 (0.40,3.68) | AveAxi | 0.77 (0.40,1.51) | 0.72 (0.28,1.88) |
| 2.31 (1.00,5.33) | 2.67 (0.45,15.72) | 1.87 (0.96,3.63) | 1.57 (0.81,3.05) | 1.61 (0.73,3.57) | 1.56 (0.54,4.48) | 1.29 (0.66,2.52) | Nivolpi | 0.93 (0.38,2.26) |
| 2.48 (0.84,7.31) | 2.87 (0.43,19.19) | 2.01 (0.77,5.21) | 1.69 (0.65,4.37) | 1.73 (0.61,4.95) | 1.67 (0.48,5.89) | 1.39 (0.53,3.61) | 1.07 (0.44,2.61) | IMA901Suni |
| 2.69 (0.86,8.41) | 3.11 (0.45,21.54) | 2.18 (0.79,6.04) | 1.83 (0.66,5.07) | 1.88 (0.62,5.70) | 1.82 (0.49,6.73) | 1.51 (0.54,4.18) | 1.17 (0.45,3.04) | 1.09 (0.33,3.52) |
| 2.64 (1.14,6.08) | 3.05 (0.52,17.94) | 2.13 (1.10,4.14) | 1.79 (0.93,3.47) | 1.84 (0.83,4.07) | 1.78 (0.62,5.11) | 1.47 (0.76,2.87) | 1.14 (0.65,2.01) | 1.06 (0.44,2.58) |
| 2.85 (1.26,6.46) | 3.29 (0.56,19.25) | 2.30 (1.21,4.38) | 1.94 (1.02,3.68) | 1.99 (0.92,4.33) | 1.92 (0.68,5.45) | 1.59 (0.83,3.04) | 1.23 (0.72,2.12) | 1.15 (0.48,2.74) |
| 3.17 (1.28,7.84) | 3.66 (0.60,22.31) | 2.56 (1.21,5.43) | 2.15 (1.02,4.56) | 2.21 (0.93,5.27) | 2.13 (0.70,6.50) | 1.77 (0.83,3.77) | 1.37 (0.70,2.67) | 1.28 (0.49,3.31) |
| 3.05 (1.46,6.36) | 3.52 (0.63,19.84) | 2.47 (1.45,4.19) | 2.07 (1.22,3.52) | 2.13 (1.07,4.23) | 2.06 (0.77,5.47) | 1.71 (1.00,2.91) | 1.32 (0.88,1.97) | 1.23 (0.56,2.71) |
| 8.51 (0.65,111.10) | 9.82 (0.49,199.00) | 6.88 (0.55,85.38) | 5.78 (0.47,71.77) | 5.94 (0.46,76.58) | 5.74 (0.41,81.16) | 4.76 (0.38,59.09) | 3.68 (0.30,44.66) | 3.43 (0.26,45.55) |
| 12.85 (1.34,123.19) | 14.84 (0.95,231.92) | 10.38 (1.15,94.01) | 8.73 (0.97,79.01) | 8.97 (0.95,84.76) | 8.66 (0.83,90.95) | 7.18 (0.79,65.07) | 5.56 (0.63,49.00) | 5.18 (0.53,50.62) |
| 21.94 (2.22,216.46) | 25.34 (1.58,405.48) | 17.74 (1.90,165.30) | 14.92 (1.60,138.94) | 15.33 (1.58,148.95) | 14.80 (1.37,159.64) | 12.27 (1.31,114.42) | 9.50 (1.05,86.19) | 8.84 (0.88,88.93) |

Supplementary Table 3B (continued)

| 0.37 (0.12,1.16) | 0.38 (0.16,0.87) | 0.35 (0.15,0.80) | 0.32 (0.13,0.78) | 0.33 (0.16,0.68) | 0.12 (0.01,1.54) | 0.08 (0.01,0.75) | 0.05 (0.00,0.45) |
| --- | --- | --- | --- | --- | --- | --- | --- |
| 0.32 (0.05,2.23) | 0.33 (0.06,1.93) | 0.30 (0.05,1.78) | 0.27 (0.04,1.67) | 0.28 (0.05,1.60) | 0.10 (0.01,2.06) | 0.07 (0.00,1.05) | 0.04 (0.00,0.63) |
| 0.46 (0.17,1.27) | 0.47 (0.24,0.91) | 0.43 (0.23,0.83) | 0.39 (0.18,0.83) | 0.41 (0.24,0.69) | 0.15 (0.01,1.81) | 0.10 (0.01,0.87) | 0.06 (0.01,0.53) |
| 0.55 (0.20,1.51) | 0.56 (0.29,1.08) | 0.52 (0.27,0.98) | 0.46 (0.22,0.98) | 0.48 (0.28,0.82) | 0.17 (0.01,2.15) | 0.11 (0.01,1.04) | 0.07 (0.01,0.62) |
| 0.53 (0.18,1.61) | 0.54 (0.25,1.20) | 0.50 (0.23,1.09) | 0.45 (0.19,1.08) | 0.47 (0.24,0.93) | 0.17 (0.01,2.17) | 0.11 (0.01,1.05) | 0.07 (0.01,0.63) |
| 0.55 (0.15,2.04) | 0.56 (0.20,1.62) | 0.52 (0.18,1.48) | 0.47 (0.15,1.43) | 0.49 (0.18,1.29) | 0.17 (0.01,2.47) | 0.12 (0.01,1.21) | 0.07 (0.01,0.73) |
| 0.66 (0.24,1.84) | 0.68 (0.35,1.32) | 0.63 (0.33,1.20) | 0.57 (0.27,1.20) | 0.59 (0.34,1.00) | 0.21 (0.02,2.61) | 0.14 (0.02,1.26) | 0.08 (0.01,0.76) |
| 0.86 (0.33,2.23) | 0.88 (0.50,1.54) | 0.81 (0.47,1.39) | 0.73 (0.37,1.42) | 0.76 (0.51,1.13) | 0.27 (0.02,3.29) | 0.18 (0.02,1.58) | 0.11 (0.01,0.95) |
| 0.92 (0.28,2.99) | 0.94 (0.39,2.28) | 0.87 (0.36,2.08) | 0.78 (0.30,2.04) | 0.81 (0.37,1.80) | 0.29 (0.02,3.88) | 0.19 (0.02,1.89) | 0.11 (0.01,1.14) |
| Anlo | 1.02 (0.39,2.66) | 0.95 (0.37,2.42) | 0.85 (0.31,2.36) | 0.21 (0.02,2.11) | 0.32 (0.02,4.31) | 8.94 (0.92,86.79) | 0.12 (0.01,1.27) |
| 0.98 (0.38,2.55) | AtezoBev | 0.93 (0.54,1.58) | 0.83 (0.43,1.62) | 0.86 (0.58,1.28) | 0.31 (0.03,3.75) | 0.21 (0.02,1.81) | 0.12 (0.01,1.09) |
| 1.06 (0.41,2.71) | 1.08 (0.63,1.85) | Pazo | 0.90 (0.47,1.71) | 0.93 (0.65,1.34) | 0.34 (0.03,4.04) | 0.22 (0.03,1.94) | 0.13 (0.01,1.17) |
| 1.18 (0.42,3.26) | 1.20 (0.62,2.33) | 1.11 (0.58,2.11) | LenEvero | 1.04 (0.61,1.77) | 0.37 (0.03,4.62) | 0.25 (0.03,2.23) | 0.14 (0.02,1.34) |
| 4.77 (0.47,47.96) | 1.16 (0.78,1.72) | 1.07 (0.75,1.54) | 0.96 (0.57,1.64) | Suni | 0.36 (0.03,4.21) | 0.24 (0.03,2.01) | 0.14 (0.02,1.22) |
| 3.16 (0.23,43.01) | 3.23 (0.27,39.07) | 2.98 (0.25,35.95) | 2.69 (0.22,33.38) | 2.79 (0.24,32.72) | Bev | 0.66 (0.20,2.25) | 0.39 (0.12,1.25) |
| 0.11 (0.01,1.09) | 4.87 (0.55,42.86) | 4.51 (0.52,39.41) | 4.06 (0.45,36.76) | 4.21 (0.50,35.73) | 1.51 (0.45,5.12) | Sora | 0.59 (0.41,0.84) |
| 8.15 (0.79,84.23) | 8.32 (0.92,75.39) | 7.70 (0.85,69.33) | 6.93 (0.74,64.63) | 7.19 (0.82,62.87) | 2.58 (0.80,8.29) | 1.71 (1.19,2.45) | Placebo |

Abbreviations: Suni: Sunitinib; Bev: Bevacizumab; Cabo: Cabozantinib; Pazo: Pazopanib; Savo: Savolitinib; Sora: Sorafenib; Axi: Axitinib; Anlo: Anlotinib; PemAxi: Pembrolizumab plus Axitinib; AtezoBev: Atezolizumab plus Bevacizumab; AveAxi: Avelumab plus Axitinib; LenPem: Lenvatinib plus Pembrolizumab; NivoCabo: Nivolumab plus Cabozantinib; NivoIpi: Nivolumab plus lpilimumab; LenEvero: Lenvatinib plus Everolimus; IMA901Suni: IMA901 plus Sunitinib.

Yellow suggests a relative treatment benefit, blue suggests a relative treatment harm, dark red suggests not significant.

Supplementary Table 3C. Matrix of pairwise comparisons of regimens on 12 month overall survival (shown as odds ratio and 95% confidence intervals).

| Lenpem | 0.90 (0.50,1.63) | 0.81 (0.26,2.56) | 0.70 (0.38,1.28) | 0.63 (0.26,1.51) | 0.59 (0.29,1.19) | 0.56 (0.33,0.96) | 0.52 (0.29,0.93) | 0.53 (0.23,1.23) |
| --- | --- | --- | --- | --- | --- | --- | --- | --- |
| 1.11 (0.61,2.02) | PemAxi | 0.90 (0.29,2.78) | 0.78 (0.45,1.36) | 0.70 (0.30,1.63) | 0.66 (0.34,1.28) | 0.62 (0.38,1.02) | 0.58 (0.34,0.98) | 0.59 (0.26,1.32) |
| 1.24 (0.39,3.91) | 1.11 (0.36,3.43) | Savo | 0.87 (0.28,2.68) | 0.78 (0.21,2.85) | 0.73 (0.22,2.40) | 0.69 (0.23,2.08) | 0.64 (0.21,1.97) | 0.66 (0.18,2.35) |
| 1.43 (0.78,2.60) | 1.28 (0.74,2.24) | 1.16 (0.37,3.58) | NivoCabo | 0.90 (0.39,2.10) | 0.85 (0.43,1.65) | 0.80 (0.49,1.32) | 0.74 (0.43,1.27) | 0.76 (0.34,1.71) |
| 1.59 (0.66,3.79) | 1.43 (0.61,3.31) | 1.28 (0.35,4.69) | 1.11 (0.48,2.59) | Anlo | 0.94 (0.37,2.36) | 0.89 (0.40,1.98) | 0.82 (0.36,1.89) | 0.84 (0.30,2.35) |
| 1.69 (0.84,3.41) | 1.52 (0.78,2.95) | 1.37 (0.42,4.48) | 1.18 (0.61,2.31) | 1.06 (0.42,2.67) | Axi | 0.95 (0.51,1.75) | 0.88 (0.46,1.68) | 0.90 (0.37,2.18) |
| 1.79 (1.04,3.07) | 1.61 (0.98,2.62) | 1.44 (0.48,4.34) | 1.25 (0.76,2.06) | 1.13 (0.50,2.52) | 1.06 (0.57,1.96) | Nivolpi | 0.93 (0.58,1.49) | 0.95 (0.44,2.04) |
| 1.92 (1.08,3.43) | 1.73 (1.02,2.94) | 1.56 (0.51,4.76) | 1.35 (0.79,2.31) | 1.21 (0.53,2.78) | 1.14 (0.60,2.18) | 1.08 (0.67,1.72) | AveAxi | 1.02 (0.46,2.26) |
| 1.88 (0.81,4.34) | 1.69 (0.76,3.78) | 1.52 (0.43,5.43) | 1.32 (0.59,2.96) | 1.19 (0.42,3.31) | 1.11 (0.46,2.70) | 1.05 (0.49,2.27) | 0.98 (0.44,2.16) | Cabo |
| 2.21 (1.32,3.69) | 1.98 (1.25,3.15) | 1.78 (0.60,5.30) | 1.54 (0.97,2.47) | 1.39 (0.63,3.06) | 1.31 (0.72,2.36) | 1.23 (0.84,1.83) | 1.15 (0.74,1.78) | 1.17 (0.55,2.48) |
| 2.42 (1.40,4.20) | 2.18 (1.32,3.60) | 1.96 (0.65,5.92) | 1.70 (1.02,2.82) | 1.53 (0.68,3.44) | 1.43 (0.77,2.68) | 1.36 (0.88,2.10) | 1.26 (0.78,2.04) | 1.29 (0.59,2.79) |
| 2.59 (1.45,4.64) | 2.33 (1.37,3.98) | 2.10 (0.68,6.44) | 1.82 (1.06,3.12) | 1.64 (0.71,3.76) | 1.54 (0.80,2.95) | 1.45 (0.90,2.33) | 1.35 (0.81,2.26) | 1.38 (0.62,3.05) |
| 3.14 (0.89,11.14) | 2.82 (0.81,9.81) | 2.54 (0.52,12.43) | 2.20 (0.63,7.66) | 1.98 (0.49,8.02) | 1.86 (0.51,6.82) | 1.76 (0.52,5.96) | 1.63 (0.47,5.63) | 1.67 (0.42,6.62) |
| 2.64 (1.68,4.14) | 2.37 (1.61,3.50) | 2.14 (0.74,6.16) | 1.85 (1.24,2.75) | 2.46 (0.88,6.86) | 1.56 (0.91,2.68) | 1.48 (1.10,2.00) | 1.37 (0.96,1.97) | 1.40 (0.69,2.84) |
| 3.89 (1.69,8.99) | 3.50 (1.57,7.83) | 3.15 (0.88,11.24) | 2.73 (1.21,6.12) | 0.41 (0.16,1.04) | 2.31 (0.95,5.60) | 2.18 (1.01,4.69) | 2.02 (0.92,4.47) | 2.07 (0.76,5.61) |
| 3.74 (1.83,7.65) | 3.37 (1.71,6.63) | 3.03 (0.92,10.01) | 2.62 (1.32,5.19) | 2.36 (0.93,5.99) | 2.22 (1.02,4.80) | 2.10 (1.11,3.94) | 1.95 (1.00,3.78) | 1.99 (0.81,4.88) |
| 5.68 (2.36,13.70) | 5.11 (2.19,11.95) | 4.60 (1.25,16.88) | 3.98 (1.69,9.34) | 3.58 (1.24,10.37) | 3.37 (1.33,8.51) | 3.18 (1.41,7.17) | 2.95 (1.28,6.83) | 3.02 (1.07,8.49) |

Supplementary Table 3C (continued)

| 0.45 (0.27,0.76) | 0.41 (0.24,0.72) | 0.39 (0.22,0.69) | 0.32 (0.09,1.13) | 0.38 (0.24,0.59) | 0.26 (0.11,0.59) | 0.27 (0.13,0.55) | 0.18 (0.07,0.42) |
| --- | --- | --- | --- | --- | --- | --- | --- |
| 0.50 (0.32,0.80) | 0.46 (0.28,0.76) | 0.43 (0.25,0.73) | 0.35 (0.10,1.23) | 0.42 (0.29,0.62) | 0.29 (0.13,0.64) | 0.30 (0.15,0.59) | 0.20 (0.08,0.46) |
| 0.56 (0.19,1.66) | 0.51 (0.17,1.54) | 0.48 (0.16,1.46) | 0.39 (0.08,1.93) | 0.47 (0.16,1.35) | 0.32 (0.09,1.13) | 0.33 (0.10,1.09) | 0.22 (0.06,0.80) |
| 0.65 (0.41,1.04) | 0.59 (0.35,0.98) | 0.55 (0.32,0.95) | 0.45 (0.13,1.58) | 0.54 (0.36,0.80) | 0.37 (0.16,0.82) | 0.38 (0.19,0.76) | 0.25 (0.11,0.59) |
| 0.72 (0.33,1.58) | 0.65 (0.29,1.47) | 0.61 (0.27,1.40) | 0.50 (0.12,2.05) | 0.41 (0.15,1.14) | 2.43 (0.96,6.16) | 0.42 (0.17,1.07) | 0.28 (0.10,0.81) |
| 0.77 (0.42,1.39) | 0.70 (0.37,1.30) | 0.65 (0.34,1.25) | 0.54 (0.15,1.97) | 0.64 (0.37,1.10) | 0.43 (0.18,1.05) | 0.45 (0.21,0.98) | 0.30 (0.12,0.75) |
| 0.81 (0.55,1.20) | 0.74 (0.48,1.14) | 0.69 (0.43,1.11) | 0.57 (0.17,1.93) | 0.68 (0.50,0.91) | 0.46 (0.21,0.99) | 0.48 (0.25,0.90) | 0.31 (0.14,0.71) |
| 0.87 (0.56,1.36) | 0.79 (0.49,1.29) | 0.74 (0.44,1.24) | 0.61 (0.18,2.11) | 0.73 (0.51,1.05) | 0.49 (0.22,1.09) | 0.51 (0.26,1.00) | 0.34 (0.15,0.78) |
| 0.85 (0.40,1.80) | 0.78 (0.36,1.68) | 0.73 (0.33,1.61) | 0.60 (0.15,2.37) | 0.71 (0.35,1.44) | 0.48 (0.18,1.31) | 0.50 (0.20,1.23) | 0.33 (0.12,0.93) |
| Pazo | 0.91 (0.61,1.37) | 0.85 (0.55,1.33) | 0.70 (0.21,2.35) | 0.84 (0.65,1.07) | 0.57 (0.27,1.20) | 0.59 (0.32,1.08) | 0.39 (0.18,0.86) |
| 1.10 (0.73,1.65) | AtezoBev | 0.93 (0.57,1.52) | 0.77 (0.23,2.63) | 0.92 (0.67,1.26) | 0.62 (0.29,1.35) | 0.65 (0.34,1.23) | 0.43 (0.19,0.97) |
| 1.18 (0.75,1.83) | 1.07 (0.66,1.74) | LenEvero | 0.83 (0.24,2.85) | 0.98 (0.68,1.42) | 0.67 (0.30,1.47) | 0.69 (0.36,1.35) | 0.46 (0.20,1.06) |
| 1.42 (0.42,4.77) | 1.30 (0.38,4.41) | 1.21 (0.35,4.18) | Bev | 1.19 (0.36,3.88) | 0.81 (0.31,2.08) | 0.84 (0.23,3.10) | 0.55 (0.22,1.37) |
| 1.20 (0.93,1.54) | 1.09 (0.79,1.50) | 1.02 (0.71,1.47) | 0.84 (0.26,2.74) | Suni | 0.68 (0.33,1.37) | 0.71 (0.40,1.23) | 0.46 (0.22,0.99) |
| 1.77 (0.84,3.73) | 1.61 (0.74,3.49) | 1.50 (0.68,3.32) | 1.24 (0.48,3.21) | 1.47 (0.73,2.99) | Sora | 1.04 (0.42,2.55) | 0.69 (0.52,0.90) |
| 1.70 (0.92,3.12) | 1.55 (0.81,2.93) | 1.44 (0.74,2.81) | 1.19 (0.32,4.40) | 1.42 (0.81,2.47) | 0.96 (0.39,2.36) | IMA901Suni | 0.66 (0.26,1.68) |
| 2.58 (1.16,5.71) | 2.35 (1.03,5.33) | 2.19 (0.95,5.07) | 1.81 (0.73,4.50) | 2.15 (1.01,4.58) | 1.46 (1.11,1.91) | 1.52 (0.59,3.88) | Placebo |

Abbreviations: Suni: Sunitinib; Bev: Bevacizumab; Cabo: Cabozantinib; Pazo: Pazopanib; Savo: Savolitinib; Sora: Sorafenib; Axi: Axitinib; Anlo: Anlotinib; PemAxi: Pembrolizumab plus Axitinib; AtezoBev: Atezolizumab plus Bevacizumab; AveAxi: Avelumab plus Axitinib; LenPem: Lenvatinib plus Pembrolizumab; NivoCabo: Nivolumab plus Cabozantinib; NivoIpi: Nivolumab plus lpilimumab; LenEvero: Lenvatinib plus Everolimus; IMA901Suni: IMA901 plus Sunitinib.

Yellow suggests a relative treatment benefit, blue suggests a relative treatment harm, dark red suggests not significant.

Supplementary Table 3D. Matrix of pairwise comparisons of regimens on 18 month overall survival (shown as odds ratio and 95% confidence intervals).

| LenPem | 0.72 (0.43,1.22) | 0.72 (0.43,1.19) | 0.67 (0.42,1.08) | 0.71 (0.24,2.13) | 0.62 (0.33,1.19) | 0.60 (0.37,0.99) | 0.50 (0.31,0.82) | 0.48 (0.30,0.75) |
| --- | --- | --- | --- | --- | --- | --- | --- | --- |
| 1.38 (0.82,2.34) | NivoCabo | 0.99 (0.62,1.60) | 0.93 (0.60,1.45) | 0.98 (0.33,2.92) | 0.86 (0.46,1.61) | 0.83 (0.52,1.34) | 0.70 (0.44,1.10) | 0.66 (0.43,1.00) |
| 1.40 (0.84,2.31) | 1.01 (0.63,1.62) | PemAxi | 0.94 (0.62,1.43) | 0.99 (0.34,2.91) | 0.87 (0.48,1.59) | 0.84 (0.54,1.31) | 0.70 (0.46,1.08) | 0.66 (0.45,0.98) |
| 1.48 (0.92,2.38) | 1.07 (0.69,1.67) | 1.06 (0.70,1.61) | Nivolpi | 1.05 (0.36,3.05) | 0.92 (0.52,1.65) | 0.89 (0.59,1.35) | 0.75 (0.51,1.11) | 0.71 (0.50,1.00) |
| 1.41 (0.47,4.23) | 1.02 (0.34,3.02) | 1.01 (0.34,2.96) | 0.95 (0.33,2.74) | Savo | 0.88 (0.28,2.77) | 0.85 (0.29,2.48) | 0.71 (0.24,2.06) | 0.67 (0.23,1.92) |
| 1.61 (0.84,3.06) | 1.16 (0.62,2.16) | 1.15 (0.63,2.10) | 1.08 (0.61,1.93) | 1.14 (0.36,3.60) | Axi | 0.97 (0.53,1.76) | 0.81 (0.45,1.45) | 0.76 (0.44,1.34) |
| 1.66 (1.01,2.73) | 1.20 (0.75,1.92) | 1.19 (0.76,1.85) | 1.12 (0.74,1.68) | 1.18 (0.40,3.45) | 1.03 (0.57,1.88) | AveAxi | 0.84 (0.55,1.27) | 0.79 (0.54,1.16) |
| 1.98 (1.22,3.20) | 1.43 (0.91,2.25) | 1.42 (0.93,2.17) | 1.33 (0.90,1.97) | 1.41 (0.48,4.09) | 1.23 (0.69,2.21) | 1.19 (0.79,1.81) | AtezoBev | 0.94 (0.66,1.35) |
| 2.10 (1.34,3.30) | 1.52 (1.00,2.31) | 1.51 (1.02,2.23) | 1.42 (1.00,2.01) | 1.49 (0.52,4.28) | 1.31 (0.75,2.29) | 1.27 (0.86,1.86) | 1.06 (0.74,1.52) | Pazo |
| 2.22 (0.97,5.06) | 1.60 (0.71,3.60) | 1.59 (0.72,3.52) | 1.49 (0.69,3.24) | 1.57 (0.45,5.55) | 1.38 (0.57,3.36) | 1.34 (0.61,2.95) | 1.12 (0.51,2.44) | 1.05 (0.49,2.26) |
| 2.40 (1.14,5.07) | 1.74 (0.84,3.60) | 1.72 (0.85,3.51) | 1.62 (0.81,3.23) | 1.71 (0.51,5.72) | 1.50 (0.66,3.39) | 1.45 (0.72,2.94) | 1.21 (0.61,2.43) | 1.14 (0.58,2.24) |
| 2.33 (1.58,3.44) | 1.69 (1.18,2.40) | 1.67 (1.22,2.30) | 1.57 (1.20,2.05) | 1.66 (0.59,4.63) | 1.45 (0.87,2.43) | 1.41 (1.03,1.92) | 1.18 (0.89,1.56) | 1.11 (0.89,1.39) |
| 2.70 (1.32,5.52) | 1.95 (0.97,3.92) | 1.93 (0.98,3.82) | 1.82 (0.94,3.51) | 1.92 (0.58,6.31) | 1.68 (0.76,3.70) | 1.63 (0.83,3.20) | 1.36 (0.70,2.65) | 1.28 (0.68,2.44) |
| 2.65 (1.59,4.42) | 1.92 (1.18,3.11) | 1.90 (1.20,3.00) | 1.79 (1.17,2.73) | 1.88 (0.64,5.55) | 1.65 (0.90,3.04) | 1.60 (1.02,2.51) | 1.34 (0.87,2.07) | 1.26 (0.84,1.88) |
| 3.26 (1.73,6.15) | 2.36 (1.28,4.35) | 2.34 (1.29,4.23) | 2.20 (1.24,3.88) | 2.32 (0.74,7.27) | 2.03 (0.99,4.16) | 1.97 (1.09,3.54) | 1.65 (0.93,2.93) | 1.55 (0.89,2.69) |
| 7.16 (2.17,23.63) | 5.17 (1.59,16.88) | 5.13 (1.59,16.57) | 4.82 (1.51,15.38) | 5.09 (1.11,23.41) | 4.46 (1.29,15.40) | 4.32 (1.34,13.91) | 3.62 (1.13,11.57) | 3.40 (1.08,10.76) |
| 8.31 (3.86,17.88) | 6.01 (2.84,12.69) | 5.96 (2.87,12.38) | 5.60 (2.75,11.41) | 5.91 (1.74,20.03) | 5.18 (2.25,11.93) | 5.01 (2.42,10.38) | 4.20 (2.05,8.59) | 3.95 (1.97,7.93) |

Supplementary Table 3D (continued)

| 0.45 (0.20,1.03) | 0.42 (0.20,0.88) | 0.43 (0.29,0.63) | 0.37 (0.18,0.76) | 0.38 (0.23,0.63) | 0.31 (0.16,0.58) | 0.14 (0.04,0.46) | 0.12 (0.06,0.26) |
| --- | --- | --- | --- | --- | --- | --- | --- |
| 0.62 (0.28,1.40) | 0.58 (0.28,1.19) | 0.59 (0.42,0.85) | 0.51 (0.26,1.03) | 0.52 (0.32,0.85) | 0.42 (0.23,0.78) | 0.19 (0.06,0.63) | 0.17 (0.08,0.35) |
| 0.63 (0.28,1.39) | 0.58 (0.29,1.18) | 0.60 (0.44,0.82) | 0.52 (0.26,1.02) | 0.53 (0.33,0.83) | 0.43 (0.24,0.78) | 0.19 (0.06,0.63) | 0.17 (0.08,0.35) |
| 0.67 (0.31,1.45) | 0.62 (0.31,1.23) | 0.64 (0.49,0.83) | 0.55 (0.28,1.06) | 0.56 (0.37,0.86) | 0.46 (0.26,0.80) | 0.21 (0.07,0.66) | 0.18 (0.09,0.36) |
| 0.64 (0.18,2.24) | 0.59 (0.17,1.96) | 0.60 (0.22,1.69) | 0.52 (0.16,1.72) | 0.53 (0.18,1.56) | 0.43 (0.14,1.36) | 0.20 (0.04,0.90) | 0.17 (0.05,0.57) |
| 0.72 (0.30,1.76) | 0.67 (0.30,1.51) | 0.69 (0.41,1.15) | 0.59 (0.27,1.31) | 0.61 (0.33,1.11) | 0.49 (0.24,1.01) | 0.22 (0.06,0.77) | 0.19 (0.08,0.44) |
| 0.75 (0.34,1.65) | 0.69 (0.34,1.40) | 0.71 (0.52,0.97) | 0.61 (0.31,1.21) | 0.63 (0.40,0.98) | 0.51 (0.28,0.92) | 0.23 (0.07,0.75) | 0.20 (0.10,0.41) |
| 0.89 (0.41,1.95) | 0.82 (0.41,1.65) | 0.85 (0.64,1.13) | 0.73 (0.38,1.42) | 0.75 (0.48,1.15) | 0.61 (0.34,1.08) | 0.28 (0.09,0.88) | 0.24 (0.12,0.49) |
| 0.95 (0.44,2.03) | 0.87 (0.45,1.72) | 0.90 (0.72,1.13) | 0.78 (0.41,1.48) | 0.79 (0.53,1.18) | 0.65 (0.37,1.12) | 0.29 (0.09,0.93) | 0.25 (0.13,0.51) |
| Anlo | 0.92 (0.35,2.42) | 0.82 (0.32,2.11) | 1.71 (0.75,3.92) | 0.84 (0.38,1.86) | 0.68 (0.28,1.65) | 0.31 (0.08,1.19) | 0.27 (0.10,0.71) |
| 1.09 (0.41,2.85) | Cabo | 1.03 (0.55,1.95) | 0.89 (0.37,2.14) | 0.91 (0.44,1.86) | 0.74 (0.33,1.66) | 0.34 (0.09,1.23) | 0.29 (0.12,0.72) |
| 1.22 (0.47,3.13) | 0.97 (0.51,1.83) | Suni | 0.86 (0.47,1.57) | 0.88 (0.63,1.22) | 0.72 (0.43,1.18) | 0.33 (0.11,1.01) | 0.28 (0.15,0.54) |
| 0.58 (0.26,1.34) | 1.12 (0.47,2.69) | 1.16 (0.64,2.11) | Sora | 1.02 (0.51,2.02) | 0.83 (0.38,1.81) | 0.38 (0.15,0.98) | 0.32 (0.25,0.43) |
| 1.20 (0.54,2.66) | 1.10 (0.54,2.26) | 1.14 (0.82,1.58) | 0.98 (0.49,1.95) | LenEvero | 0.81 (0.45,1.48) | 0.37 (0.11,1.20) | 0.32 (0.15,0.67) |
| 1.47 (0.61,3.56) | 1.36 (0.60,3.05) | 1.40 (0.85,2.31) | 1.21 (0.55,2.64) | 1.23 (0.67,2.24) | IMA901Suni | 0.46 (0.13,1.56) | 0.39 (0.17,0.90) |
| 3.23 (0.84,12.37) | 2.98 (0.82,10.87) | 3.07 (0.99,9.49) | 2.65 (1.02,6.89) | 2.70 (0.83,8.75) | 2.20 (0.64,7.55) | Bev | 0.86 (0.34,2.15) |
| 3.75 (1.40,10.01) | 3.46 (1.38,8.63) | 3.56 (1.84,6.89) | 3.08 (2.35,4.04) | 3.14 (1.50,6.56) | 2.55 (1.11,5.84) | 1.16 (0.46,2.90) | Placebo |

Abbreviations: Suni: Sunitinib; Bev: Bevacizumab; Cabo: Cabozantinib; Pazo: Pazopanib; Savo: Savolitinib; Sora: Sorafenib; Axi: Axitinib; Anlo: Anlotinib; PemAxi: Pembrolizumab plus Axitinib; AtezoBev: Atezolizumab plus Bevacizumab; AveAxi: Avelumab plus Axitinib; LenPem: Lenvatinib plus Pembrolizumab; NivoCabo: Nivolumab plus Cabozantinib; NivoIpi: Nivolumab plus lpilimumab; LenEvero: Lenvatinib plus Everolimus; IMA901Suni: IMA901 plus Sunitinib.

Yellow suggests a relative treatment benefit, blue suggests a relative treatment harm, dark red suggests not significant.

Supplementary Table 3E. Matrix of pairwise comparisons of regimens on 24 month overall survival (shown as odds ratio and 95% confidence intervals).

| LenPem | 0.95 (0.62,1.46) | 0.95 (0.59,1.52) | 0.92 (0.58,1.44) | 0.93 (0.45,1.91) | 0.87 (0.47,1.61) | 0.76 (0.49,1.19) | 0.72 (0.36,1.43) | 0.67 (0.43,1.03) | 0.63 (0.42,0.95) | 0.60 (0.43,0.85) | 0.49 (0.31,0.78) | 0.41 (0.23,0.73) |
| --- | --- | --- | --- | --- | --- | --- | --- | --- | --- | --- | --- | --- |
| 1.05 (0.69,1.61) | Nivolpi | 0.99 (0.66,1.50) | 0.96 (0.66,1.42) | 0.98 (0.50,1.93) | 0.91 (0.52,1.61) | 0.80 (0.55,1.17) | 0.76 (0.40,1.44) | 0.70 (0.49,1.01) | 0.66 (0.48,0.92) | 0.63 (0.49,0.81) | 0.51 (0.34,0.77) | 0.43 (0.25,0.73) |
| 1.06 (0.66,1.70) | 1.01 (0.67,1.52) | NivoCabo | 0.97 (0.63,1.50) | 0.98 (0.48,2.00) | 0.92 (0.50,1.68) | 0.81 (0.53,1.24) | 0.76 (0.39,1.50) | 0.71 (0.46,1.08) | 0.67 (0.45,0.99) | 0.64 (0.46,0.88) | 0.52 (0.33,0.82) | 0.43 (0.25,0.76) |
| 1.09 (0.69,1.71) | 1.04 (0.70,1.53) | 1.03 (0.67,1.60) | PemAxi | 1.01 (0.51,2.03) | 0.95 (0.53,1.71) | 0.83 (0.55,1.25) | 0.79 (0.41,1.52) | 0.73 (0.49,1.08) | 0.69 (0.48,0.99) | 0.66 (0.49,0.88) | 0.53 (0.35,0.82) | 0.45 (0.26,0.77) |
| 1.07 (0.52,2.20) | 1.02 (0.52,2.01) | 1.02 (0.50,2.06) | 0.99 (0.49,1.98) | Cabo | 0.94 (0.42,2.10) | 0.82 (0.41,1.63) | 0.78 (0.33,1.84) | 0.72 (0.36,1.42) | 0.68 (0.35,1.32) | 0.65 (0.35,1.21) | 0.53 (0.26,1.06) | 0.44 (0.20,0.96) |
| 1.15 (0.62,2.12) | 1.09 (0.62,1.93) | 1.09 (0.59,1.98) | 1.05 (0.59,1.90) | 1.07 (0.48,2.40) | Axi | 0.88 (0.49,1.57) | 0.83 (0.38,1.81) | 0.77 (0.43,1.36) | 0.73 (0.42,1.26) | 0.69 (0.42,1.15) | 0.56 (0.31,1.02) | 0.47 (0.24,0.94) |
| 1.31 (0.84,2.04) | 1.25 (0.86,1.81) | 1.24 (0.81,1.90) | 1.20 (0.80,1.80) | 1.22 (0.61,2.43) | 1.14 (0.64,2.04) | AveAxi | 0.95 (0.49,1.82) | 0.88 (0.60,1.29) | 0.83 (0.58,1.18) | 0.79 (0.60,1.04) | 0.64 (0.42,0.98) | 0.54 (0.31,0.92) |
| 1.39 (0.70,2.74) | 1.32 (0.69,2.50) | 1.31 (0.67,2.57) | 1.27 (0.66,2.46) | 1.29 (0.54,3.05) | 1.21 (0.55,2.63) | 1.06 (0.55,2.03) | Sora | 0.87 (0.38,1.97) | 0.87 (0.47,1.64) | 0.83 (0.46,1.51) | 0.68 (0.35,1.33) | 0.57 (0.27,1.20) |
| 1.49 (0.97,2.31) | 1.42 (0.99,2.05) | 1.41 (0.93,2.15) | 1.37 (0.92,2.04) | 1.39 (0.70,2.76) | 1.30 (0.73,2.31) | 1.14 (0.78,1.68) | 1.15 (0.51,2.61) | AtezoBev | 0.94 (0.67,1.33) | 1.08 (0.56,2.06) | 0.73 (0.48,1.11) | 0.61 (0.36,1.05) |
| 1.58 (1.05,2.38) | 1.51 (1.08,2.10) | 1.50 (1.01,2.21) | 1.45 (1.01,2.09) | 1.47 (0.76,2.87) | 1.38 (0.79,2.40) | 1.21 (0.85,1.72) | 1.14 (0.61,2.14) | 1.06 (0.75,1.50) | Pazo | 0.95 (0.77,1.19) | 0.78 (0.53,1.14) | 0.65 (0.39,1.08) |
| 1.66 (1.18,2.34) | 1.58 (1.23,2.03) | 1.57 (1.13,2.17) | 1.52 (1.14,2.04) | 1.54 (0.82,2.90) | 1.45 (0.87,2.40) | 1.27 (0.96,1.68) | 1.20 (0.66,2.16) | 0.93 (0.48,1.77) | 1.05 (0.84,1.30) | Suni | 0.81 (0.59,1.11) | 0.68 (0.43,1.08) |
| 2.04 (1.28,3.25) | 1.94 (1.30,2.91) | 1.93 (1.23,3.03) | 1.87 (1.22,2.88) | 1.90 (0.94,3.84) | 1.78 (0.98,3.23) | 1.56 (1.02,2.38) | 1.47 (0.75,2.88) | 1.37 (0.90,2.06) | 1.29 (0.88,1.89) | 1.23 (0.90,1.68) | LenEvero | 0.84 (0.48,1.47) |
| 2.44 (1.37,4.35) | 2.32 (1.37,3.94) | 2.31 (1.31,4.07) | 2.24 (1.29,3.88) | 2.27 (1.04,4.97) | 2.12 (1.07,4.23) | 1.86 (1.08,3.21) | 1.76 (0.83,3.73) | 1.63 (0.96,2.79) | 1.54 (0.92,2.57) | 1.47 (0.92,2.34) | 1.20 (0.68,2.10) | IMA901Suni |

Abbreviations: Suni: Sunitinib; Cabo: Cabozantinib; Pazo: Pazopanib; Sora: Sorafenib; Axi: Axitinib; PemAxi: Pembrolizumab plus Axitinib; AtezoBev: Atezolizumab plus Bevacizumab; AveAxi: Avelumab plus Axitinib; LenPem: Lenvatinib plus Pembrolizumab; NivoCabo: Nivolumab plus Cabozantinib; NivoIpi: Nivolumab plus lpilimumab; LenEvero: Lenvatinib plus Everolimus; IMA901Suni: IMA901 plus Sunitinib. Yellow suggests a relative treatment benefit, blue suggests a relative treatment harm, dark red suggests not significant.

Supplementary Table 3F. Matrix of pairwise comparisons of regimens on 30 month overall survival (shown as odds ratio and 95% confidence intervals).

| AveAxi | 0.96 (0.63,1.45) | 0.87 (0.44,1.74) | 0.83 (0.58,1.20) | 0.82 (0.56,1.21) | 0.82 (0.46,1.45) | 0.79 (0.52,1.21) | 0.72 (0.37,1.38) | 0.63 (0.45,0.89) | 0.60 (0.42,0.88) | 0.58 (0.45,0.76) | 0.45 (0.30,0.67) | 0.40 (0.24,0.67) |
| --- | --- | --- | --- | --- | --- | --- | --- | --- | --- | --- | --- | --- |
| 1.05 (0.69,1.59) | NivoCabo | 0.91 (0.45,1.85) | 0.87 (0.58,1.30) | 0.86 (0.56,1.31) | 0.85 (0.47,1.56) | 0.83 (0.53,1.30) | 0.75 (0.38,1.48) | 0.66 (0.45,0.97) | 0.63 (0.42,0.95) | 0.61 (0.44,0.84) | 0.47 (0.30,0.73) | 0.42 (0.24,0.72) |
| 1.15 (0.58,2.30) | 1.10 (0.54,2.24) | Cabo | 0.96 (0.48,1.89) | 0.95 (0.47,1.90) | 0.94 (0.42,2.12) | 0.91 (0.45,1.86) | 0.82 (0.34,1.98) | 0.73 (0.37,1.43) | 0.69 (0.35,1.38) | 0.67 (0.36,1.27) | 0.51 (0.25,1.04) | 0.46 (0.21,0.99) |
| 1.20 (0.84,1.72) | 1.15 (0.77,1.71) | 1.04 (0.53,2.06) | Nivolpi | 0.99 (0.68,1.43) | 0.98 (0.56,1.72) | 0.95 (0.63,1.43) | 0.86 (0.45,1.65) | 0.76 (0.55,1.05) | 0.73 (0.51,1.04) | 0.70 (0.55,0.89) | 0.54 (0.36,0.79) | 0.48 (0.29,0.79) |
| 1.22 (0.83,1.79) | 1.16 (0.76,1.77) | 1.06 (0.53,2.12) | 1.01 (0.70,1.46) | PemAxi | 0.99 (0.56,1.77) | 0.96 (0.63,1.48) | 0.87 (0.45,1.69) | 0.77 (0.54,1.09) | 0.73 (0.50,1.07) | 0.71 (0.54,0.94) | 0.54 (0.36,0.82) | 0.48 (0.29,0.82) |
| 1.23 (0.69,2.18) | 1.17 (0.64,2.13) | 1.06 (0.47,2.41) | 1.02 (0.58,1.79) | 1.01 (0.56,1.80) | Axi | 0.97 (0.53,1.77) | 0.88 (0.40,1.93) | 0.77 (0.45,1.35) | 0.74 (0.42,1.31) | 0.72 (0.43,1.19) | 0.55 (0.30,0.99) | 0.49 (0.25,0.96) |
| 1.26 (0.83,1.92) | 1.21 (0.77,1.90) | 1.10 (0.54,2.24) | 1.05 (0.70,1.58) | 1.04 (0.68,1.59) | 1.03 (0.56,1.88) | LenPem | 0.90 (0.46,1.79) | 0.80 (0.54,1.18) | 0.76 (0.50,1.16) | 0.74 (0.53,1.02) | 0.56 (0.36,0.88) | 0.50 (0.29,0.87) |
| 1.40 (0.72,2.70) | 1.34 (0.68,2.64) | 1.21 (0.51,2.92) | 1.16 (0.61,2.23) | 1.15 (0.59,2.23) | 1.14 (0.52,2.51) | 1.11 (0.56,2.19) | Sora | 0.88 (0.47,1.68) | 0.91 (0.40,2.10) | 0.82 (0.45,1.49) | 0.62 (0.32,1.23) | 0.55 (0.26,1.17) |
| 1.58 (1.12,2.23) | 1.51 (1.03,2.22) | 1.37 (0.70,2.69) | 1.32 (0.95,1.82) | 1.30 (0.91,1.85) | 1.29 (0.74,2.24) | 1.25 (0.85,1.85) | 1.13 (0.60,2.15) | Pazo | 0.96 (0.68,1.34) | 0.92 (0.74,1.15) | 0.71 (0.48,1.03) | 0.63 (0.38,1.03) |
| 1.66 (1.14,2.41) | 1.58 (1.05,2.39) | 1.44 (0.72,2.86) | 1.38 (0.97,1.97) | 1.36 (0.93,2.00) | 1.35 (0.76,2.39) | 1.31 (0.87,1.99) | 1.09 (0.48,2.51) | 1.05 (0.75,1.47) | AtezoBev | 1.18 (0.61,2.28) | 0.74 (0.49,1.10) | 0.66 (0.39,1.10) |
| 1.71 (1.31,2.24) | 1.64 (1.19,2.25) | 1.49 (0.79,2.82) | 1.43 (1.12,1.82) | 1.41 (1.07,1.86) | 1.40 (0.84,2.32) | 1.36 (0.98,1.88) | 1.23 (0.67,2.24) | 1.08 (0.87,1.34) | 0.84 (0.44,1.63) | Suni | 0.76 (0.56,1.04) | 0.68 (0.44,1.06) |
| 2.24 (1.49,3.37) | 2.14 (1.38,3.33) | 1.95 (0.96,3.95) | 1.87 (1.26,2.76) | 1.85 (1.22,2.79) | 1.83 (1.01,3.32) | 1.78 (1.14,2.77) | 1.60 (0.82,3.16) | 1.42 (0.97,2.06) | 1.35 (0.91,2.03) | 1.31 (0.96,1.78) | LenEvero | 0.89 (0.52,1.53) |
| 2.52 (1.50,4.24) | 2.41 (1.39,4.16) | 2.19 (1.01,4.76) | 2.10 (1.26,3.48) | 2.07 (1.22,3.51) | 2.06 (1.05,4.04) | 1.99 (1.15,3.46) | 1.80 (0.85,3.82) | 1.59 (0.97,2.61) | 1.52 (0.91,2.55) | 1.47 (0.94,2.30) | 1.12 (0.65,1.93) | IMA901Suni |

Abbreviations: Suni: Sunitinib; Cabo: Cabozantinib; Pazo: Pazopanib; Sora: Sorafenib; Axi: Axitinib; PemAxi: Pembrolizumab plus Axitinib; AtezoBev: Atezolizumab plus Bevacizumab; AveAxi: Avelumab plus Axitinib; LenPem: Lenvatinib plus Pembrolizumab; NivoCabo: Nivolumab plus Cabozantinib; NivoIpi: Nivolumab plus lpilimumab; LenEvero: Lenvatinib plus Everolimus; IMA901Suni: IMA901 plus Sunitinib. Yellow suggests a relative treatment benefit, blue suggests a relative treatment harm, dark red suggests not significant.

Supplementary Figure 4. Forst plolt of pairwise comparisons of regimens on 36 months overall survival. OR (95% CI): odds ratio (95% confidence intervals).

Supplementary Table 4A. Matrix of pairwise comparisons of regimens on progression free survival (shown as hazard ratio and 95% credible intervals).

| Suni | 0.85(0.50,1.45) | 2.08(1.12,3.87) | 1.46(1.01,2.19) | 0.42(0.25,0.69) | 0.92(0.64,1.29) | 1.13(0.54,2.35) | 1.41(0.64,3.10) | 0.94(0.58,1.55) | 1.48(0.95,2.03) |
| --- | --- | --- | --- | --- | --- | --- | --- | --- | --- |
| 1.18(0.69,2.02) | IMA901Suni | 2.45(1.09,5.56) | 1.72(0.91,3.41) | 0.49(0.24,1.03) | 1.09(0.57,2.03) | 1.34(0.53,3.29) | 1.67(0.64,4.29) | 1.12(0.54,2.31) | 1.75(0.85,3.17) |
| 0.48(0.26,0.89) | 0.41(0.18,0.92) | Cabo | 0.70(0.34,1.47) | 0.20(0.09,0.44) | 0.44(0.22,0.89) | 0.54(0.21,1.41) | 0.68(0.25,1.83) | 0.46(0.21,1.00) | 0.71(0.33,1.39) |
| 0.69(0.46,0.99) | 0.58(0.29,1.10) | 1.42(0.68,2.91) | PemAxi | 0.29(0.15,0.53) | 0.63(0.36,1.03) | 0.78(0.33,1.74) | 0.97(0.40,2.28) | 0.65(0.34,1.19) | 1.02(0.54,1.61) |
| 2.39(1.44,3.95) | 2.02(0.97,4.22) | 4.96(2.25,11.05) | 3.48(1.89,6.71) | Placebo | 2.20(1.38,3.44) | 2.71(1.21,5.95) | 3.38(1.34,8.51) | 2.25(1.46,3.52) | 3.53(1.77,6.23) |
| 1.08(0.78,1.55) | 0.92(0.49,1.76) | 2.26(1.13,4.62) | 1.58(0.97,2.75) | 0.45(0.29,0.73) | Pazo | 1.23(0.64,2.37) | 1.54(0.66,3.62) | 1.02(0.62,1.74) | 1.61(0.92,2.55) |
| 0.88(0.42,1.86) | 0.75(0.30,1.87) | 1.84(0.71,4.85) | 1.29(0.58,3.04) | 0.37(0.17,0.83) | 0.81(0.42,1.56) | PazoEvero | 1.24(0.43,3.66) | 0.83(0.37,1.93) | 1.30(0.55,2.86) |
| 0.71(0.32,1.55) | 0.60(0.23,1.55) | 1.47(0.55,3.99) | 1.03(0.44,2.50) | 0.30(0.12,0.75) | 0.65(0.28,1.52) | 0.80(0.27,2.33) | Savo | 0.67(0.27,1.68) | 1.04(0.42,2.40) |
| 1.06(0.64,1.72) | 0.90(0.43,1.84) | 2.20(1.00,4.83) | 1.54(0.84,2.93) | 0.44(0.28,0.68) | 0.98(0.57,1.60) | 1.20(0.52,2.72) | 1.50(0.60,3.72) | Sora | 1.57(0.79,2.71) |
| 0.68(0.49,1.05) | 0.57(0.32,1.17) | 1.41(0.72,3.06) | 0.98(0.62,1.84) | 0.28(0.16,0.56) | 0.62(0.39,1.09) | 0.77(0.35,1.83) | 0.96(0.42,2.37) | 0.64(0.37,1.27) | AtezoBev |
| 0.84(0.47,1.49) | 0.71(0.33,1.56) | 1.75(0.75,4.07) | 1.23(0.63,2.50) | 0.35(0.16,0.76) | 0.77(0.39,1.49) | 0.95(0.37,2.41) | 1.19(0.45,3.15) | 0.79(0.38,1.69) | 1.24(0.59,2.34) |
| 0.55(0.33,0.91) | 0.47(0.22,0.98) | 1.14(0.52,2.54) | 0.80(0.43,1.55) | 0.23(0.11,0.47) | 0.51(0.27,0.92) | 0.62(0.25,1.50) | 0.78(0.31,1.96) | 0.52(0.26,1.06) | 0.81(0.41,1.44) |
| 0.75(0.49,1.15) | 0.63(0.32,1.26) | 1.55(0.74,3.31) | 1.09(0.63,1.99) | 0.31(0.19,0.51) | 0.69(0.42,1.11) | 0.85(0.37,1.90) | 1.06(0.44,2.55) | 0.70(0.51,1.00) | 1.11(0.59,1.84) |
| 0.89(0.45,1.77) | 0.76(0.31,1.80) | 1.85(0.73,4.68) | 1.30(0.60,2.89) | 0.37(0.16,0.87) | 0.82(0.38,1.75) | 1.01(0.36,2.74) | 1.26(0.44,3.58) | 0.84(0.36,1.95) | 1.31(0.57,2.77) |
| 0.39(0.24,0.65) | 0.33(0.16,0.69) | 0.81(0.37,1.80) | 0.57(0.31,1.09) | 0.16(0.08,0.33) | 0.36(0.19,0.65) | 0.44(0.18,1.07) | 0.55(0.22,1.39) | 0.37(0.18,0.75) | 0.58(0.29,1.02) |
| 0.65(0.39,1.07) | 0.55(0.26,1.15) | 1.35(0.61,3.00) | 0.95(0.51,1.83) | 0.27(0.13,0.55) | 0.60(0.32,1.09) | 0.74(0.30,1.77) | 0.92(0.36,2.31) | 0.61(0.31,1.24) | 0.96(0.48,1.69) |
| 0.56(0.34,0.92) | 0.47(0.23,0.98) | 1.16(0.53,2.58) | 0.82(0.44,1.57) | 0.23(0.12,0.48) | 0.52(0.28,0.93) | 0.64(0.26,1.53) | 0.79(0.31,1.99) | 0.53(0.26,1.07) | 0.83(0.42,1.45) |
| 0.89(0.55,1.45) | 0.75(0.37,1.56) | 1.85(0.85,4.06) | 1.29(0.71,2.48) | 0.37(0.19,0.75) | 0.82(0.45,1.47) | 1.01(0.41,2.41) | 1.26(0.50,3.13) | 0.84(0.42,1.69) | 1.32(0.67,2.29) |
| 1.13(0.50,2.56) | 0.96(0.36,2.55) | 2.34(0.85,6.58) | 1.65(0.68,4.15) | 0.47(0.18,1.24) | 1.04(0.42,2.50) | 1.28(0.42,3.82) | 1.59(0.51,4.96) | 1.06(0.41,2.78) | 1.66(0.64,3.91) |

Supplementary Table 4A (continued)

| 1.19(0.67,2.12) | 1.82(1.10,3.02) | 1.34(0.87,2.05) | 1.12(0.56,2.24) | 2.56(1.55,4.24) | 1.54(0.93,2.54) | 1.79(1.09,2.94) | 1.12(0.69,1.83) | 0.89(0.39,2.00) |
| --- | --- | --- | --- | --- | --- | --- | --- | --- |
| 1.40(0.64,3.07) | 2.14(1.03,4.48) | 1.58(0.79,3.12) | 1.32(0.56,3.18) | 3.03(1.45,6.30) | 1.81(0.87,3.78) | 2.11(1.02,4.37) | 1.33(0.64,2.73) | 1.05(0.39,2.78) |
| 0.57(0.25,1.32) | 0.88(0.39,1.93) | 0.64(0.30,1.36) | 0.54(0.21,1.37) | 1.23(0.56,2.71) | 0.74(0.33,1.63) | 0.86(0.39,1.89) | 0.54(0.25,1.18) | 0.43(0.15,1.18) |
| 0.82(0.40,1.60) | 1.25(0.65,2.31) | 0.92(0.50,1.60) | 0.77(0.35,1.67) | 1.76(0.91,3.23) | 1.06(0.55,1.94) | 1.23(0.64,2.25) | 0.77(0.40,1.40) | 0.61(0.24,1.47) |
| 2.84(1.32,6.08) | 4.34(2.13,8.86) | 3.20(1.94,5.24) | 2.67(1.15,6.30) | 6.12(3.00,12.49) | 3.67(1.81,7.49) | 4.26(2.10,8.65) | 2.69(1.33,5.40) | 2.11(0.81,5.51) |
| 1.29(0.67,2.55) | 1.97(1.08,3.69) | 1.45(0.90,2.40) | 1.22(0.57,2.66) | 2.78(1.53,5.19) | 1.67(0.92,3.11) | 1.94(1.07,3.60) | 1.22(0.68,2.24) | 0.96(0.40,2.36) |
| 1.05(0.42,2.70) | 1.60(0.67,3.95) | 1.18(0.53,2.68) | 0.99(0.37,2.75) | 2.26(0.94,5.56) | 1.36(0.56,3.34) | 1.57(0.65,3.85) | 0.99(0.42,2.41) | 0.78(0.26,2.37) |
| 0.84(0.32,2.23) | 1.29(0.51,3.25) | 0.95(0.39,2.30) | 0.79(0.28,2.26) | 1.81(0.72,4.56) | 1.09(0.43,2.75) | 1.26(0.50,3.18) | 0.80(0.32,1.99) | 0.63(0.20,1.95) |
| 1.26(0.59,2.65) | 1.92(0.95,3.87) | 1.42(1.00,1.98) | 1.19(0.51,2.76) | 2.72(1.34,5.44) | 1.63(0.80,3.27) | 1.89(0.94,3.78) | 1.19(0.59,2.36) | 0.94(0.36,2.41) |
| 0.81(0.43,1.69) | 1.23(0.70,2.46) | 0.90(0.54,1.70) | 0.76(0.36,1.75) | 1.73(0.98,3.45) | 1.04(0.59,2.07) | 1.20(0.69,2.41) | 0.76(0.44,1.50) | 0.60(0.26,1.56) |
| Atezo | 1.53(0.71,3.29) | 1.13(0.55,2.30) | 0.94(0.39,2.33) | 2.16(1.01,4.62) | 1.29(0.61,2.77) | 1.50(0.70,3.21) | 0.94(0.45,2.00) | 0.74(0.27,2.03) |
| 0.65(0.30,1.40) | AveAxi | 0.74(0.38,1.43) | 0.62(0.26,1.45) | 1.41(0.69,2.88) | 0.85(0.41,1.72) | 0.98(0.48,1.99) | 0.62(0.31,1.25) | 0.49(0.19,1.27) |
| 0.89(0.43,1.82) | 1.36(0.70,2.64) | Axi | 0.84(0.38,1.88) | 1.91(0.99,3.71) | 1.15(0.59,2.23) | 1.33(0.69,2.59) | 0.84(0.44,1.61) | 0.66(0.26,1.67) |
| 1.06(0.43,2.59) | 1.62(0.69,3.79) | 1.19(0.53,2.66) | Anlo | 2.28(0.97,5.32) | 1.37(0.58,3.18) | 1.59(0.68,3.69) | 1.00(0.43,2.30) | 0.79(0.27,2.28) |
| 0.46(0.22,0.99) | 0.71(0.35,1.45) | 0.52(0.27,1.01) | 0.44(0.19,1.03) | LenPem | 0.60(0.29,1.22) | 0.70(0.34,1.42) | 0.44(0.22,0.88) | 0.35(0.13,0.90) |
| 0.77(0.36,1.65) | 1.18(0.58,2.41) | 0.87(0.45,1.68) | 0.73(0.31,1.71) | 1.67(0.82,3.39) | LenEvero | 1.16(0.57,2.36) | 0.73(0.36,1.47) | 0.58(0.22,1.50) |
| 0.67(0.31,1.42) | 1.02(0.50,2.07) | 0.75(0.39,1.44) | 0.63(0.27,1.46) | 1.44(0.71,2.90) | 0.86(0.42,1.74) | NivoCabo | 0.63(0.31,1.26) | 0.50(0.19,1.28) |
| 1.06(0.50,2.25) | 1.62(0.80,3.27) | 1.19(0.62,2.27) | 1.00(0.43,2.32) | 2.28(1.13,4.60) | 1.37(0.68,2.75) | 1.59(0.79,3.20) | Nivolpi | 0.79(0.40,1.54) |
| 1.34(0.49,3.64) | 2.05(0.79,5.36) | 1.51(0.60,3.80) | 1.26(0.44,3.71) | 2.89(1.11,7.54) | 1.73(0.67,4.53) | 2.01(0.78,5.24) | 1.27(0.65,2.47) | Nivo |

Abbreviations: Suni: Sunitinib; Bev: Bevacizumab; Cabo: Cabozantinib; Pazo: Pazopanib; Savo: Savolitinib; Sora: Sorafenib; Axi: Axitinib; Anlo: Anlotinib; Nivo: Nivolumab; Atezo: Atezolizumab; PemAxi: Pembrolizumab plus Axitinib; AtezoBev: Atezolizumab plus Bevacizumab; AveAxi: Avelumab plus Axitinib; LenPem: Lenvatinib plus Pembrolizumab; NivoCabo: Nivolumab plus Cabozantinib; NivoIpi: Nivolumab plus lpilimumab; LenEvero: Lenvatinib plus Everolimus; PazoEvero: Pazopanib plus Everolimus; IMA901Suni: IMA901 plus Sunitinib. Yellow suggests a relative treatment benefit, blue suggests a relative treatment harm, dark red suggests not significant.

Supplementary Table 4B. Matrix of pairwise comparisons of regimens on overall survival (shown as hazard ratio and 95% credible intervals).

| Suni | 0.75(0.41,1.37) | 1.25(0.65,2.39) | 0.92(0.60,1.41) | 2.08(1.07,4.03) | 1.96(0.73,5.36) | 0.37(0.15,0.91) | 0.80(0.40,1.59) |
| --- | --- | --- | --- | --- | --- | --- | --- |
| 1.34(0.73,2.47) | IMA901Suni | 1.67(0.69,4.08) | 1.23(0.59,2.60) | 2.80(1.13,6.86) | 2.63(0.83,8.35) | 0.49(0.17,1.47) | 1.08(0.43,2.69) |
| 0.80(0.42,1.54) | 0.60(0.24,1.46) | Cabo | 0.74(0.34,1.60) | 1.67(0.66,4.24) | 1.58(0.48,5.19) | 0.30(0.10,0.90) | 0.64(0.25,1.65) |
| 1.09(0.71,1.67) | 0.81(0.38,1.70) | 1.36(0.62,2.95) | Pazo | 2.27(1.03,4.97) | 2.15(0.73,6.34) | 0.40(0.15,1.09) | 0.88(0.39,1.96) |
| 0.48(0.25,0.93) | 0.36(0.15,0.88) | 0.60(0.24,1.52) | 0.44(0.20,0.97) | PemAxi | 0.94(0.29,3.13) | 0.18(0.06,0.54) | 0.38(0.15,0.99) |
| 0.51(0.19,1.37) | 0.38(0.12,1.20) | 0.63(0.19,2.08) | 0.47(0.16,1.36) | 1.06(0.32,3.48) | Savo | 0.19(0.05,0.70) | 0.41(0.12,1.33) |
| 2.71(1.10,6.73) | 2.02(0.68,6.05) | 3.38(1.12,10.3) | 2.48(0.92,6.85) | 5.65(1.86,17.22) | 5.33(1.43,19.9) | Placebo | 2.17(1.21,3.94) |
| 1.25(0.63,2.48) | 0.93(0.37,2.34) | 1.55(0.61,4.01) | 1.14(0.51,2.59) | 2.60(1.01,6.71) | 2.45(0.75,8.02) | 0.46(0.25,0.83) | Sora |
| 0.93(0.53,1.64) | 0.69(0.30,1.60) | 1.16(0.49,2.74) | 0.85(0.42,1.75) | 1.94(0.82,4.61) | 1.83(0.60,5.69) | 0.34(0.12,1.00) | 0.75(0.31,1.83) |
| 0.52(0.28,0.96) | 0.39(0.16,0.92) | 0.65(0.26,1.60) | 0.48(0.23,1.01) | 1.08(0.44,2.67) | 1.02(0.32,3.29) | 0.19(0.06,0.57) | 0.42(0.17,1.05) |
| 0.82(0.47,1.44) | 0.61(0.27,1.40) | 1.02(0.44,2.41) | 0.75(0.37,1.53) | 1.71(0.72,4.04) | 1.61(0.53,4.99) | 0.30(0.15,0.62) | 0.66(0.44,0.98) |
| 0.44(0.25,0.78) | 0.33(0.14,0.76) | 0.55(0.23,1.30) | 0.40(0.20,0.83) | 0.92(0.38,2.20) | 0.86(0.28,2.70) | 0.16(0.06,0.48) | 0.35(0.14,0.86) |
| 0.87(0.48,1.56) | 0.65(0.28,1.51) | 1.08(0.45,2.60) | 0.80(0.39,1.66) | 1.81(0.75,4.36) | 1.71(0.55,5.39) | 0.32(0.11,0.95) | 0.70(0.28,1.72) |
| 0.62(0.34,1.12) | 0.46(0.20,1.08) | 0.77(0.32,1.86) | 0.57(0.27,1.19) | 1.29(0.53,3.14) | 1.22(0.39,3.82) | 0.23(0.08,0.68) | 0.50(0.20,1.23) |
| 0.89(0.51,1.56) | 0.66(0.29,1.51) | 1.11(0.47,2.60) | 0.81(0.40,1.66) | 1.85(0.78,4.39) | 1.75(0.57,5.42) | 0.33(0.11,0.95) | 0.71(0.29,1.74) |

Supplementary Table 4B (continued)

| 1.08(0.61,1.89) | 1.93(1.04,3.56) | 1.22(0.69,2.13) | 2.27(1.28,4.03) | 1.15(0.64,2.06) | 1.61(0.89,2.91) | 1.12(0.64,1.98) |
| --- | --- | --- | --- | --- | --- | --- |
| 1.44(0.63,3.30) | 2.58(1.08,6.14) | 1.64(0.71,3.72) | 3.05(1.31,7.03) | 1.54(0.66,3.59) | 2.17(0.93,5.04) | 1.51(0.66,3.46) |
| 0.86(0.36,2.03) | 1.55(0.63,3.78) | 0.98(0.41,2.29) | 1.82(0.77,4.31) | 0.92(0.38,2.20) | 1.30(0.54,3.10) | 0.90(0.39,2.11) |
| 1.17(0.57,2.37) | 2.10(0.99,4.41) | 1.33(0.65,2.68) | 2.48(1.20,5.05) | 1.25(0.60,2.58) | 1.76(0.84,3.64) | 1.23(0.60,2.48) |
| 0.52(0.22,1.23) | 0.92(0.37,2.28) | 0.58(0.25,1.38) | 1.09(0.46,2.62) | 0.55(0.23,1.33) | 0.78(0.32,1.88) | 0.54(0.23,1.28) |
| 0.55(0.18,1.68) | 0.98(0.30,3.15) | 0.62(0.20,1.90) | 1.16(0.37,3.58) | 0.58(0.19,1.82) | 0.82(0.26,2.57) | 0.57(0.18,1.76) |
| 2.91(1.00,8.49) | 5.22(1.75,15.54) | 3.30(1.62,6.77) | 6.16(2.10,18.1) | 3.12(1.06,9.20) | 4.37(1.48,12.94) | 3.05(1.06,8.88) |
| 1.34(0.55,3.27) | 2.40(0.96,6.00) | 1.52(1.02,2.27) | 2.83(1.16,6.96) | 1.43(0.58,3.55) | 2.01(0.81,5.00) | 1.40(0.58,3.42) |
| AtezoBev | 1.79(0.78,4.13) | 1.13(0.51,2.53) | 2.11(0.94,4.74) | 1.07(0.47,2.41) | 1.50(0.66,3.42) | 1.04(0.47,2.34) |
| 0.56(0.24,1.29) | AveAxi | 0.63(0.28,1.45) | 1.18(0.51,2.74) | 0.60(0.26,1.40) | 0.84(0.36,1.97) | 0.58(0.26,1.35) |
| 0.88(0.40,1.96) | 1.58(0.69,3.61) | Axi | 1.86(0.84,4.17) | 0.94(0.42,2.13) | 1.32(0.59,2.99) | 0.92(0.42,2.05) |
| 0.47(0.21,1.06) | 0.85(0.37,1.96) | 0.54(0.24,1.20) | LenPem | 0.51(0.22,1.15) | 0.71(0.31,1.62) | 0.49(0.22,1.11) |
| 0.94(0.41,2.11) | 1.68(0.71,3.92) | 1.06(0.47,2.38) | 1.98(0.87,4.52) | LenEvero | 1.41(0.61,3.22) | 0.98(0.44,2.20) |
| 0.67(0.29,1.51) | 1.19(0.51,2.79) | 0.76(0.33,1.70) | 1.41(0.62,3.21) | 0.71(0.31,1.64) | NivoCabo | 0.70(0.31,1.58) |
| 0.96(0.43,2.12) | 1.71(0.74,3.92) | 1.08(0.49,2.39) | 2.02(0.90,4.52) | 1.02(0.45,2.30) | 1.44(0.63,3.23) | NivIpi |

Abbreviations: Suni: Sunitinib; Cabo: Cabozantinib; Pazo: Pazopanib; Savo: Savolitinib; Sora: Sorafenib; Axi: Axitinib; Nivo: Nivolumab; PemAxi: Pembrolizumab plus Axitinib; AtezoBev: Atezolizumab plus Bevacizumab; AveAxi: Avelumab plus Axitinib; LenPem: Lenvatinib plus Pembrolizumab; NivoCabo: Nivolumab plus Cabozantinib; NivoIpi: Nivolumab plus lpilimumab; LenEvero: Lenvatinib plus Everolimus; IMA901Suni: IMA901 plus Sunitinib.

Yellow suggests a relative treatment benefit, blue suggests a relative treatment harm, dark red suggests not significant.

Supplementary Table 4C. Matrix of pairwise comparisons of regimens on objective response rate (shown as hazard ratio and 95% credible intervals).

| Bev | 0.17(0.01,1.51) | 6.09(0.25,77.25) | 1.52(0.07,15.86) | 3.45(0.16,37.77) | 2.19(0.11,22.77) | 1.86(0.07,30.31) | 1.06(0.05,10.77) | 1.75(0.08,19.55) |
| --- | --- | --- | --- | --- | --- | --- | --- | --- |
| 5.92(0.66,107.61) | Placebo | 36.49(9.82,146.01) | 9.19(3.84,23.51) | 21.05(7.59,60.11) | 13.09(5.78,33.39) | 11.86(2.49,56.65) | 6.37(2.87,14.81) | 10.66(3.94,30.65) |
| 0.16(0.01,4.07) | 0.03(0.01,0.10) | Cabo | 0.25(0.09,0.70) | 0.57(0.18,1.77) | 0.36(0.12,1.12) | 0.32(0.06,1.72) | 0.17(0.05,0.61) | 0.29(0.09,0.91) |
| 0.66(0.06,13.72) | 0.11(0.04,0.26) | 4.01(1.44,11.40) | Suni | 2.28(1.35,3.80) | 1.41(0.93,2.42) | 1.28(0.35,4.65) | 0.71(0.32,1.49) | 1.16(0.72,1.89) |
| 0.29(0.03,6.29) | 0.05(0.02,0.13) | 1.75(0.57,5.53) | 0.44(0.26,0.74) | PemAxi | 0.62(0.33,1.34) | 0.56(0.14,2.27) | 0.31(0.12,0.75) | 0.51(0.25,1.04) |
| 0.46(0.04,9.48) | 0.08(0.03,0.17) | 2.81(0.89,8.61) | 0.71(0.41,1.07) | 1.62(0.74,3.08) | Pazo | 0.91(0.22,3.46) | 0.49(0.2,1.07) | 0.82(0.39,1.53) |
| 0.54(0.03,14.22) | 0.08(0.02,0.40) | 3.14(0.58,16.09) | 0.78(0.22,2.85) | 1.79(0.44,7.23) | 1.11(0.29,4.58) | Savo | 0.54(0.12,2.42) | 0.91(0.23,3.68) |
| 0.94(0.09,18.98) | 0.16(0.07,0.35) | 5.73(1.64,20.7) | 1.44(0.67,3.16) | 3.27(1.33,8.37) | 2.04(0.94,4.95) | 1.84(0.41,8.33) | Sora | 1.66(0.69,4.20) |
| 0.57(0.05,12.24) | 0.09(0.03,0.25) | 3.43(1.10,10.86) | 0.86(0.53,1.40) | 1.96(0.96,3.96) | 1.22(0.66,2.54) | 1.11(0.27,4.42) | 0.62(0.24,1.45) | AtezoBev |
| 0.78(0.06,18.65) | 0.13(0.04,0.45) | 4.78(1.30,18.50) | 1.19(0.53,2.79) | 2.72(1.03,7.44) | 1.69(0.68,4.56) | 1.51(0.32,7.16) | 0.83(0.27,2.52) | 1.38(0.54,3.59) |
| 0.22(0.02,4.91) | 0.04(0.01,0.11) | 1.35(0.41,4.46) | 0.34(0.18,0.64) | 0.77(0.34,1.73) | 0.47(0.23,1.12) | 0.43(0.10,1.78) | 0.24(0.09,0.62) | 0.39(0.18,0.87) |
| 0.43(0.04,8.69) | 0.07(0.03,0.17) | 2.59(0.78,8.69) | 0.65(0.33,1.25) | 1.48(0.64,3.38) | 0.92(0.45,2.04) | 0.83(0.19,3.59) | 0.45(0.27,0.73) | 0.75(0.33,1.71) |
| 0.58(0.04,14.35) | 0.10(0.03,0.36) | 3.56(0.84,14.37) | 0.91(0.33,2.28) | 2.05(0.65,5.97) | 1.27(0.45,3.77) | 1.15(0.22,5.56) | 0.62(0.18,2.09) | 1.04(0.35,2.97) |
| 0.15(0.01,3.38) | 0.02(0.01,0.07) | 0.91(0.28,3.09) | 0.23(0.12,0.44) | 0.52(0.23,1.20) | 0.32(0.16,0.77) | 0.31(0.07,1.25) | 0.16(0.06,0.43) | 0.27(0.12,0.60) |
| 0.32(0.03,7.25) | 0.05(0.02,0.16) | 1.95(0.60,6.67) | 0.49(0.26,0.93) | 1.12(0.49,2.54) | 0.69(0.34,1.62) | 0.63(0.15,2.65) | 0.34(0.13,0.92) | 0.57(0.26,1.27) |
| 0.21(0.02,4.47) | 0.03(0.01,0.10) | 1.25(0.38,4.18) | 0.31(0.16,0.60) | 0.72(0.31,1.62) | 0.44(0.21,1.05) | 0.41(0.09,1.69) | 0.22(0.08,0.58) | 0.36(0.16,0.81) |
| 0.49(0.04,10.99) | 0.08(0.03,0.24) | 2.99(0.93,9.89) | 0.75(0.4,1.39) | 1.71(0.75,3.81) | 1.06(0.52,2.43) | 0.96(0.23,4.03) | 0.52(0.19,1.36) | 0.87(0.39,1.91) |
| 0.81(0.06,19.08) | 0.13(0.03,0.59) | 4.86(0.99,23.48) | 1.21(0.35,4.17) | 2.73(0.72,10.38) | 1.70(0.48,6.63) | 1.54(0.26,9.12) | 0.84(0.19,3.50) | 1.39(0.37,5.39) |

Supplementary Table 4C (continued)

| 1.28(0.05,15.86) | 4.48(0.20,51.38) | 2.32(0.12,24.49) | 1.72(0.07,22.29) | 6.6(0.3,76.67) | 3.11(0.14,35.22) | 4.81(0.22,55.47) | 2.04(0.09,22.72) | 1.23(0.05,17.97) |
| --- | --- | --- | --- | --- | --- | --- | --- | --- |
| 7.76(2.23,26.92) | 27.4(9.29,82.83) | 14.17(6.04,34.98) | 10.3(2.81,39.35) | 40.12(13.46,121.77) | 18.82(6.35,56.79) | 29.36(9.97,90.31) | 12.31(4.22,37.07) | 7.58(1.69,36.46) |
| 0.21(0.05,0.77) | 0.74(0.22,2.44) | 0.39(0.12,1.29) | 0.28(0.07,1.19) | 1.09(0.32,3.59) | 0.51(0.15,1.67) | 0.8(0.24,2.65) | 0.33(0.1,1.08) | 0.21(0.04,1.01) |
| 0.84(0.36,1.9) | 2.98(1.57,5.54) | 1.54(0.81,3.02) | 1.11(0.44,3.02) | 4.36(2.28,8.18) | 2.04(1.07,3.82) | 3.19(1.68,6.16) | 1.33(0.72,2.48) | 0.83(0.24,2.85) |
| 0.37(0.13,0.97) | 1.3(0.58,2.94) | 0.67(0.30,1.55) | 0.49(0.17,1.55) | 1.91(0.83,4.31) | 0.89(0.39,2.03) | 1.41(0.62,3.26) | 0.59(0.26,1.33) | 0.37(0.1,1.39) |
| 0.59(0.22,1.47) | 2.11(0.89,4.29) | 1.08(0.49,2.23) | 0.79(0.27,2.25) | 3.09(1.3,6.38) | 1.44(0.62,2.97) | 2.26(0.96,4.76) | 0.95(0.41,1.93) | 0.59(0.15,2.11) |
| 0.66(0.14,3.10) | 2.33(0.56,9.69) | 1.21(0.28,5.14) | 0.87(0.18,4.63) | 3.39(0.8,14.54) | 1.59(0.38,6.75) | 2.47(0.59,10.75) | 1.04(0.25,4.41) | 0.65(0.11,3.81) |
| 1.21(0.4,3.77) | 4.25(1.61,11.7) | 2.21(1.37,3.76) | 1.60(0.48,5.67) | 6.28(2.32,17.09) | 2.94(1.09,7.94) | 4.56(1.71,12.98) | 1.91(0.73,5.22) | 1.19(0.29,5.15) |
| 0.72(0.28,1.86) | 2.57(1.15,5.62) | 1.33(0.59,3.01) | 0.96(0.34,2.89) | 3.76(1.66,8.29) | 1.76(0.79,3.89) | 2.74(1.23,6.19) | 1.15(0.52,2.55) | 0.72(0.19,2.69) |
| Atezo | 3.55(1.27,9.98) | 1.83(0.63,5.35) | 1.34(0.38,4.75) | 5.19(1.84,14.83) | 2.42(0.86,7.04) | 3.81(1.34,10.94) | 1.59(0.58,4.49) | 1.01(0.22,4.34) |
| 0.28(0.1,0.79) | AveAxi | 0.52(0.21,1.29) | 0.37(0.12,1.24) | 1.47(0.59,3.55) | 0.69(0.28,1.68) | 1.07(0.44,2.7) | 0.45(0.19,1.09) | 0.28(0.07,1.11) |
| 0.55(0.19,1.58) | 1.94(0.77,4.74) | Axi | 0.73(0.23,2.42) | 2.85(1.11,6.98) | 1.34(0.52,3.25) | 2.07(0.83,5.29) | 0.87(0.35,2.12) | 0.54(0.13,2.19) |
| 0.74(0.21,2.62) | 2.67(0.81,8.14) | 1.38(0.41,4.39) | Anlo | 3.91(1.21,11.94) | 1.83(0.57,5.63) | 2.86(0.86,8.88) | 1.21(0.38,3.66) | 0.74(0.15,3.49) |
| 0.19(0.07,0.54) | 0.68(0.28,1.69) | 0.35(0.14,0.91) | 0.26(0.08,0.83) | LenPem | 0.47(0.19,1.15) | 0.73(0.3,1.86) | 0.30(0.13,0.76) | 0.19(0.05,0.77) |
| 0.41(0.14,1.17) | 1.45(0.60,3.57) | 0.75(0.31,1.92) | 0.55(0.18,1.76) | 2.14(0.87,5.19) | LenEvero | 1.56(0.64,3.90) | 0.65(0.27,1.59) | 0.41(0.11,1.64) |
| 0.26(0.09,0.74) | 0.93(0.37,2.28) | 0.48(0.19,1.21) | 0.35(0.11,1.16) | 1.38(0.54,3.32) | 0.64(0.26,1.56) | NivoCabo | 0.42(0.17,1.01) | 0.26(0.06,1.04) |
| 0.63(0.22,1.73) | 2.23(0.92,5.35) | 1.15(0.47,2.85) | 0.83(0.27,2.65) | 3.28(1.31,7.87) | 1.53(0.63,3.71) | 2.38(0.99,5.89) | Nivolpi | 0.62(0.21,1.82) |
| 1.02(0.23,4.54) | 3.55(0.9,14.52) | 1.86(0.46,7.49) | 1.35(0.29,6.65) | 5.23(1.31,20.96) | 2.44(0.61,9.93) | 3.81(0.97,15.74) | 1.61(0.55,4.84) | Nivo |

Abbreviations: Suni: Sunitinib; Bev: Bevacizumab; Cabo: Cabozantinib; Pazo: Pazopanib; Savo: Savolitinib; Sora: Sorafenib; Axi: Axitinib; Anlo: Anlotinib; Nivo: Nivolumab; Atezo: Atezolizumab; PemAxi: Pembrolizumab plus Axitinib; AtezoBev: Atezolizumab plus Bevacizumab; AveAxi: Avelumab plus Axitinib; LenPem: Lenvatinib plus Pembrolizumab; NivoCabo: Nivolumab plus Cabozantinib; NivoIpi: Nivolumab plus lpilimumab; LenEvero: Lenvatinib plus Everolimus. Yellow suggests a relative treatment benefit, blue suggests a relative treatment harm, dark red suggests not significant.

Supplementary Table 4D. Matrix of pairwise comparisons of regimens on grade ≥3 adverse events (shown as hazard ratio and 95% credible intervals).

| Nivo | 1.43 (0.33,6.16) | 1.81 (0.64,5.17) | 2.61 (0.94,7.28) | 2.74 (0.88,8.53) | 2.92 (0.98,8.67) | 4.38 (1.94,9.85) | 4.38 (1.29,14.84) | 5.49 (2.28,13.24) |
| --- | --- | --- | --- | --- | --- | --- | --- | --- |
| 0.70 (0.16,3.02) | Savo | 1.27 (0.33,4.86) | 1.83 (0.49,6.88) | 1.92 (0.47,7.87) | 2.05 (0.52,8.08) | 3.06 (0.91,10.34) | 3.07 (0.70,13.48) | 3.85 (1.14,12.96) |
| 0.55 (0.19,1.57) | 0.79 (0.21,3.01) | Placebo | 1.44 (0.62,3.36) | 1.51 (0.57,4.01) | 1.61 (1.21,2.15) | 2.41 (1.24,4.69) | 2.42 (1.30,4.51) | 3.03 (1.56,5.88) |
| 0.38 (0.14,1.07) | 0.55 (0.15,2.05) | 0.69 (0.30,1.61) | Atezo | 1.05 (0.41,2.71) | 1.12 (0.46,2.73) | 1.67 (0.90,3.12) | 1.68 (0.59,4.79) | 2.10 (1.13,3.92) |
| 0.36 (0.12,1.13) | 0.52 (0.13,2.13) | 0.66 (0.25,1.75) | 0.95 (0.37,2.46) | Anlo | 1.07 (0.39,2.95) | 1.60 (0.72,3.53) | 1.60 (0.50,5.09) | 2.00 (0.91,4.42) |
| 0.34 (0.12,1.02) | 0.49 (0.12,1.93) | 0.62 (0.46,0.83) | 0.89 (0.37,2.19) | 0.94 (0.34,2.60) | Sora | 1.50 (0.73,3.09) | 1.50 (0.86,2.60) | 1.88 (0.91,3.87) |
| 0.23 (0.10,0.51) | 0.33 (0.10,1.10) | 0.41 (0.21,0.80) | 0.60 (0.32,1.12) | 0.63 (0.28,1.38) | 0.67 (0.32,1.38) | Nivolpi | 1.00 (0.40,2.49) | 1.26 (0.89,1.76) |
| 0.23 (0.07,0.77) | 0.33 (0.07,1.43) | 0.41 (0.22,0.77) | 0.60 (0.21,1.70) | 0.63 (0.20,1.99) | 0.67 (0.38,1.16) | 1.00 (0.40,2.48) | Axi | 1.25 (0.50,3.11) |
| 0.18 (0.08,0.44) | 0.26 (0.08,0.88) | 0.33 (0.17,0.64) | 0.48 (0.26,0.89) | 0.50 (0.23,1.10) | 0.53 (0.26,1.10) | 0.80 (0.57,1.12) | 0.80 (0.32,1.98) | AtezoBev |
| 0.12 (0.05,0.27) | 0.17 (0.05,0.55) | 0.21 (0.11,0.39) | 0.31 (0.17,0.55) | 0.32 (0.15,0.68) | 0.34 (0.17,0.68) | 0.51 (0.40,0.65) | 0.51 (0.21,1.24) | 0.64 (0.51,0.82) |
| 0.11 (0.05,0.27) | 0.16 (0.05,0.53) | 0.20 (0.11,0.35) | 0.29 (0.15,0.54) | 0.30 (0.14,0.67) | 0.32 (0.17,0.60) | 0.48 (0.34,0.69) | 0.48 (0.21,1.11) | 0.60 (0.42,0.86) |
| 0.10 (0.04,0.24) | 0.14 (0.04,0.48) | 0.18 (0.09,0.35) | 0.26 (0.14,0.49) | 0.27 (0.12,0.61) | 0.29 (0.14,0.61) | 0.44 (0.30,0.63) | 0.44 (0.17,1.09) | 0.55 (0.38,0.78) |
| 0.10 (0.03,0.29) | 0.14 (0.03,0.54) | 0.17 (0.07,0.44) | 0.25 (0.10,0.62) | 0.26 (0.09,0.73) | 0.28 (0.10,0.74) | 0.42 (0.20,0.87) | 0.42 (0.14,1.28) | 0.52 (0.25,1.10) |
| 0.09 (0.04,0.22) | 0.13 (0.04,0.44) | 0.16 (0.08,0.32) | 0.23 (0.12,0.45) | 0.24 (0.11,0.56) | 0.26 (0.12,0.55) | 0.39 (0.26,0.58) | 0.39 (0.15,1.00) | 0.49 (0.33,0.73) |
| 0.08 (0.03,0.20) | 0.11 (0.03,0.38) | 0.14 (0.06,0.29) | 0.20 (0.10,0.41) | 0.21 (0.09,0.50) | 0.22 (0.10,0.50) | 0.33 (0.20,0.55) | 0.33 (0.12,0.88) | 0.41 (0.25,0.68) |
| 0.06 (0.02,0.14) | 0.08 (0.02,0.28) | 0.10 (0.05,0.21) | 0.15 (0.08,0.29) | 0.16 (0.07,0.36) | 0.17 (0.08,0.36) | 0.25 (0.16,0.38) | 0.25 (0.10,0.64) | 0.31 (0.20,0.48) |
| 0.05 (0.02,0.13) | 0.08 (0.02,0.26) | 0.10 (0.05,0.20) | 0.14 (0.07,0.27) | 0.15 (0.06,0.34) | 0.16 (0.07,0.34) | 0.23 (0.15,0.36) | 0.23 (0.09,0.60) | 0.29 (0.19,0.45) |

Supplementary Table 4D (continued)

| 8.53 (3.65,19.89) | 9.10 (3.74,22.11) | 10.02 (4.12,24.36) | 10.52 (3.50,31.62) | 11.21 (4.53,27.77) | 13.23 (5.10,34.33) | 17.62 (7.05,44.04) | 18.79 (7.50,47.04) |
| --- | --- | --- | --- | --- | --- | --- | --- |
| 5.97 (1.81,19.66) | 6.37 (1.88,21.60) | 7.02 (2.07,23.80) | 7.37 (1.85,29.38) | 7.85 (2.29,26.99) | 9.27 (2.60,32.97) | 12.34 (3.56,42.70) | 13.16 (3.80,45.59) |
| 4.70 (2.53,8.73) | 5.02 (2.87,8.77) | 5.53 (2.82,10.84) | 5.80 (2.28,14.79) | 6.19 (3.08,12.42) | 7.30 (3.42,15.56) | 9.72 (4.78,19.77) | 10.36 (5.09,21.12) |
| 3.26 (1.83,5.80) | 3.48 (1.85,6.56) | 3.83 (2.03,7.23) | 4.02 (1.62,9.98) | 4.29 (2.22,8.30) | 5.06 (2.46,10.43) | 6.74 (3.44,13.21) | 7.18 (3.66,14.12) |
| 3.11 (1.46,6.62) | 3.32 (1.49,7.39) | 3.65 (1.64,8.14) | 3.84 (1.37,10.76) | 4.09 (1.80,9.30) | 4.83 (2.02,11.55) | 6.42 (2.80,14.76) | 6.85 (2.98,15.77) |
| 2.92 (1.47,5.77) | 3.11 (1.66,5.84) | 3.43 (1.65,7.14) | 3.60 (1.35,9.59) | 3.84 (1.80,8.17) | 4.53 (2.01,10.19) | 6.03 (2.80,12.98) | 6.43 (2.98,13.86) |
| 1.95 (1.53,2.48) | 2.08 (1.45,2.98) | 2.29 (1.60,3.28) | 2.40 (1.14,5.06) | 2.56 (1.71,3.84) | 3.02 (1.83,4.99) | 4.03 (2.63,6.16) | 4.29 (2.80,6.59) |
| 1.94 (0.81,4.68) | 2.08 (0.90,4.79) | 2.29 (0.91,5.72) | 2.40 (0.78,7.38) | 2.56 (1.00,6.51) | 3.02 (1.13,8.04) | 4.02 (1.56,10.33) | 4.29 (1.66,11.03) |
| 1.55 (1.22,1.97) | 1.66 (1.16,2.37) | 1.83 (1.28,2.61) | 1.92 (0.91,4.02) | 2.04 (1.37,3.05) | 2.41 (1.46,3.97) | 3.21 (2.10,4.90) | 3.42 (2.23,5.24) |
| Suni | 1.07 (0.82,1.39) | 1.18 (0.90,1.53) | 1.23 (0.61,2.49) | 1.32 (0.95,1.82) | 1.55 (1.00,2.40) | 2.07 (1.46,2.93) | 2.20 (1.55,3.14) |
| 0.94 (0.72,1.22) | Pazo | 1.10 (0.76,1.61) | 1.16 (0.55,2.45) | 1.23 (0.81,1.87) | 1.45 (0.87,2.43) | 1.94 (1.25,3.00) | 2.06 (1.33,3.21) |
| 0.85 (0.65,1.11) | 0.91 (0.62,1.32) | PemAxi | 1.05 (0.49,2.23) | 1.12 (0.74,1.70) | 1.32 (0.79,2.20) | 1.76 (1.13,2.73) | 1.87 (1.20,2.92) |
| 0.81 (0.40,1.64) | 0.87 (0.41,1.83) | 0.95 (0.45,2.02) | Cabo | 1.07 (0.49,2.31) | 1.26 (0.55,2.88) | 1.67 (0.76,3.67) | 1.79 (0.81,3.92) |
| 0.76 (0.55,1.05) | 0.81 (0.53,1.23) | 0.89 (0.59,1.36) | 0.94 (0.43,2.03) | NivoCabo | 1.18 (0.69,2.03) | 1.57 (0.98,2.53) | 1.68 (1.04,2.70) |
| 0.64 (0.42,1.00) | 0.69 (0.41,1.15) | 0.76 (0.45,1.26) | 0.79 (0.35,1.82) | 0.85 (0.49,1.46) | IMA901Suni | 1.33 (0.76,2.33) | 1.42 (0.81,2.49) |
| 0.48 (0.34,0.69) | 0.52 (0.33,0.80) | 0.57 (0.37,0.88) | 0.60 (0.27,1.31) | 0.64 (0.40,1.02) | 0.75 (0.43,1.31) | LenPem | 1.07 (0.65,1.75) |
| 0.45 (0.32,0.65) | 0.48 (0.31,0.75) | 0.53 (0.34,0.83) | 0.56 (0.25,1.23) | 0.60 (0.37,0.96) | 0.70 (0.40,1.24) | 0.94 (0.57,1.54) | LenEvero |

Abbreviations: Suni: Sunitinib; Cabo: Cabozantinib; Pazo: Pazopanib; Savo: Savolitinib; Sora: Sorafenib; Axi: Axitinib; Anlo: Anlotinib; Nivo: Nivolumab; Atezo: Atezolizumab; PemAxi: Pembrolizumab plus Axitinib; AtezoBev: Atezolizumab plus Bevacizumab; LenPem: Lenvatinib plus Pembrolizumab; NivoCabo: Nivolumab plus Cabozantinib; NivoIpi: Nivolumab plus lpilimumab; LenEvero: Lenvatinib plus Everolimus; IMA901Suni: IMA901 plus Sunitinib. Yellow suggests a relative treatment benefit, blue suggests a relative treatment harm, dark red suggests not significant.

Supplementary Figure 5A. Convergence of the three Markov Chain Monte Carlo chains established by of the history feature for progression free survival. Suni: Sunitinib; Bev: Bevacizumab; Cabo: Cabozantinib; Pazo: Pazopanib; Savo: Savolitinib; Sora: Sorafenib; Axi: Axitinib; Anlo: Anlotinib; Nivo: Nivolumab; Atezo: Atezolizumab; Pem_Axi: Pembrolizumab plus Axitinib; Atezo_Bev: Atezolizumab plus Bevacizumab; Ave_Axi: Avelumab plus Axitinib; Len_Pem: Lenvatinib plus Pembrolizumab; Nivo_Cabo: Nivolumab plus Cabozantinib; Nivo_Ipi: Nivolumab plus lpilimumab; Len_Evero: Lenvatinib plus Everolimus; Pazo_Evero: Pazopanib plus Everolimus; IMA901_Suni: IMA901 plus Sunitinib.

Supplementary Figure 5B. Convergence of the three Markov Chain Monte Carlo chains established by of the history feature for overall survival. Suni: Sunitinib; Cabo: Cabozantinib; Pazo: Pazopanib; Savo: Savolitinib; Sora: Sorafenib; Axi: Axitinib; Nivo: Nivolumab; Pem_Axi: Pembrolizumab plus Axitinib; Atezo_Bev: Atezolizumab plus Bevacizumab; Ave_Axi: Avelumab plus Axitinib; Len_Pem: Lenvatinib plus Pembrolizumab; Nivo_Cabo: Nivolumab plus Cabozantinib; Nivo_Ipi: Nivolumab plus lpilimumab; Len_Evero: Lenvatinib plus Everolimus; IMA901_Suni: IMA901 plus Sunitinib.

Supplementary Figure 5C. Convergence of the three Markov Chain Monte Carlo chains established by of the history feature for objective response rate. Suni: Sunitinib; Bev: Bevacizumab; Cabo: Cabozantinib; Pazo: Pazopanib; Savo: Savolitinib; Sora: Sorafenib; Axi: Axitinib; Anlo: Anlotinib; Nivo: Nivolumab; Atezo: Atezolizumab; Pem_Axi: Pembrolizumab plus Axitinib; Atezo_Bev: Atezolizumab plus Bevacizumab; Ave_Axi: Avelumab plus Axitinib; Len_Pem: Lenvatinib plus Pembrolizumab; Nivo_Cabo: Nivolumab plus Cabozantinib; Nivo_Ipi: Nivolumab plus lpilimumab; Len_Evero: Lenvatinib plus Everolimus.

Supplementary Figure 5D. Convergence of the three Markov Chain Monte Carlo chains established by of the history feature for grade ≥3 adverse events. Suni: Sunitinib; Cabo: Cabozantinib; Pazo: Pazopanib; Savo: Savolitinib; Sora: Sorafenib; Axi: Axitinib; Anlo: Anlotinib; Nivo: Nivolumab; Atezo: Atezolizumab; Pem_Axi: Pembrolizumab plus Axitinib; Atezo_Bev: Atezolizumab plus Bevacizumab; Len_Pem: Lenvatinib plus Pembrolizumab; Nivo_Cabo: Nivolumab plus Cabozantinib; Nivo_Ipi: Nivolumab plus lpilimumab; Len_Evero: Lenvatinib plus Everolimus; IMA901_Suni: IMA901 plus Sunitinib.

Supplementary Figure 6A. Convergence of the three Markov Chain Monte Carlo chains established by of the Brooks-Gelman-Rubin diagnostic for progression free survival. Suni: Sunitinib; Bev: Bevacizumab; Cabo: Cabozantinib; Pazo: Pazopanib; Savo: Savolitinib; Sora: Sorafenib; Axi: Axitinib; Anlo: Anlotinib; Nivo: Nivolumab; Atezo: Atezolizumab; Pem_Axi: Pembrolizumab plus Axitinib; Atezo_Bev: Atezolizumab plus Bevacizumab; Ave_Axi: Avelumab plus Axitinib; Len_Pem: Lenvatinib plus Pembrolizumab; Nivo_Cabo: Nivolumab plus Cabozantinib; Nivo_Ipi: Nivolumab plus lpilimumab; Len_Evero: Lenvatinib plus Everolimus; Pazo_Evero: Pazopanib plus Everolimus; IMA901_Suni: IMA901 plus Sunitinib.

Supplementary Figure 6B. Convergence of the three Markov Chain Monte Carlo chains established by of the Brooks-Gelman-Rubin diagnostic for overall survival. Suni: Sunitinib; Cabo: Cabozantinib; Pazo: Pazopanib; Savo: Savolitinib; Sora: Sorafenib; Axi: Axitinib; Nivo: Nivolumab; PemAxi: Pembrolizumab plus Axitinib; AtezoBev: Atezolizumab plus Bevacizumab; AveAxi: Avelumab plus Axitinib; LenPem: Lenvatinib plus Pembrolizumab; NivoCabo: Nivolumab plus Cabozantinib; NivoIpi: Nivolumab plus lpilimumab; LenEvero: Lenvatinib plus Everolimus; IMA901Suni: IMA901 plus Sunitinib.

Supplementary Figure 6C. Convergence of the three Markov Chain Monte Carlo chains established by of the Brooks-Gelman-Rubin diagnostic for objective response rate. Suni: Sunitinib; Bev: Bevacizumab; Cabo: Cabozantinib; Pazo: Pazopanib; Savo: Savolitinib; Sora: Sorafenib; Axi: Axitinib; Anlo: Anlotinib; Nivo: Nivolumab; Atezo: Atezolizumab; Pem_Axi: Pembrolizumab plus Axitinib; Atezo_Bev: Atezolizumab plus Bevacizumab; Ave_Axi: Avelumab plus Axitinib; Len_Pem: Lenvatinib plus Pembrolizumab; Nivo_Cabo: Nivolumab plus Cabozantinib; Nivo_Ipi: Nivolumab plus lpilimumab; Len_Evero: Lenvatinib plus Everolimus.

Supplementary Figure 6D. Convergence of the three Markov Chain Monte Carlo chains established by of the Brooks-Gelman-Rubin diagnostic for grade ≥3 adverse events. Suni: Sunitinib; Cabo: Cabozantinib; Pazo: Pazopanib; Savo: Savolitinib; Sora: Sorafenib; Axi: Axitinib; Anlo: Anlotinib; Nivo: Nivolumab; Atezo: Atezolizumab; Pem_Axi: Pembrolizumab plus Axitinib; Atezo_Bev: Atezolizumab plus Bevacizumab; Len_Pem: Lenvatinib plus Pembrolizumab; Nivo_Cabo: Nivolumab plus Cabozantinib; Nivo_Ipi: Nivolumab plus lpilimumab; Len_Evero: Lenvatinib plus Everolimus; IMA901_Suni: IMA901 plus Sunitinib.

Supplementary Table 5A. Matrix of pairwise comparisons of regimens on progression free survival (shown as hazard ratio and 95% credible intervals). (meta-regression analysis).

| Anlo | 1.31(0.41,4.01) | 1.50(0.55,3.80) | 2.00(0.62,6.80) | 1.40(0.51,3.80) | 2.20(0.71,6.90) | 0.71(0.29,1.80) | 1.50(0.59,3.70) | 2.50(0.97,6.10) | 0.50(0.64,3.50) |
| --- | --- | --- | --- | --- | --- | --- | --- | --- | --- |
| 0.79(0.25,2.50) | Atezo | 1.20(0.55,2.40) | 1.60(0.70,3.60) | 1.10(0.51,2.30) | 1.70(0.72,4.10) | 0.56(0.17,1.80) | 1.20(0.47,2.80) | 1.90(0.79,4.70) | 0.40(0.27,4.50) |
| 0.66(0.26,1.80) | 0.84(0.42,1.81) | Atezobev | 1.30(0.69,2.90) | 0.93(0.53,1.80) | 1.50(0.71,3.20) | 0.47(0.18,1.30) | 0.97(0.50,2.10) | 1.60(0.84,3.50) | 0.33(0.28,3.40) |
| 0.49(0.15,1.60) | 0.63(0.28,1.40) | 0.75(0.34,1.50) | AveAxi | 0.69(0.33,1.40) | 1.10(0.47,2.50) | 0.35(0.11,1.20) | 0.73(0.29,1.80) | 1.20(0.48,3.10) | 0.25(0.15,3.20) |
| 0.71(0.26,2.00) | 0.90(0.43,1.90) | 1.10(0.56,1.90) | 1.40(0.69,3.00) | Axi | 1.60(0.72,3.40) | 0.51(0.18,1.50) | 1.00(0.50,2.30) | 1.70(0.82,3.80) | 0.36(0.28,3.70) |
| 0.46(0.15,1.40) | 0.58(0.24,1.40) | 0.69(0.31,1.40) | 0.69(0.39,2.20) | 0.64(0.29,1.40) | Cabo | 0.33(0.10,1.00) | 0.67(0.27,1.60) | 1.10(0.45,2.80) | 0.23(0.17,2.50) |
| 1.49(0.56,3.50) | 1.80(0.55,6.00) | 2.10(0.75,5.60) | 2.80(0.82,10.00) | 2.00(0.69,5.70) | 3.10(0.96,10.00) | IMA901Suni | 2.10(0.84,5.00) | 3.40(1.40,8.40) | 0.70(0.11,4.00) |
| 0.68(0.27,1.70) | 0.87(0.35,2.10) | 1.00(0.47,2.00) | 1.40(0.55,3.50) | 0.96(0.44,2.00) | 2.30(0.32,17.40) | 0.48(0.20,1.20) | LenEvero | 1.70(0.79,3.50) | 0.34(0.35,2.80) |
| 0.41(0.16,1.00) | 0.52(0.21,1.33) | 0.62(0.28,1.20) | 0.83(0.32,2.10) | 0.57(0.26,1.20) | 0.90(0.36,2.20) | 0.29(0.12,0.72) | 0.60(0.28,1.30) | LenPem | 0.20(0.21,1.70) |
| 2.00(0.29,16.00) | 2.50(0.22,37.03) | 3.00(0.29,35.00) | 4.00(0.31,66.00) | 2.80(0.27,35.00) | 4.40(0.39,60.00) | 1.40(0.25,9.10) | 2.90(0.36,28.00) | 4.90(0.60,47.00) | Nivo |
| 0.66(0.27,1.60) | 0.83(0.27,2.72) | 0.99(0.36,2.50) | 1.30(0.40,4.70) | 0.92(0.34,2.60) | 1.40(0.47,4.60) | 0.47(0.22,1.00) | 0.97(0.47,2.30) | 1.60(0.68,3.80) | 0.33(0.51,1.90) |
| 1.60(0.26,11.00) | 2.00(0.19,26.01) | 2.40(0.26,25.00) | 3.10(0.27,48.00) | 2.20(0.24,26.00) | 3.40(0.34,44.00) | 1.10(0.23,6.30) | 2.30(0.32,20.00) | 3.80(0.54,34.00) | 0.79(0.39,1.60) |
| 1.20(0.53,2.70) | 1.50(0.63,3.60) | 1.80(0.88,3.30) | 2.40(0.96,6.10) | 1.70(0.85,3.20) | 2.60(1.10,6.20) | 0.84(0.40,1.80) | 1.70(0.91,3.30) | 2.90(1.50,5.60) | 0.59(0.71,4.20) |
| 0.95(0.34,2.70) | 1.20(0.41,3.70) | 1.40(0.54,3.40) | 1.90(0.62,3.10) | 1.30(0.52,3.40) | 2.10(0.70,6.30) | 0.68(0.25,1.90) | 1.40(0.55,3.50) | 2.30(0.92,5.90) | 0.48(0.52,3.80) |
| 0.77(0.34,1.74) | 0.97(0.36,2.60) | 1.20(0.50,2.40) | 1.60(0.55,4.40) | 1.10(0.46,2.40) | 1.70(0.63,4.40) | 0.55(0.26,1.10) | 1.10(0.54,2.20) | 1.90(0.90,3.70) | 0.38(0.51,2.40) |
| 2.40(0.96,6.30) | 3.10(1.30,7.40) | 3.70(1.70,7.00) | 4.90(2.00,12.00) | 3.40(1.90,6.00) | 5.30(2.20,13.00) | 1.70(0.68,4.50) | 3.60(1.70,7.70) | 6.00(2.80,13.00) | 1.20(0.12,11.00) |
| 0.66(0.19,2.30) | 0.84(0.31,2.30) | 1.00(0.51,3.50) | 1.30(0.51,3.50) | 0.93(0.37,2.30) | 1.50(0.52,4.00) | 0.47(0.13,1.70) | 0.98(0.35,2.70) | 1.60(0.58,4.60) | 0.33(0.22,4.10) |
| 1.00(0.38,2.84) | 1.30(0.58,3.04) | 1.60(0.77,2.80) | 2.10(0.93,4.70) | 1.50(0.99,4.70) | 2.30(0.99,2.10) | 0.74(0.26,2.00) | 1.50(0.70,3.30) | 2.50(1.20,5.50) | 0.52(0.44,5.00) |
| 1.00(0.48,2.20) | 1.30(0.65,2.68) | 1.60(0.93,2.30) | 2.10(1.02,4.40) | 1.50(0.85,2.40) | 2.30(1.10,4.60) | 0.74(0.34,1.60) | 1.50(0.89,2.60) | 2.50(1.50,4.30) | 0.52(0.54,4.00) |

Supplementary Table 5A (continued)

| 1.50(0.62,3.70) | 0.64(0.09,3.90) | 0.85(0.38,1.90) | 1.00(0.37,3.00) | 1.30(0.59,3.00) | 0.41(0.16,1.00) | 1.50(0.43,5.30) | 0.97(0.35,2.60) | 0.97(0.45,2.10) |
| --- | --- | --- | --- | --- | --- | --- | --- | --- |
| 1.20(0.37,3.70) | 0.51(0.38,5.20) | 0.67(0.28,1.60) | 0.83(0.27,2.50) | 1.00(0.39,2.70) | 0.32(0.13,0.77) | 1.20(0.44,3.20) | 0.76(0.34,1.70) | 0.76(0.38,1.50) |
| 1.00(0.40,2.70) | 0.42(0.39,3.90) | 0.56(0.30,1.10) | 0.70(0.28,1.90) | 0.86(0.42,2.00) | 0.27(0.14,0.57) | 1.00(0.42,2.60) | 0.64(0.35,1.30) | 0.64(0.43,1.10) |
| 0.75(0.21,2.50) | 0.32(0.21,3.60) | 0.42(0.16,1.00) | 0.52(0.16,1.60) | 0.64(0.23,1.80) | 0.20(0.08,0.49) | 0.74(0.29,2.00) | 0.48(0.21,1.10) | 0.48(0.23,0.99) |
| 1.10(0.39,3.00) | 0.46(0.39,4.20) | 0.61(0.31,1.20) | 0.75(0.29,1.90) | 0.93(0.42.2.20) | 0.29(0.17,0.52) | 1.10(0.44,2.70) | 0.69(0.48,1.00) | 0.69(0.42,1.20) |
| 0.69(0.22,2.10) | 0.29(0.23,2.90) | 0.39(0.16,0.92) | 0.48(0.16,1.40) | 0.59(0.23,1.60) | 0.19(0.08,0.45) | 0.69(0.25,1.90) | 0.44(0.19,1.00) | 0.44(0.22,0.90) |
| 2.10(0.99,4.60) | 0.89(0.16,4.40) | 1.20(0.56,2.50) | 1.50(0.53,4.00) | 1.80(0.90,3.80) | 0.58(0.22,1.50) | 2.10(0.58,7.90) | 1.40(0.49,3.80) | 1.40(0.64,2.90) |
| 1.00(0.44,2.40) | 0.43(0.50,3.10) | 0.58(0.30,1.10) | 0.71(0.28,1.80) | 0.88(0.45,1.90) | 0.28(0.13,0.59) | 1.00(0.37,2.90) | 0.66(0.30,1.40) | 0.66(0.39,1.40) |
| 0.62(0.26,1.50) | 0.26(0.30,1.90) | 0.35(0.18,0.66) | 0.43(0.17,1.10) | 0.53(0.28,1.10) | 0.17(0.08,0.35) | 0.60(0.21,1.7) | 0.40(0.18,0.86) | 0.40(0.23,0.67) |
| 3.10(0.53,20.00) | 1.30(0.64,2.50) | 1.70(0.24,14.00) | 2.10(0.26,19.00) | 2.60(0.41,20.00) | 0.82(0.95,8.40) | 3.00(0.25,46.00) | 1.90(0.20,23.00) | 1.90(0.25,18.00) |
| NivoCabo | 0.42(0.74,2.10) | 0.56(0.27,1.10) | 0.69(0.26,1.80) | 0.86(0.44,1.70) | 0.27(0.11,0.66) | 0.99(0.28,3.60) | 0.64(0.24,1.70) | 0.64(0.32,1.30) |
| 2.40(0.48,14.00) | Nivolpi | 1.30(0.21,10.00) | 1.60(0.23,14.00) | 2.00(0.37,14.00) | 0.64(0.84,6.00) | 2.30(0.21,33.00) | 1.50(0.17,17.00) | 1.50(0.22,13.00) |
| 1.80(0.88,3.70) | 0.75(0.10,4.70) | Pazo | 1.20(0.63,2.40) | 1.50(0.90,2.80) | 0.48(0.29,0.81) | 1.80(0.65,5.00) | 1.10(0.60,2.20) | 1.10(0.77,1.70) |
| 1.40(0.55,3.90) | 0.61(0.73,4.30) | 0.81(0.41,1.60) | PazoEvero | 1.20(0.53,3.00) | 0.39(0.17,0.91) | 1.40(0.43,4.90) | 0.92(0.37,2.30) | 0.93(0.42,2.00) |
| 1.20(0.57,2.30) | 0.49(0.72,2.70) | 0.66(0.36,1.10) | 0.81(0.33,1.90) | PemAxi | 0.32(0.15,0.64) | 1.20(0.38,3.50) | 0.75(0.32,1.60) | 0.75(0.44,1.20) |
| 3.70(1.50,9.10) | 1.60(0.17,12.00) | 2.10(1.20,3.50) | 2.50(1.10,6.00) | 3.20(1.60,6.90) | Placebo | 3.70(1.30,10.00) | 2.40(1.40,3.90) | 2.40(1.40,4.00) |
| 1.00(0.28,3.50) | 0.43(0.31,4.70) | 0.57(0.20,1.50) | 0.70(0.20,1.50) | 0.86(0.29,2.60) | 0.27(0.11,0.74) | Savo | 0.64(0.25,1.70) | 0.64(0.27,1.50) |
| 1.60(0.58,4.20) | 0.66(0.60,5.07) | 0.88(0.46,1.70) | 1.10(0.43,2.70) | 1.30(0.61,3.10) | 0.42(0.26,0.69) | 1.60(0.60,4.10) | Sora | 1.00(0.58,1.70) |
| 1.60(0.76,3.20) | 0.66(0.76,4.50) | 0.88(0.58,1.30) | 1.10(0.50,2.40) | 1.30(0.83,2.30) | 0.42(0.25,0.71) | 1.60(0.65,3.70) | 1.00(0.58,1.70) | Suni |

Abbreviations: Suni: Sunitinib; Bev: Bevacizumab; Cabo: Cabozantinib; Pazo: Pazopanib; Savo: Savolitinib; Sora: Sorafenib; Axi: Axitinib; Anlo: Anlotinib; Nivo: Nivolumab; Atezo: Atezolizumab; PemAxi: Pembrolizumab plus Axitinib; AtezoBev: Atezolizumab plus Bevacizumab; AveAxi: Avelumab plus Axitinib; LenPem: Lenvatinib plus Pembrolizumab; NivoCabo: Nivolumab plus Cabozantinib; NivoIpi: Nivolumab plus lpilimumab; LenEvero: Lenvatinib plus Everolimus; PazoEvero: Pazopanib plus Everolimus; IMA901Suni: IMA901 plus Sunitinib. Yellow suggests a relative treatment benefit, blue suggests a relative treatment harm, dark red suggests not significant.

Supplementary Table 5B. Matrix of pairwise comparisons of regimens on overall survival (shown as hazard ratio and 95% credible intervals). (meta-regression analysis).

| AtezoBev | 1.90(0.85,3.60) | 1.20(0.46,4.30) | 1.20(0.48,3.40) | 0.65(0.49,2.90) | 1.00(0.38,2.50) | 2.00(1.10,4.10) | 0.87(0.23,48.00) |
| --- | --- | --- | --- | --- | --- | --- | --- |
| 0.53(0.10,1.60) | AveAxi | 0.62(0.26,1.50) | 0.62(0.20,1.70) | 0.35(0.61,2.90) | 0.56(0.56,2.20) | 1.10(0.11,4.40) | 0.47(0.70,5.12) |
| 0.85(0.23,2.20) | 1.60(0.69,3.90) | Axi | 1.00(0.41,2.40) | 0.56(0.15,3.70) | 0.89(0.13,3.00) | 1.80(0.26,5.70) | 0.75(0.68,7.50) |
| 0.83(0.29,2.10) | 1.60(0.60,5.00) | 1.00(0.42,2.50) | Cabo | 0.54(0.21,3.30) | 0.86(0.18,2.70) | 1.70(0.36,5.20) | 0.73(0.94,55.00) |
| 1.50(0.35,20.00) | 2.90(0.35,1.60) | 1.80(0.27,68.00) | 1.60(0.31,47.00) | IMA901Suni | 1.60(0.48,11.00) | 3.20(1.40,6.30) | 1.30(0.39,24.00) |
| 0.97(0.40,2.70) | 1.80(0.44,18.00) | 1.10(0.34,7.60) | 1.20(0.37,5.60) | 0.61(0.90,2.10) | LenEvero | 2.00(1.40,4.90) | 0.82(0.43,33.00) |
| 0.49(0.25,0.88) | 0.91(0.23,8.90) | 0.57(0.17,3.80) | 0.59(0.19,2.80) | 0.31(0.16,0.71) | 0.51(0.20,0.72) | LenPem | 0.42(0.21,17.00) |
| 1.10(0.21,4.40) | 2.10(0.20,37.00) | 1.30(0.15,1.50) | 1.40(0.18,1.10) | 0.76(0.42,2.50) | 1.20(0.31,2.30) | 2.40(0.06,4.70) | NivIpi |
| 0.71(0.17,8.40) | 1.30(0.17,68.00) | 0.83(0.13,27.00) | 0.85(0.15,20.00) | 0.46(0.20,0.92) | 0.76(0.23,4.70) | 1.50(0.46,9.30) | 0.61(0.16,11.00) |
| 1.20(0.44,6.00) | 2.30(0.45,47.00) | 1.40(0.34,19.00) | 1.50(0.39,14.00) | 0.78(0.24,1.90) | 1.30(0.57,3.60) | 2.60(1.10,7.00) | 1.10(0.12,29.00) |
| 0.55(0.16,3.70) | 1.00(0.16,29.00) | 0.64(0.12,12.00) | 0.66(0.14,8.50) | 0.34(0.12,0.92) | 0.58(0.24,0.87) | 1.10(0.40,4.30) | 0.46(0.67,12.00) |
| 2.80(1.30,4.70) | 5.40(1.70,17.00) | 3.30(1.60,6.70) | 3.30(1.10,10.00) | 1.80(0.46,14.00) | 2.90(1.39,12.00) | 5.80(1.78,23.00) | 2.50(0.22,2.40) |
| 0.52(0.13,1.80) | 1.00(0.31,3.60) | 0.63(0.21,1.90) | 0.63(0.19,2.10) | 0.34(0.10,2.70) | 0.54(0.84,2.30) | 1.10(0.17,4.50) | 0.46(0.47,2.89) |
| 1.30(0.34,3.60) | 2.50(1.95,6.40) | 1.50(1.00,2.30) | 1.50(0.57,3.90) | 0.85(0.22,6.00) | 1.40(0.19,4.80) | 2.70(1.39,9.30) | 1.10(0.00,1.10) |
| 1.10(0.51,3.70) | 2.10(0.51,29.00) | 1.30(0.40,12.00) | 1.30(0.44,8.40) | 0.71(0.16,1.80) | 1.20(0.63,2.30) | 1.90(1.30,4.50) | 0.96(0.01,31.00) |

Supplementary Table 5B (continued)

| 1.40(0.12,5.80) | 0.81(0.17,2.30) | 1.80(0.27,6.30) | 0.36(0.21,0.74) | 1.90(0.56,7.50) | 0.78(0.28,3.00) | 0.90(0.27,2.00) |
| --- | --- | --- | --- | --- | --- | --- |
| 0.76(0.15,5.80) | 0.44(0.21,2.20) | 0.98(0.34,6.10) | 0.19(0.06,0.57) | 0.99(0.28,3.30) | 0.41(0.16,0.89) | 0.48(0.35,1.90) |
| 1.20(0.37,7.70) | 0.70(0.52,2.90) | 1.60(0.85,8.10) | 0.30(0.15,0.62) | 1.60(0.52,4.90) | 0.66(0.44,0.98) | 0.77(0.86,2.50) |
| 1.20(0.51,6.70) | 0.68(0.72,2.60) | 1.50(0.12,7.10) | 0.30(0.10,0.95) | 1.60(0.48,5.20) | 0.66(0.25,1.80) | 0.75(0.12,2.30) |
| 2.20(0.93,5.10) | 1.30(0.52,4.20) | 2.90(0.11,8.60) | 0.55(0.70,2.00) | 3.00(0.38,98.00) | 1.20(0.17,46.00) | 1.40(0.55,6.20) |
| 1.30(0.21,4.30) | 0.77(0.28,1.80) | 1.70(1.10,4.20) | 0.34(0.24,0.96) | 1.90(0.44,12.00) | 0.74(0.21,5.10) | 0.85(0.44,1.60) |
| 0.67(0.11,2.20) | 0.39(0.14,0.89) | 0.87(0.23,2.50) | 0.17(0.11,0.36) | 0.93(0.22,5.90) | 0.38(0.11,0.74) | 0.43(0.22,0.79) |
| 1.60(0.09,6.20) | 0.95(0.35,8.50) | 2.20(0.85,1.50) | 0.41(0.042,4.60) | 2.20(0.24,2.10) | 0.88(0.95,9.80) | 1.00(0.33,1.40) |
| NivoCabo | 0.59(0.25,1.80) | 1.30(0.52,3.70) | 0.25(0.34,0.62) | 1.40(0.18,41.00) | 0.55(0.81,18.00) | 0.65(0.27,2.60) |
| 1.70(0.55,4.00) | Pazo | 2.20(0.98,4.90) | 0.44(0.28,0.46) | 2.40(0.46,29.00) | 0.94(0.21,13.00) | 1.10(0.69,1.90) |
| 0.75(0.27,1.90) | 0.45(0.20,1.00) | PemAxi | 0.19(0.10,0.47) | 1.00(0.17,17.00) | 0.42(0.18,0.97) | 0.50(0.23,0.84) |
| 4.00(1.11,30.00) | 2.30(1.16,11.00) | 5.10(1.26,31.00) | Placebo | 5.20(1.40,19.00) | 2.20(1.20,3.90) | 2.50(1.26,10.00) |
| 0.73(0.24,5.50) | 0.42(0.35,2.20) | 0.95(0.59,5.80) | 0.19(0.05,0.71) | Savo | 0.41(0.13,1.40) | 0.47(0.56,2.00) |
| 1.80(0.55,12.00) | 1.10(0.77,4.70) | 2.40(2.13,13.00) | 0.46(0.26,0.83) | 2.40(0.74,7.80) | Sora | 1.20(0.13,4.10) |
| 1.50(0.39,3.70) | 0.90(0.53,1.40) | 2.00(1.81,4.30) | 0.40(0.03,0.90) | 2.10(0.51,18.00) | 0.85(0.24,7.90) | Suni |

Abbreviations: Suni: Sunitinib; Cabo: Cabozantinib; Pazo: Pazopanib; Savo: Savolitinib; Sora: Sorafenib; Axi: Axitinib; Nivo: Nivolumab; PemAxi: Pembrolizumab plus Axitinib; AtezoBev: Atezolizumab plus Bevacizumab; AveAxi: Avelumab plus Axitinib; LenPem: Lenvatinib plus Pembrolizumab; NivoCabo: Nivolumab plus Cabozantinib; NivoIpi: Nivolumab plus lpilimumab; LenEvero: Lenvatinib plus Everolimus; IMA901Suni: IMA901 plus Sunitinib.

Yellow suggests a relative treatment benefit, blue suggests a relative treatment harm, dark red suggests not significant.

Supplementary Table 5C. Matrix of pairwise comparisons of regimens on objective response rate (shown as hazard ratio and 95% credible intervals). (meta-regression analysis)

| Anlo | 0.64(0.12,3.30) | 0.88(0.23,3.20) | 2.30(0.36,13.00) | 1.20(0.26,5.20) | 0.83(0.31,25.00) | 2.90(0.51,15.00) | 1.50(0.51,4.60) | 3.30(1.10,9.80) |
| --- | --- | --- | --- | --- | --- | --- | --- | --- |
| 1.60(0.30,8.50) | Atezo | 1.40(0.48,4.00) | 3.50(1.10,11.00) | 1.80(0.60,5.80) | 1.30(0.05,41.00) | 4.50(1.10,17.00) | 2.40(0.63,9.30) | 5.10(1.30,20.00) |
| 1.10(0.31,4.30) | 0.72(0.25,2.10) | AtezoBev | 2.60(0.91,7.10) | 1.30(0.55,3.30) | 0.95(0.38,27.00) | 3.3(1.00,11.00) | 1.70(0.64,4.80) | 3.70(1.40,10.00) |
| 0.44(0.74,2.80) | 0.28(0.90,0.89) | 0.39(0.14,1.10) | AveAxi | 0.51(0.18,1.60) | 0.37(0.13,12.00) | 1.30(0.35,47.00) | 0.68(0.17,2.90) | 1.40(0.35,6.20) |
| 0.86(0.19,3.80) | 0.55(0.17,1.70) | 0.76(0.30,1.80) | 1.90(0.64,5.50) | Axi | 0.72(0.30,19.00) | 2.50(0.68,8.60) | 1.30(0.40,4.20) | 2.80(0.85,8.90) |
| 1.20(0.41,32.00) | 0.76(0.25,20.00) | 1.10(0.04,26.00) | 2.70(0.085,75.00) | 1.40(0.53,33.00) | Bev | 3.40(0.11,97.00) | 1.80(0.07,46.00) | 3.90(0.14,98.00) |
| 0.35(0.66,1.90) | 0.22(0.57,0.87) | 0.31(0.11,1.00) | 0.79(0.21,2.90) | 0.41(0.12,1.50) | 0.29(0.10,9.30) | Cabo | 0.54(0.13,2.20) | 1.10(0.28,47.00) |
| 0.65(0.22,2.00) | 0.41(0.11,1.60) | 0.57(0.21,1.60) | 1.50(0.35,6.00) | 0.76(0.24,2.50) | 0.54(0.22,15.00) | 1.90(0.45,7.50) | LenEvero | 2.10(0.78,5.80) |
| 0.31(0.10,0.93) | 0.20(0.50,0.75) | 0.27(0.11,0.74) | 0.69(0.16,2.90) | 0.36(0.11,1.20) | 0.26(0.10,7.40) | 0.87(0.21,3.60) | 0.47(0.17,1.30) | LenPem |
| 1.70(0.05,51.00) | 1.10(0.01,1.10) | 1.50(0.18,1.10) | 3.80(0.026,4.80) | 2.00(0.20,1.70) | 1.40(0.80,2.50) | 4.80(0.043,4.60) | 2.60(0.47,1.20) | 5.50(0.10,2.60) |
| 0.42(0.15,1.20) | 0.27(0.04,1.80) | 0.37(0.76,17.00) | 0.96(0.11,7.50) | 0.49(0.85,2.80) | 0.35(0.12,11.00) | 1.20(0.17,8.10) | 0.65(0.18,2.30) | 1.40(0.38,4.90) |
| 1.10(0.36,27.00) | 0.68(0.65,59.00) | 0.93(0.12,58.00) | 2.40(0.17,2.60) | 1.20(0.04,93.00) | 0.88(0.57,1.40) | 3.00(0.30,2.50) | 1.60(0.34,65.00) | 3.50(0.07,1.40) |
| 0.95(0.36,2.10) | 0.60(0.13,2.40) | 0.83(0.27,2.20) | 2.10(0.41,9.60) | 1.10(0.32,3.60) | 0.78(0.33,21.00) | 2.70(0.58,11.00) | 1.50(0.54,3.40) | 3.10(1.20,7.20) |
| 0.59(0.24,1.40) | 0.38(0.74,1.80) | 0.52(0.15,17.00) | 1.30(0.22,7.50) | 0.68(0.17,2.90) | 0.49(0.19,14.00) | 1.70(0.32,8.50) | 0.90(0.33,2.50) | 1.90(0.70,5.30) |
| 12.00(3.10,42.00) | 7.40(1.80,29.00) | 10.00(3.20,30.00) | 26.00(5.90,11.00) | 14.00(1.49,37.00) | 9.70(0.47,2.20) | 33.00(7.40,14.00) | 18.00(5.40,55.00) | 38.00(1.10,12.00) |
| 1.10(0.16,74.00) | 0.68(0.14,3.20) | 0.94(0.23,3.80) | 2.40(0.54,10.00) | 1.20(0.28,5.50) | 0.89(0.28,31.00) | 3.10(0.57,16.00) | 1.60(0.32,8.50) | 3.50(0.66,18.00) |
| 1.90(0.44,8.30) | 1.20(0.35,4.10) | 1.70(0.61,4.40) | 4.30(1.30,14.00) | 2.20(1.30,3.90) | 1.60(0.68,40.00) | 5.40(1.40,20.00) | 2.90(0.88,9.50) | 6.20(1.90,20.00) |
| 1.30(0.55,3.20) | 0.85(0.28,2.50) | 1.20(0.59,2.30) | 3.00(0.89,97.00) | 1.50(0.63,3.90) | 1.10(0.48,29.00) | 3.80(1.10,12.00) | 2.00(1.08,4.10) | 4.30(2.10,8.90) |

Supplementary Table 5C (continued)

| 0.60(0.20,20.00) | 2.40(0.84,6.60) | 0.95(0.37,28.00) | 1.10(0.47,27.00) | 1.70(0.69,4.10) | 0.08(0.24,0.33) | 0.94(0.13,6.40) | 0.53(0.12,2.30) | 0.76(0.31,1.80) |
| --- | --- | --- | --- | --- | --- | --- | --- | --- |
| 0.93(0.01,1.10) | 3.70(0.55,26.00) | 1.50(0.17,1.50) | 1.70(0.41,7.50) | 2.70(0.55,14.00) | 0.13(0.35,0.56) | 1.50(0.31,7.00) | 0.83(0.25,2.90) | 1.20(0.39,3.60) |
| 0.68(0.03,56.00) | 2.70(0.57,13.00) | 1.10(0.17,82.00) | 1.20(0.45,37.00) | 1.90(0.57,6.70) | 0.09(0.03,0.31) | 1.10(0.26,4.40) | 0.60(0.23,1.60) | 0.86(0.44,1.70) |
| 0.26(0.21,39.00) | 1.00(0.13,9.00) | 0.42(0.38,58.00) | 0.47(0.10,2.40) | 0.75(0.13,4.50) | 0.03(0.01,0.17) | 0.42(0.95,1.80) | 0.23(0.07,0.79) | 0.33(0.10,1.10) |
| 0.51(0.59,50.00) | 2.00(0.36,12.00) | 0.81(0.11,73.00) | 0.91(0.28,3.20) | 1.50(0.35,6.00) | 0.07(0.03,0.21) | 0.80(0.18,3.50) | 0.46(0.26,0.78) | 0.65(0.26,1.60) |
| 0.71(0.40,1.20) | 2.80(0.10,83.00) | 1.10(0.73,1.70) | 1.30(0.48,31.00) | 2.00(0.10,53.00) | 0.10(0.46,2.10) | 1.10(0.32,35.00) | 0.63(0.25,15.00) | 0.90(0.34,21.00) |
| 0.21(0.22,23.00) | 0.82(0.12,5.80) | 0.33(0.40,33.00) | 0.37(0.88,17.00) | 0.59(0.12,3.10) | 0.03(0.01,0.14) | 0.33(0.63,17.00) | 0.19(0.05,0.71) | 0.26(0.08,0.88) |
| 0.39(0.81,21.00) | 1.50(0.43,5.60) | 0.62(0.15,30.00) | 0.69(0.30,1.80) | 1.10(0.40,3.10) | 0.56(0.02,0.19) | 0.61(0.12,3.20) | 0.34(0.10,1.10) | 0.49(0.24,1.00) |
| 0.18(0.38,9.80) | 0.72(0.20,2.60) | 0.29(0.72,14.00) | 0.32(0.14,0.87) | 0.52(0.19,14.00) | 0.02(0.01,0.09) | 0.29(0.55,1.50) | 0.16(0.05,0.54) | 0.23(0.11,0.47) |
| Nivo | 4.00(0.16,93.00) | 1.60(0.53,4.90) | 1.80(0.46,67.00) | 2.80(0.85,87.00) | 0.15(0.22,8.60) | 1.60(0.12,1.80) | 0.89(0.98,70.00) | 1.30(0.23,61.00) |
| 0.25(0.11,6.40) | NivoCabo | 0.40(0.21,8.70) | 0.45(0.17,1.30) | 0.72(0.27,1.90) | 0.03(0.01,0.17) | 0.40(0.45,3.40) | 0.22(0.04,1.20) | 0.32(0.10,0.97) |
| 0.63(0.21,1.90) | 2.50(0.11,47.00) | NivoLpi | 1.10(0.33,35.00) | 1.80(0.61,45.00) | 0.91(0.15,4.60) | 0.98(0.01,1.00) | 0.56(0.68,38.00) | 0.80(0.16,32.00) |
| 0.56(0.15,22.00) | 2.20(0.76,5.90) | 0.89(0.28,31.00) | Pazo | 1.60(0.68,3.30) | 0.08(0.03,0.21) | 0.88(0.15,4.90) | 0.50(0.15,1.60) | 071(0.36,1.20) |
| 0.35(0.11,12.00) | 1.40(0.54,3.70) | 0.56(0.22,16.00) | 0.62(0.31,1.50) | PemAxi | 0.05(0.02,0.18) | 0.55(0.84,3.60) | 0.31(0.76,1.30) | 0.45(0.20,0.96) |
| 6.90(0.12,4.50) | 27.00(1.60,12.00) | 11.00(0.22,6.50) | 12.00(1.47,34.00) | 20.00(5.50,68.00) | Placebo | 11.00(2.00,59.00) | 6.20(2.40,15.00) | 8.80(3.40,22.00) |
| 0.64(0.55,82.00) | 2.50(0.29,22.00) | 1.00(0.98,1.20) | 1.10(0.20,6.70) | 1.80(0.28,12.00) | 0.11(0.017,0.50) | Savo | 0.56(0.12,2.70) | 0.81(0.18,3.50) |
| 1.10(0.14,1.00) | 4.50(0.82,25.00) | 1.80(0.26,1.50) | 2.00(0.64,6.90) | 3.20(0.79,13.00) | 0.16(0.068,0.41) | 1.80(0.38,8.40) | Sora | 1.40(0.56,3.60) |
| 0.79(0.16,43.00) | 3.10(1.02,9.90) | 1.30(0.31,61.00) | 1.40(0.80,2.80) | 2.20(1.04,4.90) | 0.11(0.046,0.30) | 1.20(0.29,5.40) | 0.70(0.27,1.80) | Suni |

Abbreviations: Suni: Sunitinib; Bev: Bevacizumab; Cabo: Cabozantinib; Pazo: Pazopanib; Savo: Savolitinib; Sora: Sorafenib; Axi: Axitinib; Anlo: Anlotinib; Nivo: Nivolumab; Atezo: Atezolizumab; PemAxi: Pembrolizumab plus Axitinib; AtezoBev: Atezolizumab plus Bevacizumab; AveAxi: Avelumab plus Axitinib; LenPem: Lenvatinib plus Pembrolizumab; NivoCabo: Nivolumab plus Cabozantinib; NivoIpi: Nivolumab plus lpilimumab; LenEvero: Lenvatinib plus Everolimus. Yellow suggests a relative treatment benefit, blue suggests a relative treatment harm, dark red suggests not significant.

Supplementary Table 5D. Matrix of pairwise comparisons of regimens on grade ≥3 adverse events (shown as hazard ratio and 95% credible intervals). (meta-regression analysis).

| Anlo | 0.35(0.12,9.01) | 1.10(0.67,18.01) | 2.50(0.87,83.01) | 4.80(0.19,1.10) | 10.00(0.94,98.00) | 7.70(0.71,91.00) | 7.20(0.68,85.00) | 13.00(0.34,1.80) |
| --- | --- | --- | --- | --- | --- | --- | --- | --- |
| 2.90(0.11,82.20) | Atezo | 3.10(0.45,22.01) | 7.60(0.19,2.62) | 14.00(0.15,1.20) | 29.00(0.76,1.40) | 23.00(1.60,3.00) | 21.00(1.50,2.80) | 36.00(0.16,8.80) |
| 0.93(0.57,15.01) | 0.32(0.45,2.21) | AtezoBev | 2.50(0.83,56.00) | 4.40(0.62,30.00) | 9.40(0.39,2.60) | 7.50(0.80,56.00) | 7.00(0.75,53.00) | 12.00(0.80,1.60) |
| 0.39(0.12,11.02) | 0.13(0.38,5.32) | 0.41(0.18,12.02) | Axi | 1.80(0.06,67.00) | 4.00(0.98,1.40) | 3.00(0.12,79.00) | 2.80(0.11,73.00) | 5.30(0.40,2.30) |
| 0.21(0.90,5.33) | 0.72(0.80,0.69) | 0.22(0.34,1.61) | 0.55(0.15,18.00) | Cabo | 2.10(0.10,92.00) | 1.70(0.13,20.00) | 1.60(0.12,19.00) | 2.60(0.14,5.10) |
| 0.10(0.10,1.10) | 0.35(0.69,1.31) | 0.11(0.38,2.60) | 0.25(0.72,10.01) | 0.48(0.01,16.00) | IMA901Suni | 0.77(0.53,12.00) | 0.72(0.50,11.00) | 1.40(0.57,8.90) |
| 0.13(0.11,14.00) | 0.44(0.34,0.62) | 0.13(0.18,1.31) | 0.33(0.13,8.51) | 0.60(0.50,7.90) | 1.30(0.10,19.00) | LenEvero | 0.94(0.11,8.00) | 17.00(0.22,6.80) |
| 0.14(0.12,1.50) | 0.47(0.35,0.67) | 0.14(0.19,1.39) | 0.35(0.01,9.00) | 0.64(0.53,8.40) | 1.40(0.90,20.00) | 1.10(0.12,9.00) | LenPem | 1.80(0.24,7.60) |
| 0.75(5.40,29.03) | 0.28(1.10,61.01) | 0.85(0.62,1.31) | 0.19(0.43,2.50) | 0.38(2.00,7.30) | 0.74(0.11,1.70) | 0.60(0.15,4.50) | 0.56(0.13,4.20) | Nivo |
| 0.99(0.11,1.00) | 0.35(0.76,1.21) | 0.11(0.00,2.40) | 0.25(0.76,9.70) | 0.48(0.12,15.00) | 1.00(0.12,8.50) | 0.77(0.55,11.00) | 0.72(0.53,10) | 1.40(0.55,9.90) |
| 0.42(3.2,12.00) | 0.16(0.63,26.01) | 0.48(3.40,54.05) | 0.11(0.26,1.10) | 0.22(1.10,3.20) | 0.41(0.01,69.00) | 0.34(0.78,1.90) | 0.32(0.75,1.80) | 0.56(0.11,2.90) |
| 0.18(0.20,1.50) | 0.61(0.54,0.70) | 0.19(0.30,1.30) | 0.45(0.31,6.50) | 0.84(0.81,8.80) | 1.80(0.15,20.00) | 1.40(0.21,8.80) | 1.30(0.20,8.30) | 2.30(0.36,8.90) |
| 0.38(0.56,2.80) | 0.13(0.65,2.61) | 0.41(0.37,4.91) | 0.97(0.45,26.20) | 1.80(0.10,32.00) | 3.90(0.50,30.00) | 3.00(0.41,25.00) | 2.80(0.38,24.00) | 5.10(0.15,8.10) |
| 2.40(0.16,32.00) | 0.82(0.47,15.10) | 2.51(0.24,32.00) | 6.10(0.70,53.00) | 11.00(0.69,1.90) | 24.00(1.30,4.20) | 18.00(1.60,2.10) | 17.00(1.50,2.00) | 32.00(0.37,1.30) |
| 5.10(0.18,17.00) | 1.80(0.17,20.02) | 5.60(0.67,49.05) | 14.00(0.31,5.30) | 25.00(0.22,2.70) | 51.00(1.20,2.90) | 41.00(2.50,6.30) | 38.00(2.30,6.00) | 64.00(0.27,1.60) |
| 0.59(0.26,12.00) | 0.20(0.82,5.61) | 0.61(0.39,12.13) | 1.50(0.32,7.10) | 2.70(0.12,72.00) | 6.00(0.21,1.50) | 4.50(0.26,79.00) | 4.30(0.25,76.00) | 7.90(0.75,3.30) |
| 0.30(0.42,2.20) | 0.10(0.14,0.81) | 0.32(0.85,1.41) | 0.79(0.43,14.00) | 1.40(0.21,10.00) | 3.00(0.30,34.00) | 2.40(0.51,11.00) | 2.20(0.48,10.00) | 3.80(0.60,1.90) |

Supplementary Table 5D (continued)

| 10.00(0.98,94.00) | 24.00(0.10,3.10) | 5.60(0.69,50.00) | 2.60(0.35,18.00) | 0.42(0.31,6.30) | 0.19(0.60,5.70) | 17.00(0.83,38.00) | 3.30(0.46,24.00) |
| --- | --- | --- | --- | --- | --- | --- | --- |
| 29.00(0.81,1.30) | 64.00(0.38,1.60) | 16.00(1.40,1.90) | 7.40(0.39,1.50) | 1.20(0.65,21.00) | 0.55(0.51,5.90) | 5.00(0.18,1.20) | 9.60(1.2,72.00) |
| 9.40(0.42,2.30) | 21.00(0.02,3.00) | 5.40(0,77,33.00) | 2.50(0.21,27.00) | 0.40(0.32,4.20) | 0.18(0.20,1.50) | 1.60(0.82,25.00) | 3.10(072,12.00) |
| 4.00(0.10,1.30) | 9.50(0.01,3.90) | 2.20(0.15,32.00) | 1.00(0.38,22.00) | 0.16(0.19,1.40) | 0.73(0.19,3.20) | 0.67(0.14,3.10) | 1.30(0.73,23.00) |
| 2.10(0.67,83.00) | 4.60(0.31,9.40) | 1.20(0.11,12.00) | 0.54(0.31,9.80) | 0.89(0.52,1.40) | 0.40(0.37,0.45) | 0.36(0.14,8.40) | 0.70(0.98,4.80) |
| 0.99(0.12,8.50) | 2.40(0.14,1.50) | 0.55(0.49,6.70) | 0.26(0.33,2.00) | 0.41(0.24,0.80) | 0.20(0.34,0.82) | 0.17(0.67,4.80) | 0.33(0.30,3.30) |
| 1.30(0.01,18.00) | 2.90(0.01,1.30) | 0.73(0.11,4.70) | 0.34(0.39,2.50) | 0.54(0.48,0.62) | 0.24(0.16,0.40) | 0.22(0.13,3.80) | 0.42(0.94,1.90) |
| 1.40(0.02,19.00) | 3.20(0.01,1.30) | 0.78(0.12,5.00) | 0.36(0.42,2.60) | 0.58(0.51,0.65) | 0.26(0.17,0.43) | 0.23(0.13,4.10) | 0.45(0.99,2.10) |
| 0.73(0.10,1.80) | 1.80(0.35,9.20) | 0.43(0.11,2.80) | 0.20(0.12,67.00) | 0.31(0.78,27.00) | 0.16(0.61,37.00) | 0.13(0.30,1.30) | 0.26(0.52,1.70) |
| NivoCabo | 2.40(0.14,1.60) | 0.55(0.54,6.20) | 0.26(0.35,1.90) | 0.41(0.25,0.77) | 0.20(0.37,0.78) | 0.17(0.71,4.60) | 0.33(0.33,3.10) |
| 0.41(0.62,73.00) | Nivolpi | 0.24(0.62,1.10) | 0.11(6.90,27.00) | 0.18(4.60,11.00) | 0.88(3.3,16.00) | 0.71(1.8,57.00) | 0.15(2.7,68.00) |
| 1.80(0.16,19.00) | 4.10(0.17,1.60) | Pazo | 0.47(0.75,2.40) | 0.74(0.16,0.35) | 0.34(0.25,0.46) | 0.30(0.34,2.60) | 0.58(0.19,1.80) |
| 3.90(0.52,28.00) | 9.00(0.37,1.40) | 2.10(0.41,13.00) | PemAxi | 0.16(0.17,1.80) | 0.75(0.31,17.00) | 0.65(0.44,12.00) | 1.30(0.29,5.80) |
| 25.00(1.30,3.90) | 57.00(0.11,2.20) | 13.00(2.8,64.00) | 6.30(0.55,59.00) | Placebo | 0.45(0.23,9.70) | 4.10(0.89,18.00) | 7.80(1.2,53.00) |
| 51.00(0.13,2.70) | 1.10(0.64,3.00) | 30.00(2.20,4.00) | 13.00(0.60,3.20) | 2.20(0.10,44.00) | Savo | 9.00(0.29,2.50) | 17.00(0.18,1.60) |
| 6.00(0.22,1.40) | 14.00(0.17,5.60) | 3.30(0.38,29.00) | 1.50(0.86,23.00) | 0.25(0.055,1.10) | 0.11(0.40,3.40) | Sora | 1.90(0.17,22.00) |
| 3.10(0.33,30.00) | 6.80(0.15,3.70) | 17.00(0.57,5.20) | 0.79(0.17,3.4.00) | 0.13(0.19,0.86) | 0.59(0.62,0.56) | 0.52(0.45,5.90) | Suni |

Abbreviations: Suni: Sunitinib; Cabo: Cabozantinib; Pazo: Pazopanib; Savo: Savolitinib; Sora: Sorafenib; Axi: Axitinib; Anlo: Anlotinib; Nivo: Nivolumab; Atezo: Atezolizumab; PemAxi: Pembrolizumab plus Axitinib; AtezoBev: Atezolizumab plus Bevacizumab; LenPem: Lenvatinib plus Pembrolizumab; NivoCabo: Nivolumab plus Cabozantinib; NivoIpi: Nivolumab plus lpilimumab; LenEvero: Lenvatinib plus Everolimus; IMA901Suni: IMA901 plus Sunitinib. Yellow suggests a relative treatment benefit, blue suggests a relative treatment harm, dark red suggests not significant.

Supplementary Figure 7A. Convergence of the three Markov Chain Monte Carlo chains established by of the history feature for progression free survival. (meta-regression analysis). Suni: Sunitinib; Cabo: Cabozantinib; Pazo: Pazopanib; Savo: Savolitinib; Sora: Sorafenib; Axi: Axitinib; Nivo: Nivolumab; Pem_Axi: Pembrolizumab plus Axitinib; Atezo_Bev: Atezolizumab plus Bevacizumab; Ave_Axi: Avelumab plus Axitinib; Len_Pem: Lenvatinib plus Pembrolizumab; Nivo_Cabo: Nivolumab plus Cabozantinib; Nivo_Ipi: Nivolumab plus lpilimumab; Len_Evero: Lenvatinib plus Everolimus; IMA901_Suni: IMA901 plus Sunitinib.

Supplementary Figure 7B. Convergence of the three Markov Chain Monte Carlo chains established by of the history feature for overall survival. (meta-regression analysis). Suni: Sunitinib; Cabo: Cabozantinib; Pazo: Pazopanib; Savo: Savolitinib; Sora: Sorafenib; Axi: Axitinib; Nivo: Nivolumab; Pem_Axi: Pembrolizumab plus Axitinib; Atezo_Bev: Atezolizumab plus Bevacizumab; Ave_Axi: Avelumab plus Axitinib; Len_Pem: Lenvatinib plus Pembrolizumab; Nivo_Cabo: Nivolumab plus Cabozantinib; Nivo_Ipi: Nivolumab plus lpilimumab; Len_Evero: Lenvatinib plus Everolimus; IMA901_Suni: IMA901 plus Sunitinib.

Supplementary Figure 7C. Convergence of the three Markov Chain Monte Carlo chains established by of the history feature for objective response rate. (meta-regression analysis). Suni: Sunitinib; Bev: Bevacizumab; Cabo: Cabozantinib; Pazo: Pazopanib; Savo: Savolitinib; Sora: Sorafenib; Axi: Axitinib; Anlo: Anlotinib; Nivo: Nivolumab; Atezo: Atezolizumab; Pem_Axi: Pembrolizumab plus Axitinib; Atezo_Bev: Atezolizumab plus Bevacizumab; Ave_Axi: Avelumab plus Axitinib; Len_Pem: Lenvatinib plus Pembrolizumab; Nivo_Cabo: Nivolumab plus Cabozantinib; Nivo_Ipi: Nivolumab plus lpilimumab; Len_Evero: Lenvatinib plus Everolimus.

Supplementary Figure 7D. Convergence of the three Markov Chain Monte Carlo chains established by of the history feature for grade ≥3 adverse events. (meta-regression analysis). Suni: Sunitinib; Cabo: Cabozantinib; Pazo: Pazopanib; Savo: Savolitinib; Sora: Sorafenib; Axi: Axitinib; Anlo: Anlotinib; Nivo: Nivolumab; Atezo: Atezolizumab; Pem_Axi: Pembrolizumab plus Axitinib; Atezo_Bev: Atezolizumab plus Bevacizumab; Len_Pem: Lenvatinib plus Pembrolizumab; Nivo_Cabo: Nivolumab plus Cabozantinib; Nivo_Ipi: Nivolumab plus lpilimumab; Len_Evero: Lenvatinib plus Everolimus; IMA901_Suni: IMA901 plus Sunitinib.

Supplementary Figure 8A. Convergence of the three Markov Chain Monte Carlo chains established by of the Brooks-Gelman-Rubin diagnostic for progression free survival. (meta-regression analysis). Suni: Sunitinib; Cabo: Cabozantinib; Pazo: Pazopanib; Savo: Savolitinib; Sora: Sorafenib; Axi: Axitinib; Nivo: Nivolumab; Pem_Axi: Pembrolizumab plus Axitinib; Atezo_Bev: Atezolizumab plus Bevacizumab; Ave_Axi: Avelumab plus Axitinib; Len_Pem: Lenvatinib plus Pembrolizumab; Nivo_Cabo: Nivolumab plus Cabozantinib; Nivo_Ipi: Nivolumab plus lpilimumab; Len_Evero: Lenvatinib plus Everolimus; IMA901_Suni: IMA901 plus Sunitinib.

Supplementary Figure 8B. Convergence of the three Markov Chain Monte Carlo chains established by of the Brooks-Gelman-Rubin diagnostic for overall survival. (meta-regression analysis). Suni: Sunitinib; Cabo: Cabozantinib; Pazo: Pazopanib; Savo: Savolitinib; Sora: Sorafenib; Axi: Axitinib; Nivo: Nivolumab; Pem_Axi: Pembrolizumab plus Axitinib; Atezo_Bev: Atezolizumab plus Bevacizumab; Ave_Axi: Avelumab plus Axitinib; Len_Pem: Lenvatinib plus Pembrolizumab; Nivo_Cabo: Nivolumab plus Cabozantinib; Nivo_Ipi: Nivolumab plus lpilimumab; Len_Evero: Lenvatinib plus Everolimus; IMA901_Suni: IMA901 plus Sunitinib.

Supplementary Figure 8C. Convergence of the three Markov Chain Monte Carlo chains established by of the Brooks-Gelman-Rubin diagnostic for objective response rate. (meta-regression analysis). Suni: Sunitinib; Bev: Bevacizumab; Cabo: Cabozantinib; Pazo: Pazopanib; Savo: Savolitinib; Sora: Sorafenib; Axi: Axitinib; Anlo: Anlotinib; Nivo: Nivolumab; Atezo: Atezolizumab; Pem_Axi: Pembrolizumab plus Axitinib; Atezo_Bev: Atezolizumab plus Bevacizumab; Ave_Axi: Avelumab plus Axitinib; Len_Pem: Lenvatinib plus Pembrolizumab; Nivo_Cabo: Nivolumab plus Cabozantinib; Nivo_Ipi: Nivolumab plus lpilimumab; Len_Evero: Lenvatinib plus Everolimus.

Supplementary Figure 8D. Convergence of the three Markov Chain Monte Carlo chains established by of the Brooks-Gelman-Rubin diagnostic for grade ≥3 adverse events. (meta-regression analysis). Suni: Sunitinib; Cabo: Cabozantinib; Pazo: Pazopanib; Savo: Savolitinib; Sora: Sorafenib; Axi: Axitinib; Anlo: Anlotinib; Nivo: Nivolumab; Atezo: Atezolizumab; Pem_Axi: Pembrolizumab plus Axitinib; Atezo_Bev: Atezolizumab plus Bevacizumab; Len_Pem: Lenvatinib plus Pembrolizumab; Nivo_Cabo: Nivolumab plus Cabozantinib; Nivo_Ipi: Nivolumab plus lpilimumab; Len_Evero: Lenvatinib plus Everolimus; IMA901_Suni: IMA901 plus Sunitinib.
